# Thirty years of declining stunting in Tanzania: Trends and ongoing challenges

**DOI:** 10.1371/journal.pone.0327779

**Published:** 2025-07-29

**Authors:** Ester Elisaria, Bet Caeyers, Esther Nkuba, Laura van der Erve, August Kuwawenaruwa

**Affiliations:** 1 Ifakara Health Institute, Dar es Salaam, Tanzania; 2 Chr. Michelsen Institute (CMI), Bergen, Norway; 3 Centre for Experimental Research on Fairness, Inequality and Rationality (FAIR) & Norwegian School of Economics; 4 Tanzania Food and Nutrition Center (TFNC), Dar-es-Salaam, Tanzania; 5 Oxford Policy Management, Oxford, United Kingdom; University of Waterloo, CANADA

## Abstract

**Background:**

Tanzania has made considerable gains in children’s nutrition between 1991/92 and 2022. The country’s stunting rate has decreased from 50% in 1992 to 30% in 2022. However, stunting varies greatly among regions. The purpose of this study was to examine regional trends in stunting rates and associated characteristics related to the risk of being stunted among children under the age of five in Tanzania.

**Methods:**

Descriptive statistics were used to summarize the data on stunting, from the Tanzania Demographic and Health Survey (TDHS) data. A total of 42,408 under-five children from repeated TDHS cross-sectional studies conducted in 1991–1992 (n = 6,585), 1996 (n = 5,438), 1999 (n = 2,555), 2004–2005 (n = 7,230), 2009–2010 (n = 6,792), 2015–2016 (n = 9,001), and 2022 (n = 4,807) were analysed to examine trends in stunting and its associated characteristics in the country. Frequencies and percentages were calculated and presented in tables and graphs as cross-sectional data points. A multivariable logistic regression model was estimated to identify factors associated with stunting in 2022. All analyses have been weighted using the weighting generated by the TDHS. STATA version 15 was used for data management and analysis.

**Results:**

Over the previous three decades, stunting rates in boys under five have consistently been 4-7 percentage points (ppt) higher than those in girls of the same age. The prevalence of stunted children was greater in households with the lowest socioeconomic status (SES) (38.6%) compared to those with the highest SES (12.6%). Similar to the trend analysis, a multivariable regression analysis found that residency in the Southern Highlands (AOR = 2.368; 95% CI: 1.746-3.212, p < 0.001), male children (AOR = 1.583 [95% CI: 1.349 - 1.858], p < 0.001), low birth weight (AOR = 3.639 [95% CI: 2.279 - 5.810], p < 0.001), maternal exposure to alcohol (AOR = 1.440 [95% CI: 1.057 – 1.963], p < 0.05), and unimproved sanitation facilities (AOR = 1.345 [95% CI: 1.055 – 1.714], p < 0.05) were significantly associated with a higher risk of stunting. In contrast, a birth interval of 24 to 47 months (AOR = 0.762 [95% CI: 0.598 - 0.969], p < 0.001), a high level of maternal education (AOR = 0.715 [95% CI: 0.530 – 0.963], p < 0.05), and high socioeconomic status (AOR = 0.268 [95% CI: 0.178 – 0.403], p < 0.001) were significantly associated with a lower risk of stunting.

**Conclusion:**

Although child stunting has decreased in the country, it remains a major challenge in Tanzania, driven by factors such as residing in the Southern Highlands, child and maternal issues, and household economic and environmental factors. To combat stunting and eliminate structural obstacles, including the empowerment of marginalised groups, a multisectoral strategy is required. Furthermore, current regulations and standards place more emphasis on educating mothers about diet and health than on empowering them economically.

## Introduction

In 2022, an estimated 148.1 million children under the age of five globally were classified as stunted [1]. The prevalence of stunting was notably higher among children residing in low- and lower-middle-income countries, which accounted for 89% of the global burden of stunting in 2020. Additionally, these children were more likely to come from rural areas and have mothers who had not received an education. Alarmingly, nearly one-third of countries in Northern Africa, Oceania, and the Caribbean are experiencing an increase in the number of stunted children [2]. This trend underscores a concerning lack of progress towards the goal of halving the prevalence of stunting by the year 2030. Central Africa is the most severely affected subregion, with a prevalence rate of 37.4% [3]. In the case of Tanzania, the Demographic and Health Surveys (DHS) data collected from 1991/92 to 2022 indicates the country had a decrease in stunting from 50% in 1991/92 to 30% in 2022 [2], with a significant disparity between rural and urban areas [4]. The World Bank and UNICEF agree that heightened efforts are needed to reach the global goal of reducing the number of children suffering from stunting to 89 million by 2030 [1].

Earlier research indicated that the disparity in stunting prevalence between rural and urban areas is linked to several factors, including access to public services, education, and economic resources [4–6]. The findings further demonstrated that understanding the underlying causes of stunting in different contexts can facilitate the development of tailored interventions for both urban and rural settings [4]. Stunting occurs due to inadequate nutrition during pre-conception, pregnancy, and early childhood [7–10]. Children affected by stunting may never reach their full potential height and may not fully develop their cognitive abilities. These children start life at a significant disadvantage, and the consequences persist into adulthood. They encounter challenges in learning at school, have lower earning potential as adults, and face obstacles to engaging in their communities [3]. Most Tanzanians have undiversified diets, where an average of 71% of all energy comes from staple foods. Even in the wealthiest segment of the population, nearly 60% of energy is derived solely from staple foods [11]. The 2022 TDHS indicates that only 64% of children aged less than six months were exclusively breastfed, only 19% of children aged 6–23 months, and 25% of women aged 15–49 years received a diversified diet. In addition, 11% of children aged 6–59 months were given iron-containing supplements in the last 12 months at the health facility, and 53% were given vitamin A supplements in the last 6 months.

To the best of our knowledge, none of the previous studies conducted in Tanzania [4,6,10,12–14] have thoroughly examined the time trends of stunting alongside child-related factors (such as birth weight, gender, and age), maternal factors (including breastfeeding practices, maternal education, nutritional status, and the number of antenatal care visits), household-related factors (such as hygiene practices, socio-economic status, the gender of the household head, and child infections, particularly diarrhoea in the 2 weeks before the survey), and cross-regional comparisons from 1991/92 to 2022. Therefore, the primary aim of this study was to examine the trends of stunting and characteristics associated with the risk of being stunted in Tanzania.

## Materials and methods

### Ethical considerations

This is a secondary analysis of the Tanzania Demographic Health Surveys collected by the government through the National Bureau of Statistics. Prior to data collection, the protocols and data collection procedures were approved by the relevant authorities in mainland Tanzania and Zanzibar. These included the National Institute of Medical Research (NIMR), the Zanzibar Medical Research Ethical Committee (ZAMREC), the Institutional Review Board of ICF International, and the Centres for Disease Control and Prevention in Atlanta. All participants provided written informed consent before the interviews. For illiterate individuals, a written consent form was presented orally in the presence of a legally permitted representative or witness, such as a friend, relative, or someone not affiliated with the research team. Parental or guardian consent was obtained for children and adolescents participating in the study. Every qualified respondent was questioned in the most private setting possible, without the presence of a third party.

### Study design

The DHS are nationally representative, repeated cross-sectional surveys conducted in low- and middle-income countries. Seven waves of Tanzania DHS have been conducted since 1991−92–2022.

### Data sources

The Demographic and Health Surveys provide valuable information for comparing nutritional and health estimates within and across countries, monitoring trends, and measuring the progress of interventions targeting stunting among children. Among other measures, these gather socio-economic and demographic data on children under the age of five, along with other health and development measures. The resulting data contain both national and regional-level estimates for stunting indicators, essential for making inferences about national and regional trends. All data presented in the graphs as cross-sectional data points and tables are from the Tanzania DHS, 1991/92–2022 [15–21].

Anthropometry is commonly used to measure a child’s nutritional status. Anthropometric measurements are used to report on child growth indicators. According to the World Health Organization (WHO) 2006 report, the distribution of height and weight among children under age 5 was compared with the WHO Child Growth Standards reference population [22]. The distribution of a well-nourished population will be similar to that of the reference population, while the distribution of a poorly nourished population will not. Children who are too short for their age are considered stunted, and this is the result of chronic or recurrent undernutrition. Stunting is the impaired growth and development that children experience due to poor nutrition, repeated infection, and inadequate psychosocial stimulation [23]. Children whose height-for-age z-score (HAZ) is below minus two standard deviations (−2 SD) from the median of the reference population are considered short for their age (stunted). Children whose HAZ is below minus three standard deviations (−3 SD) from the median are considered severely stunted [24,25]. HAZ is calculated by subtracting an age- and sex-appropriate median value from a standard population, and dividing it by the SD of the standard population.

The primary outcome of interest was stunting, defined as a height-for-age measurement that is more than two standard deviations below the mean of the reference population. In 2006, the WHO recommended new standard growth cut-off points for stunting [22]. The Tanzania DHS surveys conducted from 1991/92 to 2004/05 used the Center for Disease Control standard growth references, which were derived from the National Center for Health Statistics, Fels Research Institute, and Center for Disease Control reference population. To harmonize the surveys from 1991/2 to 2004/5, z-scores were recalculated according to the new WHO Child Growth Standards. The syntax file is available from the WHO (http://www.who.int/childgrowth/software/en/).

To maintaining the quality of measurements for the TDHS, quality control teams comprised personnel from the National Bureau of Statistics, the Office of the Chief Government Statistician, the Tanzania Food and Nutrition Centre (TFNC), and the ministry responsible for health in both mainland Tanzania and Zanzibar [26]. Monitoring of fieldwork involved regular visits to ensure the survey was conducted according to established protocols, providing real-time solutions to field challenges by observing the biomarker measurements of eligible respondents. All biomarker questionnaires and urine specimens were dispatched weekly to the nearest TFNC laboratory. Field check tables were regularly generated from Syncloud to monitor data quality and fieldwork progress. For field teams facing specific challenges, quality control staff provided targeted instructions to improve their performance; otherwise, consistent feedback was offered to all field teams.

### Data access

The use of this data was approved by the Demographic and Health Surveys Program, ICF, following our request with the data analysis concept. An authorisation letter was issued to the research team on 31 January 2024.

### TDHS sampling of households

The TDHS employed a random sampling method that took population density into account to gather data from all administrative regions of the country. Data of 42,408 under-five children had nutrition related variables. The seven waves of TDHS had different sample size, 1991–1992 (n = 6,585), 1996 (n = 5,438), 1999 (n = 2,555), 2004–2005 (n = 7,230), 2009–2010 (n = 6,792), 2015–2016 (n = 9,001), and 2022 (n = 4,807).

### Data analysis

#### Descriptive analysis.

Descriptive statistics were used to summarize the data on stunting, while the factors influencing stunting (such as gender and age), caregiver/mother-related factors (such as education, number of ANC visits, and age at first birth), and household-level factors (such as gender of the household head and household wealth status) were analyzed using appropriate statistical methods for the Tanzania DHS 1991/92–2022 survey data. We calculated frequencies and percentages, which are displayed using graphs as cross-sectional data points. A chi-square test was used to assess the association between stunting and each of the independent variables for identifying factors associated with stunting. The descriptive statistics guided the selection of covariates included in the univariate and multivariable logistic regression model to assess the factors influencing stunting in 2022.

#### Inferential analysis.

It has been hypothesised that child stunting would be affected by both child, caregivers, and household-level factors. A multivariable logistic regression model was estimated to identify factors associated with stunting in 2022. Both backward and forward elimination have been used to arrive at the final model for statistically significant levels, conditional on the p-value being less than 5 percent (p < 0.05). All analyses have been weighted using the weighting generated by the TDHS. STATA (StataCorp, College Station, TX, USA) version 15 was used for data management and analysis. The ‘svy’ commands were utilised to account for the cluster sampling design, sampling weights, and the calculation of standard errors.

## Results

### Profile of the respondents 1991-92 to 2022

Table 1 presents information on the profile of the respondents from the repeated Tanzania DHS cross-sectional surveys conducted from 1991-92 to 2022. The findings indicated that across all seven waves of the Tanzania DHS, there was minimal variation in the proportion of male and female children included in the studies. Close to half of children represented in all surveys were aged between 18 and 47 months, ranging between 47.9% and 49.5% for all TDHS waves.

**Table 1:** Characteristics of children, caregivers, and households from 1991−92 to 2022.

	TDHS 1991−92 N = 6,585	TDHS 1996 N = 5,438	TDHS 1999 N = 2,555	TDHS 2004−05 N = 7,230	TDHS 2010 N = 6,792	TDHS 2015−16 N = 9,001	TDHS 2020 N = 4,807
	n(%)	n(%)	n(%)	n(%)	n(%)	n(%)	n(%)
Child Characteristics							
Child sex							
Male	3,254(49.4)	2,760(50.7)	1,290(50.5)	3,609(49.9)	3,374(49.7)	4,508(50.1)	2,426(50.5)
Female	3,331(50.6)	2,678(49.3)	1,265(49.5)	3,621(50.1)	3,418(50.3)	4,493(49.9)	2,381(49.5)
Child age, months							
<9	1,154(17.5)	949(17.4)	466(18.2)	1,253(17.3)	1,107(16.3)	1,472(16.5)	760(15.8)
9 - 17	1,180(17.9)	968(17.7)	419(16.4)	1,264(17.5)	1,118(16.5)	1,560(17.5)	775(16.1)
18–47	3,155(47.9)	2,607(47.9)	1,227(48.0)	3,463(47.9)	3,361(49.5)	4,324(48.4)	2,365(49.2)
48–59	1,096(16.6)	914(16.8)	443(17.3)	1,250(17.3)	1,206(17.7)	1,575(17.6)	876(18.2)
Maternal characteristics							
Mother’s Education							
None	2,488(37.8)	1,586(29.2)	706(27.6)	1,982(27.4)	1,735(25.5)	1,964(21.8)	1,033(21.5)
Primary	3,882(58.9)	3,582(66.0)	1,546(60.5)	4,641(64.2)	4,281(63.0)	5,411(60.1)	2,522(52.5)
Secondary and above	215(3.3)	258(4.7)	303(11.8)	607(8.4)	776(11.4)	1,626(18.1)	1,252(26.0)
Body mass index (kg/m2) of mother							
Underweight (< 18.50)	610(9.3)	472(8.7)		641(8.9)	664(9.8)	642(7.1)	332(6.9)
Normal (18.50–24.99)	5,213(79.5)	4,197(77.9)		5481(76.1)	4,787(70.7)	6,057(67.5)	3,031(63.1)
Overweight (>=25.0)	731(11.1)	717(13.3)		1,085(15.0)	1321(19.5)	2,280(25.4)	1,435(29.8)
ANC visits							
< 4 visits	2,165(32.9)	1,614(29.7)	1,301(50.9)	4,111(56.8)	4,735(69.7)	5,828(64.7)	3,043(63.3)
>= 4 visits	4,420(67.1)	3,824(70.3)	1,254(49.1)	3,119(43.1)	2,057(30.3)	3,173(35.3)	1,764(36.7)
Household Related Factors							
Gender of Head of Household							
Female	762(11.6)	842(15.5)	395(15.5)	6,075(15.9)	1,094(16.1)	1,420(15.8)	1,094(22.8)
Male	5,823(88.4)	4,596(84.5)	2,160(84.5)	1,155(84.0)	5,698(83.9)	7,581(84.2)	3,713(77.2)
Locality							
Rural	5,575(84.7)	4,376(80.5)	1,909(74.7)	6,001(83.0)	5,554(81.8)	6,966(77.4)	3,517(73.2)
Urban	1,010(15.3)	1,062(19.5)	646(25.3)	1,229(17.0)	1,238(18.2)	2,035(22.6)	1,290(26.8)
SES							
Lowest	1,952(30,4)	1,544(28.4)	930(36.4)	1,590(21.9)	1,389(20.4)	2,080(23.1)	1,029(21.4)
Second	615(9.6)	654(12.0)	211(8.3)	1,453(20.1)	1,561(22.9)	1,879(20.9)	951(19.8)
Middle	1,320(20.6)	1,469(27.0)	686(26.8)	1,463(20.2)	1,453(21.4)	1,756(19.5)	993(20.7)
Fourth	1,240(19.5)	698(12.8)	240(9.4)	1,605(22.2)	1,393(20.5)	1,854(20.6)	976(20.3)
Highest	1,278(19.9)	1,073(19.7)	480(19.1)	1,119(15.5)	996(14.7)	1,432(15.9)	858(17.8)

Maternal characteristics demonstrate a positive trend in educational attainment among caregivers. Over the past 30 years, the percentage of caregivers without any formal education has decreased from 37.8% in the 1991–1992 period to 21.5% in the 2022 survey. Similarly, the proportion of caregivers with secondary education or higher has risen from 3.3% in 1991–1992 to 26% in the 2022 survey. Across all the Tanzania DHS, the majority (> 63.1%) of caregivers had a normal weight (Body Mass Index [kg/m²] – 18.5 to 24.9).

Household-level characteristics indicate that the majority of household heads were male across all surveys, with proportions ranging from 77.2% to 88.4%. Additionally, most respondents in all surveys were sampled from rural areas, with percentages ranging from 73.2% to 84.7%.

### Trends of stunting in Tanzania

**Fig 1:**
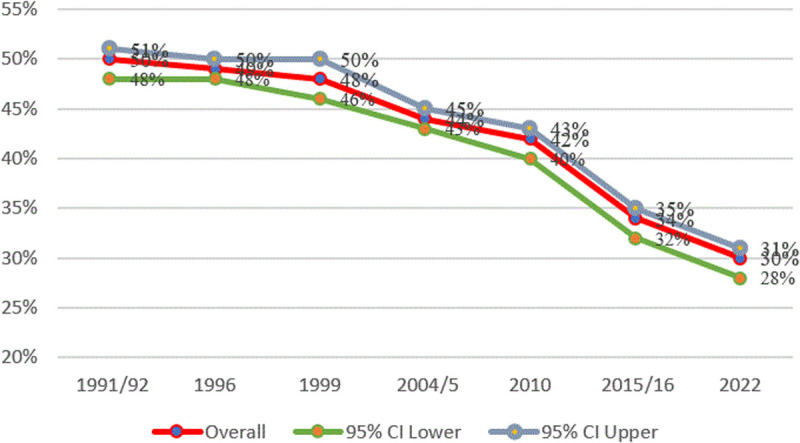
Prevalence of stunting from Tanzania Demographic Health Surveys 1991–2022. The red line shows the stunting rates, which is displaying a declining trend. The line graphs represent cross-sectional data points for different TDHS from 1991/92–2022, rather than longitudinal data for all the graphs.

In Tanzania, stunting has decreased slowly over the last 30 years, from 50% in 1991/92–30% in 2022 (Fig 1).

### Characteristics associated with the risk of stunting (child, mother, and family)

#### Stunting and child gender.

**Fig 2:**
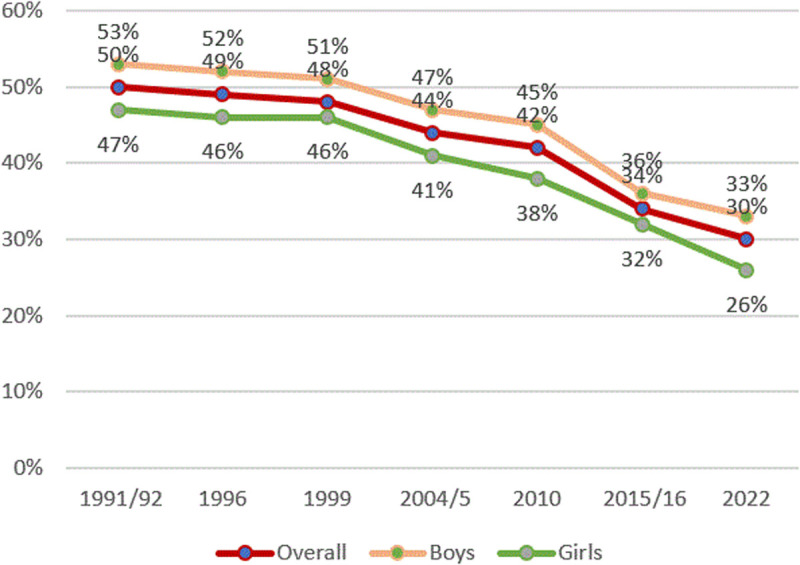
Prevalence of stunting and child gender from TDHS 1991/92–2022. The red line represents the overall stunting rates, the orange line represents stunting rates among boys, and the green line represents stunting rates among girls.

Fig 2 shows this disparity over time in Tanzania, where, over the last 30 years, under-five boys have consistently had 4–7 percentage points (ppts) higher stunting rates than girls of the same age. Girls have a 16% lower chance of being stunted. 

#### Stunting and child age.

**Fig 3:**
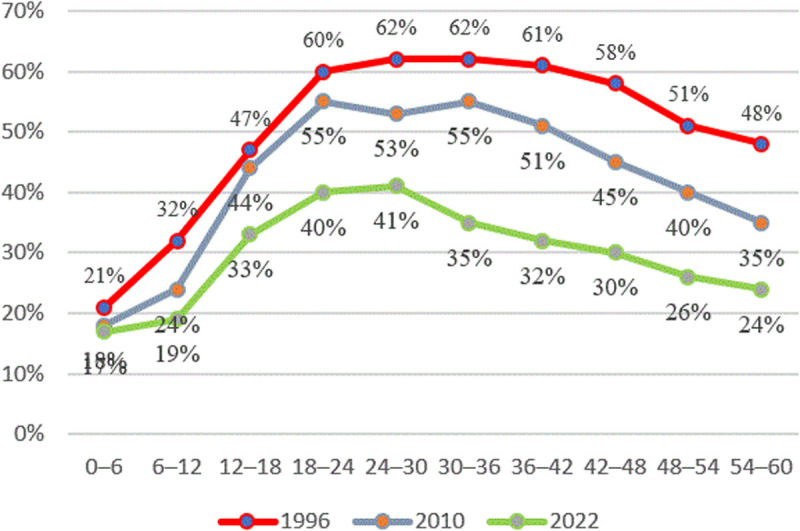
Prevalence of stunting and age in months from TDHS from 1991/92–2022. The red line represents the stunting rates for 1996, the grey line represents stunting rates for 2010, and the green line represents stunting rates for 2022.

Fig 3 highlights this pattern in Tanzania, where, until approximately two years, stunting rates increase and decrease thereafter. Stunting rates are currently at their highest in children of 18–24 months (40%) and 24–30 months (41%).

#### Stunting and child birthweight.

**Fig 4:**
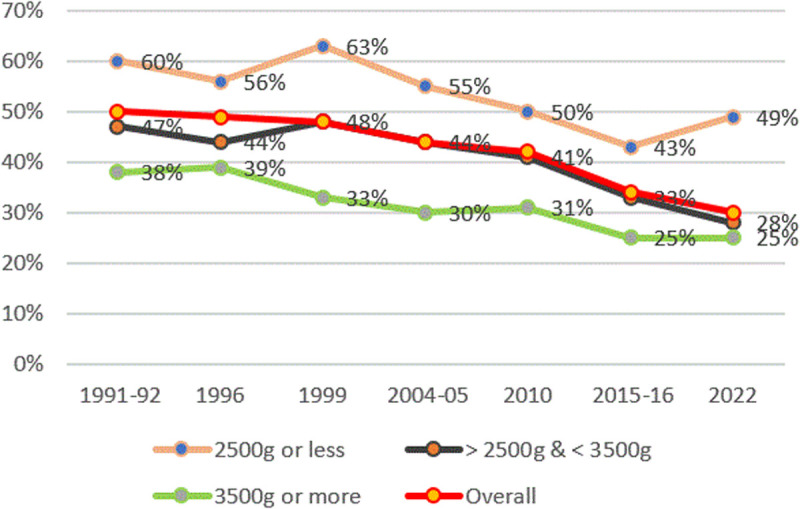
Prevalence of stunting and birthweight from TDHS 1991/92–2022. The red line represents overall stunting rates, black line for those with weights>2,500 grams & < 3,500 grams, the orange line represents stunting rates for those with weights less than 2,500 grams, and the green line represents stunting rates for those with weights above 3,500 grams.

Fig 4 shows the relationship between birthweight and stunting over time in Tanzania. Close to half of children under five years old who were born with low birthweight (<2,500 grams) are stunted, and only around a quarter of children who weighted 3,500 grams or more at birth.

### Maternal characteristics associated with the risk of stunting

#### Breastfeeding practices.

**Fig 5:**
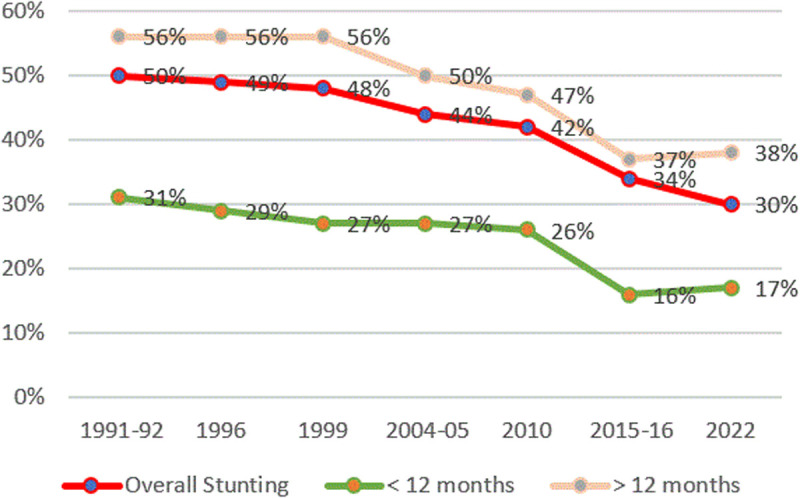
Prevalence of stunting and breastfeeding duration from TDHS 1991/92 to 2022. The red line represents the overall stunting rates, the orange line represents stunting rates for those who maintained breastfeeding for more than one year and above, the green line represents stunting rates for those with less than 12 months breastfeeding.

There has been a decline in the stunting trend for those breastfeeding for more than 12 months in Tanzania, from 51% in 1991/92 to 38% in the 1996 DHS, while stunting rates for those breastfeeding for less than 12 months declined by 5 ppts from 22% in 1991/92–17% in 2022 (Fig 5).

#### Mother’s education.

**Fig 6:**
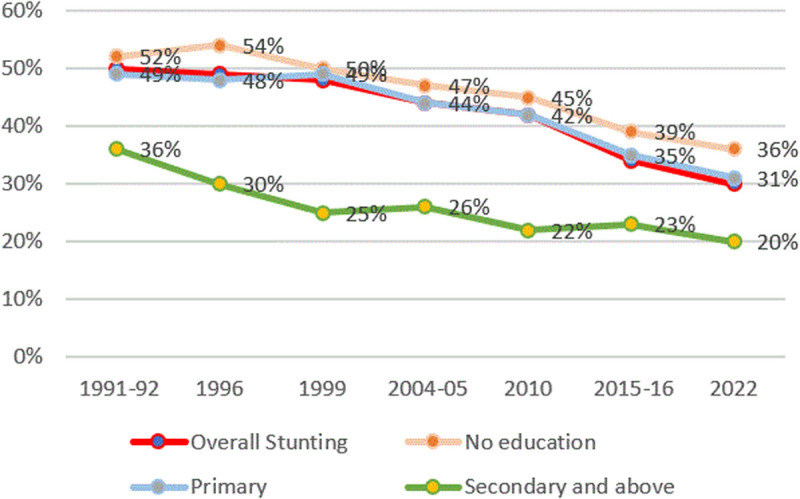
Prevalence of stunting and mother’s education from TDHS 1991/92–2022. The red line represents the overall stunting rates, the green line represents stunting rates for those who attained secondary education and above, the grey line represents stunting rates for those with primary education, and the orange line represents those who had not attended formal education.

Fig 6 shows the trend of stunting and maternal education in Tanzania. It is observed that mothers with no education have children with higher rates of stunting compared to those with higher education (primary complete). There has been a declining trend since the 1996 DHS for all education categories. For example, stunting among caregivers with no education declined by 11 ppts from 47% to 36% in the 2022 DHS.

#### Stunting and Mother’s nutritional status.

**Fig 7:**
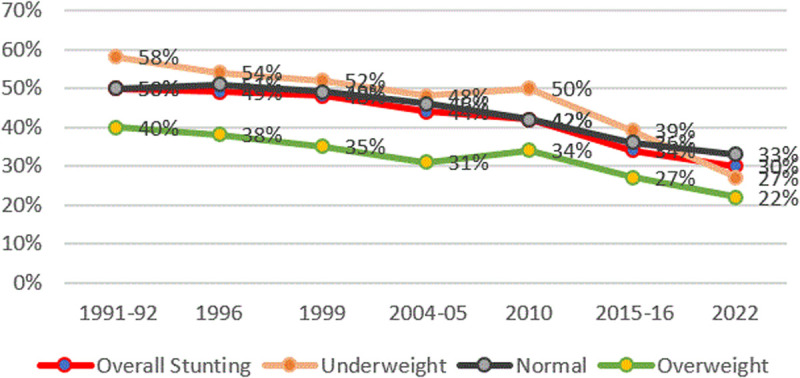
Prevalence of stunting and mothers’ nutritional status from TDHS 1991/92 to 2022. The red line represents the overall stunting rates, the black line presents stunting rates for mothers who have normal weight, the orange line represents stunting rates for overweight mothers, and the green line represents stunting rates for underweight mothers.

Mother’s nutritional characteristics in Tanzania across the years have been consistently closely correlated with the growth of the newborn (Fig 7). Mothers with a low body mass index have a higher proportion of stunted children.

#### Stunting and number of ANC visits.

**Fig 8:**
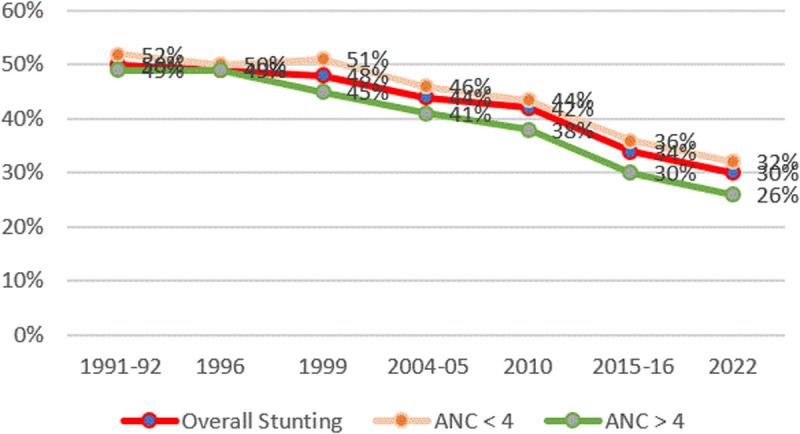
Prevalence of stunting and ANC visits from TDHS 1991/92–2022. The red line represents the overall stunting rates, the green line presents stunting rates for mothers who had more than 4 ANC visits, and the orange line represents stunting rates for mothers who had less than 4 ANC visits.

Fig 8 shows the trend of the ANC visits over time. An increased number of ANC visits to more than 4 has been associated with a declining trend of stunting. A 16-point decline in stunting was observed for mothers having more than 4 ANC visits, from 42% in 1991−92 to 26% in 2022.

### Household related factors

#### Stunting and access to improved water.

**Fig 9:**
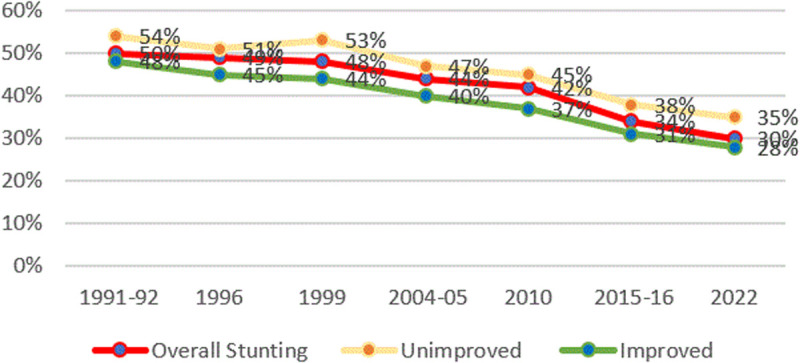
Prevalence of stunting and household access to water from TDHS from 1991/92 to 2022. The red line represents the overall stunting rates, the green line presents stunting rates for those with access to improved water, and the orange line represents stunting rates for those without access to improved water.

Fig 9 shows the trends in stunting and access to improved water. Stunting rates among households with access to unimproved water are higher than at the national level.

#### Stunting and hygiene practices.

**Fig 10:**
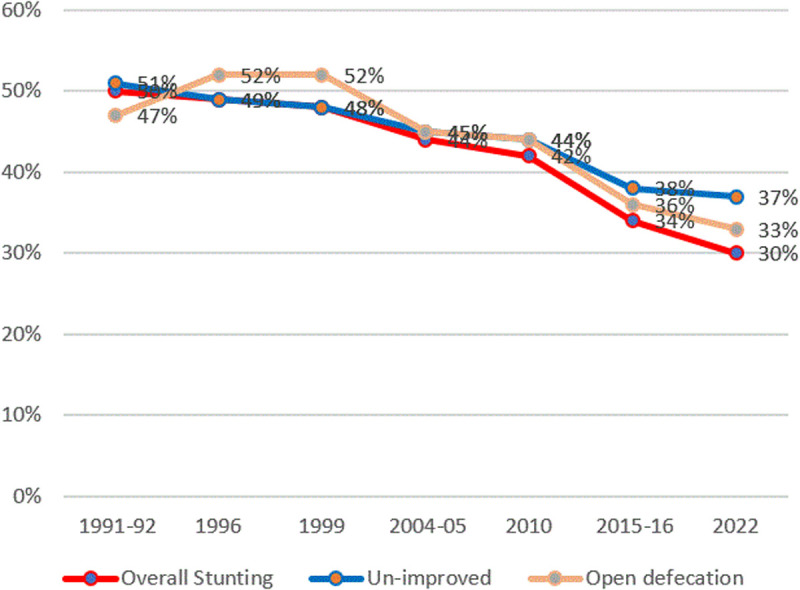
Prevalence of stunting and household access to sanitation facilities in Tanzania from the TDHS 1991/92 to 2022. The red line represents the average stunting rates, the blue line represents stunting rates for households with unimproved sanitation facilities, and the orange line represents stunting rates for households practicing open defecation.

Fig 10 shows the trends in stunting and household sanitation facilities. Stunting rates among households with unimproved sanitation and open defecation are higher than at the national level.

#### Stunting and Household Socioeconomic Status (SES).

**Fig 11:**
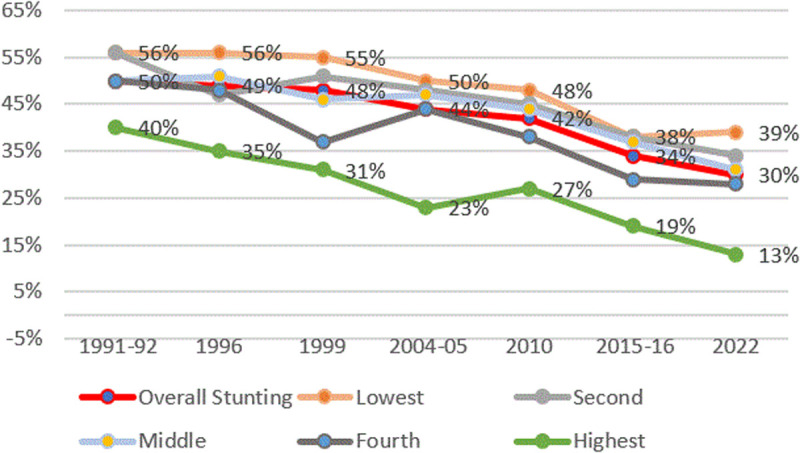
Prevalence of stunting and household ranking for each person using socioeconomic status from TDHS 1991/92 to 2022. The red line represents the average stunting rates, the orange line represents stunting rates for households within the lowest SES, and the green line represents stunting rates for households within the highest SES.

Fig 11 shows a clear disparity in stunting rates between households of different socioeconomic statuses (SES), with households in the highest wealth quintile having lower stunting rates than those in the lowest wealth quintile.

#### Stunting and gender of head of household.

**Fig 12:**
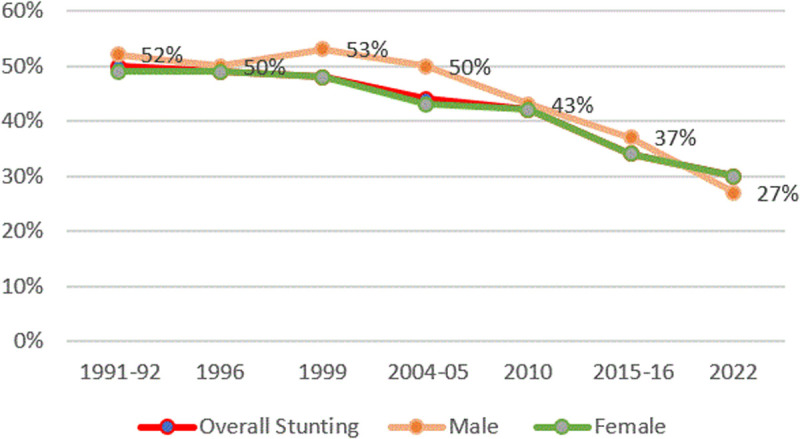
Prevalence of stunting and male vs female household head from TDHS 1991/92 to 2022. The red line represents the average stunting rates, the green line represents stunting rates for female-headed, the orange line represents stunting rates for male headed households.

There is a declining stunting trend within male-headed households from 43% in 1991 to 30% in 2022. The proportion of stunting within female-headed households has been higher than that of male-headed households from 1991−2 to 2015−16 (Fig 12).

#### Stunting and child infection (Diarrhoea).

**Fig 13:**
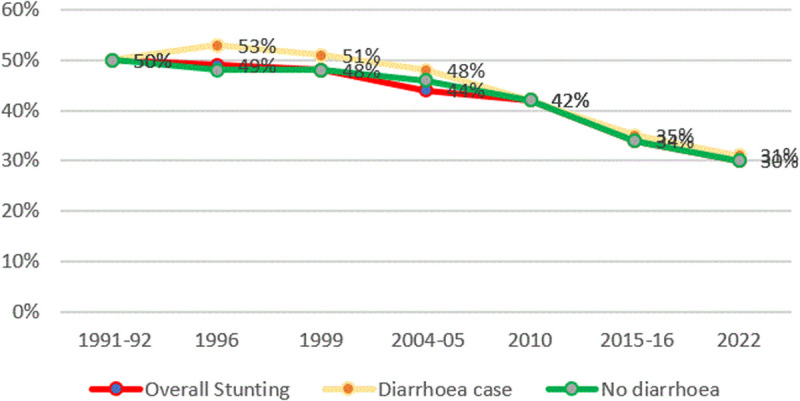
Prevalence of stunting and child diarrhoea from TDHS 1991/92 to 2022. The red line represents the average stunting rates, the green line represents stunting rates for child who had no diarrhoea, and the orange line represents stunting rates for child who had diarrhoea.

The prevalence of diarrhoea among children under 5 declined from 31% and 13% in 1991 to 8% and 9% respectively in 2022 in the 2 weeks before the survey. It has been observed that as diarrhoea declines over the years, stunting also decreases. Trends of stunting and diarrhoea are presented in Fig 13. Repeated episodes of sickness deteriorate a child’s growth over time.

### Stunting by regions

**Fig 14:**
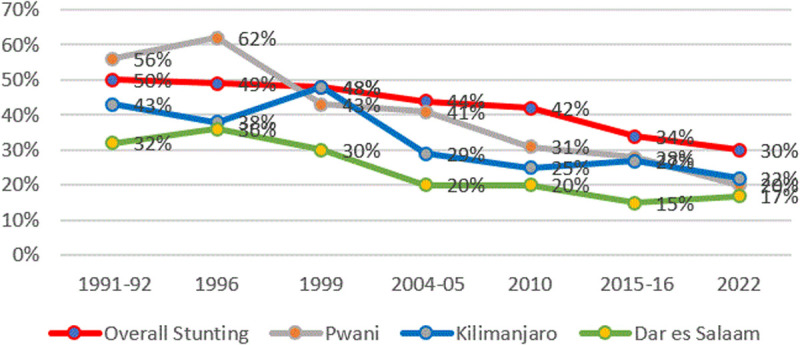
Prevalence of stunting and regions with low stunting rates from TDHS 1991/92–2022. The red line represents the average stunting rates, the green line represents stunting rates for the Dar es Salaam region, the blue line represents stunting rates for the Kilimanjaro region, and the grey line represents stunting rates for the Pwani region.

Fig 14 presents information on time trends in stunting in a few selected regions that were performing relatively well in 2022 (Dar es Salaam, Pwani, Kilimanjaro). Interestingly, whereas Dar es Salaam and Kilimanjaro have consistently been outperforming the average region in Tanzania from 1992–2022 in terms of stunting rates, Pwani started off as a relatively poor performer in 1992 but over time has become a relatively well-performing region.

**Fig 15:**
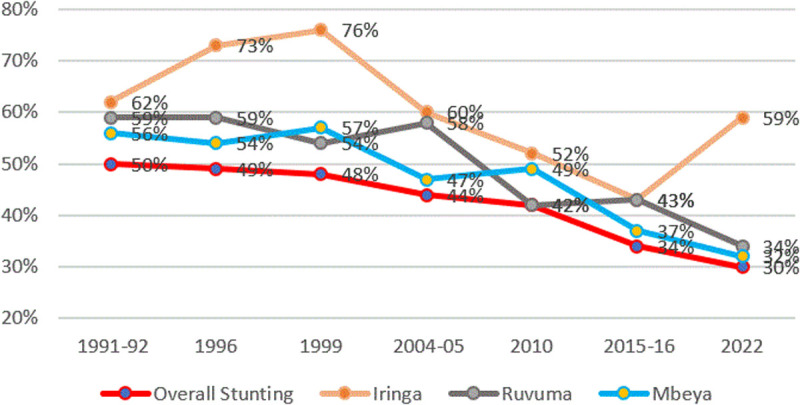
Prevalence of stunting and regions with high stunting rates from TDHS 1991/92–2022. The red line represents the average stunting rates, the orange line represents stunting rates for the Iringa region, the grey line represents stunting rates for the Ruvuma region, and the blue line represents stunting rates for the Mbeya region.

Fig 15, in turn, presents information on trends in stunting in a few selected regions that were performing poorly in 2022 (Iringa, Ruvuma, and Mbeya). Note that all three regions have been performing relatively poorly ever since 1992.

### Bivariate analysis of childhood stunting and related factors in 2022

Table 2 presents bivariate analysis relating 2022 stunting rates to various characteristics associated with the risk of being stunted, including demographic characteristics such as age, place of residence, child gender, zone, mother’s educational status, and wealth index quintile. The South West Highlands and Southern Highlands regions had a statistically significantly higher proportion of stunted children, at 37.8% and 46.2% respectively, compared to Eastern and Zanzibar, which had 22.8% and 17.1% respectively. A higher proportion of stunted children was observed among those aged 18 to 47 months (35.9%) and 9 to 17 months (27.7%). Similarly, males had a higher proportion (33.3%) of stunting compared to females (25.8%). A high proportion (55.6%) of stunting was observed in children with low birthweight. Women with short birth intervals (less than 24 months) had a higher proportion (35.3%) of stunted children compared to those with intervals of 24 to 47 months (29.5%) and those above 47 months (25.6%). Overweight and obese women had a lower proportion of stunted children (24.5% each) compared to those with normal weight (32.9%). Women with secondary education or above had a lower proportion of stunted children (20.3%) compared to those with no formal education (36.3%). Those with more than four ANC visits had a lower proportion of stunted children (26.5%). Women who gave birth before the age of 20 had a higher proportion of stunted children; similarly, those exposed to alcohol had a higher proportion of stunted children (38.3%). Women who used folic acid for more than 90 days had a lower proportion of stunted children (27.8%). Households with limited access to improved water had a higher proportion of stunted children (34.9%); similarly, those lacking access to improved sanitation facilities (37.1%). Households in the lowest SES had the highest proportion of stunted children (38.6%) compared to those in the highest SES (12.6%).

**Table 2:** Bivariate Analysis of Childhood Stunting and Related Factors, TDHS 2022.

	Not Stunted n = 3,423	Stunted n = 1,443	χ2 (df)	P-value
Zones new	n(%)	n(%)		
Lakes	1,197(69.7)	520(30.3)	109.6(8)	<0.001
Northern highlands	369(74.0)	129(25.9)		
Central	363(70.1)	151(29.3)		
Western	328(70.3)	138(29.7)		
South West Highlands	296(62.1)	181(37.9)		
Southern Highlands	136(53.8)	117(46.2)		
Eastern	471(77.2)	139(22.8)		
Southern	143(77.1)	43(22.9)		
Zanzibar	118(82.9)	24(17.1)		
Child age, months				
<9	615(82.7)	129(17.3)	81.5(4)	<0.001
9 - 17	584(72.3)	224(27.7)		
18–47	1,527(64.1)	855(35.9)		
48–59	670(75.2)	221(24.8)		
Child sex				
Male	1,653(66.7)	826(33.3)	18.2(1)	<0.001
Female	1,769(74.2)	617(25.8)		
Child weight				
Low birth weight	82(44.4)	103(55.6)	52.3(2)	<0.001
Normal	1,628(72.4)	619(27.6)		
Child fever				
Yes	376(70.1)	161(29.9)	0.17(1)	0.679
No	3,046(70.4)	1,282(29.6)		
Child Diarrhoea in the 2 weeks before the survey				
Yes	294(69.6)	129(30.5)	0.09(1)	0.755
No	3,129(70.4)	1,314(29.6)		
Birth order				
1	745(68.6)	341(31.4)	16.5(3)	<0.001
2-3	1,347(72.2)	519(27.8)		
4-5	796(73.9)	280(26.0)		
Six and above	535(63.9)	302(36.1)		
Birth interval				
< 24 months	398(64.7)	217(35.3)	14.6(3)	0.002
24 - 47 months	1,322(70.5)	554(29.5)		
Above 47 months	954(74.4)	328(25.6)		
BMI				
Underweight	258(72.9)	96(27.1)	47.6(4)	<0.001
Normal	2,143(67.1)	1,051(32.9)		
Overweight	660(75.5)	214(24.5)		
Obese	350(81.8)	213(24.5)		
Education				
None	667(63.7)	380(36.3)	60.9(2)	<0.001
Primary	1,955(69.5)	859(30.5)		
Secondary and above	800(79.7)	203(20.3)		
ANC visits				
< 4 visits	2,108(68.5)	970(31.5)	13.8(1)	<0.001
>= 4 visits	1,314(73.5)	473(26.5)		
Delivery – caesarean section				
No	3,221(70.5)	1,348(29.5)	2.3(1)	0.129
Yes	202(67.9)	95(32.0)		
Age at birth				
<20 years	1,983(68.1)	929(31.9)	8.7(2)	0.013
20-24	1,137(73.2)	416(26.8)		
25+	302(75.7)	97(24.3)		
Exposure to alcohol				
No	3,286(70.7)	1,358(29.2)	8.3(1)	0.004
Yes	136(61.7)	85(38.3)		
Used folic acid for more than 90 days				
No	2,628(69.8)	1,137(30.2)	3.6(1)	0.058
Yes	794(72.2)	305(27.8)		
Access to improved water				
No	953(65.0)	512(34.9)	34.9(2)	<0.001
Yes	2,350(72.4)	894(27.6)		
Assess to improved sanitation				
Unimproved	1,244(62.9)	734(37.1)	113.4(3)	<0.001
Improved	1,686(77.4)	492(22.6)		
Open defecation	389(67.3)	189(32.8)		
Household head				
Female	805(72.7)	302(27.3)	4.1(1)	0.044
Male	2,618(69.6)	1,140(30.3)		
SES				
Lowest	687(61.5)	430(38.6)	139.7(4)	<0.001
Second	633(61.5)	333(34.5)		
Middle	659(65.5)	295(30.9)		
Fourth	734(72.3)	281(27.7)		
Highest	709(87.4)	102(12.6)		

Note: Regions belonging to the South West Highlands include Katavi, Rukwa, Songwe, and Mbeya, while the Southern Highlands comprise Iringa, Njombe, and Ruvuma.

### Multivariable logistic regression on the characteristics associated with the risk of stunting in 2022

A total of 4,807 observations were included in the regression analysis (Table 3). The table presents the results of a multivariable logistic regression analysis on factors associated with stunting in Tanzania.

**Table 3:** Multivariable logistic regression on the characteristics associated with the risk of stunting, TDHS 2022.

	COR	95%CI	P-value	AOR	95%CI	P-value
Zones new						
Lakes	1					
Northern highlands	0.807	0.602-1.083	0.153	0.916	0.673-1.246	0.576
Central	0.953	0.728-1.247	0.729	0.931	0.696-1.245	0.629
Western	0.975	0.744-1.277	0.853	0.787	0.592-1.045	0.098
South West Highlands	1.407	1.115-1.776	0.004	1.408	1.104-1.794	0.006
Southern Highlands	1.977	1.510-2.589	<0.001	2.368	1.746-3.212	<0.001
Eastern	0.681	0.511-0.905	0.008	0.961	0.702-1.315	0.804
Southern	0.683	0.453-1.030	0.069	0.612	0.392-0.956	0.031
Zanzibar	0.474	0.365-0.616	<0.001	0.894	0.665-1.202	0.459
Child age, months						
<9	1					
9 - 17	1.831	1.356-2.470	<0.001	1.866	1.351-2.578	<0.001
18 - 47	2.678	2.072-3.460	<0.001	3.176	2.381-4.237	<0.001
48 - 59	1.574	1.172-2.113	0.003	2.037	1.416-2.931	<0.001
Child sex						
Female	1					
Male	1.434	1.229-1.673	<0.001	1.584	1.349-1.858	<0.001
Child weight						
Normal	1					
Low birth weight	3.297	2.197-4.947	<0.001	3.639	2.279-5.810	<0.001
Child fever						
No	1					
Yes	1.015	0.776-1.328	0.913			
Child Diarrhoea in the 2 weeks before the survey						
No	1			1		
Yes	1.042	0.790-1.374	0.769	1.215	0.899-1.643	0.204
Birth order						
<2	1					
2-3	0.842	0.684-1.037	0.107	0.747	0.093-5.976	0.783
4-5	0.769	0.608-0.971	0.027	0.577	0.071-4.664	0.606
Six and above	1.233	0.973-1.563	0.083	0.839	0.104-6.771	0.869
Birth interval						
< 24 months	1					
24 - 47 months	0.769	0.605-0.976	0.031	0.762	0.598-0.969	0.027
Above 47 months	0.631	0.489-0.814	<0.001	0.721	0.574-1.000	0.051
BMI						
Normal	1					
Underweight	0.758	0.552-1.041	0.086	0.720	0.520-0.997	0.048
Overweight	0.660	0.537-0.811	<0.001	0.803	0.641-1.005	0.055
Obese	0.453	0.176-0.612	<0.001	0.738	0.529-1.026	0.071
Education						
None	1					
Primary	0.772	0.641-0.931	0.007	0.933	0.757-1.148	0.513
Secondary and above	0.446	0.352-0.566	<0.001	0.715	0.530-0.963	0.027
ANC visits and above						
< 4 visits	1					
>= 4 visits	0.781	0.661-0.923	0.004	0.858	0.694-1.061	0.159
Delivery Caesarean section						
No	0					
Yes	1.127	0.856-1.483	0.395	1.449	1.046-2.007	0.025
Age at birth						
<20 years	1					
20-24	0.782	0.661-0.926	0.004	0.901	0.751-1.081	0.260
25+	0.685	0.522-0.897	0.006	0.997	0.742-1.341	0.988
Exposure to alcohol						
No	1					
Yes	1.394	1.034-1.879	0.029	1.441	1.057-1.963	0.021
Used folic acid for more than 90 days						
No	1					
Yes	0.889	0.733-1.079	0.236			
Access to improved water						
No	1			1		
Yes	0.709	0.602-0.832	<0.001	1.012	0.842-1.217	0.896
Assess to sanitation						
Improved	1			1		
Unimproved	2.019	1.697-2.404	<0.001	1.345	1.055-1.714	0.017
Open defecation	1.662	1.299-2.125	<0.001	1.006	0.723-1.402	0.969
Household head						
Female	1					
Male	1.158	0.961-1.396	0.124			
SES						
Lowest	1					
Second	0.839	0.686-1.026	0.088	0.739	0.590-0.927	0.009
Middle	0.714	0.572-0.891	0.003	0.688	0.524-0.905	0.007
Fourth	0.611	0.477-0.784	<0.001	0.628	0.446-0.883	0.008
Highest	0.230	0.174-0.305	<0.001	0.268	0.178-0.403	<0.001
Observations				4,807		
Wald chi2 (39)				337.6		
Prob > chi2				<0.001		
Pseudo R2				0.098		

Note: Both backward and forward elimination have been used to arrive at the final multivariable model for statistically significant levels, conditional on the p-value being greater than 5 percent (p > 0.05).

Several factors in the multivariable analysis remain significantly associated with stunting even after controlling for confounding factors. These significant variables include households residing in the Southern Highlands (p < 0.001); child age (p < 0.001), child sex (p < 0.001), child birth weight (p < 0.001), birth interval (p < 0.05), mother’s (p < 0.05), mother’s education (p < 0.05), delivery by Caesarean section (p < 0.05), access to sanitation (p < 0.05), and socio-economic status (SES) (p < 0.001).

Factors such as child diarrhoea (AOR = 1.215 [95% CI: 0.899–1.643], p = 0.204), more than four ANC visits (AOR = 0.858 [95% CI: 0.694–1.061], p = 0.159), mother’s age at birth of 20–24 years (AOR = 0.901 [95% CI: 0.750–1.081], p = 0.260), mothers above 25 years of age (AOR = 0.998 [95% CI: 0.742–1.341], p = 0.988), and household access to improved water (AOR = 1.012 [95% CI: 0.842–1.217], p = 0.896) were not significantly related to child stunting.

## Discussion

This study sought to examine trends in stunting among children under five in mainland Tanzania and to identify factors associated with stunting in this population. Despite the persistent high burden of stunting, a notable decline has been observed — from 50% in 1991/2 to 30% in 2022 — highlighting progress amongst ongoing challenges. Several maternal, child, household, and contextual factors remain significantly associated with stunting in 2022.

Maternal factors. Consistent with existing literature, our findings reinforce the importance of birth spacing—specifically, that increased intervals between births significantly reduce the risk of child stunting. Short interpregnancy intervals are linked to adverse outcomes such as preterm birth, low birth weight, and congenital abnormalities [27–29]. Additionally, maternal nutritional status, as indicated by BMI, plays a vital role: higher maternal BMI correlates with lower stunting risk. This aligns with evidence emphasizing the importance of adequate maternal nutrition during preconception and pregnancy for optimal fetal development [30–36], given that maternal undernutrition increases risks such as intrauterine growth restriction [37,38]. For instance, Amaha et al. (2021) demonstrated that each unit increase in maternal BMI reduces the odds of child stunting by approximately 4 percentage points [39]. Similar trends are observed within Tanzania, where children of undernourished mothers (BMI < 18.5) are more likely to be underweight [6]. These findings underscore the impact of maternal nutritional support and care on child growth outcomes.

Globally and within Tanzania, maternal education remains a consistent predictor of child nutritional status. Higher levels of maternal education are associated with reduced stunting prevalence, likely due to improved health literacy, better feeding practices, and increased utilization of healthcare services [40–47]. Mohamed (2023) attributes lower stunting rates among children of educated mothers to their increased awareness of nutrition and participation in growth monitoring programs [48]. Similarly, Moshi (2022) reports that maternal education predicts better child weight outcomes [6]. Education enhances household income and caregivers’ capacity to provide appropriate nutrition, hygiene, and healthcare interventions [42,49].

The 2018 National Antenatal Care Guidelines recommend at least eight ANC visits based on the 2016 WHO model. Our findings indicate that attending four or more ANC visits and delivering in a health facility are associated with decreased rates of severe stunting, aligning with prior research [34–36,39]. For example, a Northern Tanzania study revealed that women attending fewer than four ANC visits had higher risks of delivering low birth weight infants, a key driver of stunting [61].

An intriguing observation is that, over time, children breastfed for more than 12 months consistently exhibit higher stunting prevalence compared to the overall rate, a pattern echoed in other regional studies. Akombi et al. (2017) identified prolonged breastfeeding beyond 12 months as a contributing factor to stunting in sub-Saharan Africa [50], and Cetthakrikul et al. (2020) found similar associations intensified by household economic hardship [51]. The complex relationship between breastfeeding duration and nutritional outcomes warrants further examination, considering the potential confounding effect of socioeconomic factors. One assumption could be that women who breastfeed for a longer period delay or substitute complementary feeding with exclusive breastfeeding exposing children to inadequate nutrient intake.

Child factors. Low birth weight (<2,500 g) remains a strong predictor of stunting, reflecting the lasting effects of intrauterine undernutrition and health insults [40,52]. These infants are more susceptible to respiratory issues, immunodeficiency, and metabolic disorders, which impair growth and development [53]. Gender disparities are also evident, with male children exhibiting higher stunting rates—a pattern that persists across various settings [34–36,39]. Biological differences, higher caloric needs, and greater susceptibility to preterm birth among males may explain this trend [54–57].

Child age is another significant factor; infants under six months tend to have lower stunting rates due to exclusive breastfeeding. However, stunting often becomes prominent after this period, particularly between 24–59 months, associated with inadequate complementary feeding, diarrheal diseases, and intestinal infections—factors that intensify during this developmental window [48,58–62]. Longitudinal data suggest that stunting peaks around 2–3 years and gradually declines afterward, reflecting the cumulative impact of nutrition and infections over time [63].

Household and Environmental Influences. Socioeconomic status is a well-established determinant; households with lower income levels face multiple nutritional and sanitary challenges that predispose children to stunting [64–68]. Even after adjusting for wealth, poor sanitation remains a crucial risk factor. Inadequate sanitation and hygiene practices facilitate infections that impair nutrient absorption and promote growth faltering [67]. Handwashing with soap and access to clean water significantly reduce infection-related growth delays.

Regional Variations and Sociocultural Factors. Regional disparities in stunting within Tanzania are evident and are often linked to socioeconomic and infrastructural factors. Highland areas and regions like Iringa and Njombe face unique challenges, including limited access to clean water, healthcare, and sanitation, compounded by socioeconomic constraints and cultural practices. For example, in Njombe, heavy workloads among women, limited male involvement, alcohol consumption, and poor sanitation contribute markedly to high stunting rates [19–21,69,70]. Dietary monotony—favoring carbohydrate-rich staples like ugali and beans—exacerbates micronutrient deficiencies, further impairing growth.

### Limitations

The study faced several limitations. First and foremost, the cross-sectional nature of the data collection restricts the ability to establish causal relationships between variables. Since the first DHS, there have been changes in methodology, e.g. cut-off points for stunting. To address this, all data were analysed using the new WHO cut-off points for comparison. Additionally, there is a possibility that not all confounders were addressed since no new primary data were collected. A review of the DHS database revealed that the data collection tool does not include information on responsive care, and there are only a limited number of variables related to the safety and security domain. Furthermore, the data is insufficient regarding social care services and the support provided by family and foster care compared to institutional care.

## Conclusion and recommendations

Although child stunting has decreased in the country, it remains higher than the global average of 22.0%. The nation’s stunting rates raise serious concerns about the development and health of children. Stunting is a multifaceted challenge that requires a multi-sectoral approach. It is essential to address region-specific challenges, gender disparities, economic status, and other maternal, child, and household factors. Integrating multisectoral interventions involves coordinating actions across health, agriculture, education, and social protection sectors to tackle factors contributing to stunting, such as poor water, sanitation, and hygiene, socioeconomic inequalities, and gender disparities. Furthermore, current regulations and standards place more emphasis on educating mothers about diet and health than on empowering them economically.

## References

[1] UNICEF. UNICEF/WHO/The World Bank: Joint child malnutrition estimates (JME). Accessed on 21st October 2024. 2023. https://www.who.int/teams/nutrition-and-food-safety/monitoring-nutritional-status-and-food-safety-and-events/joint-child-malnutrition-estimates

[2] FAO, FAO, IFAD, UNICEF, WFP and WHO. The State of Food Security and Nutrition in the World 2022. Repurposing food and agricultural policies to make healthy diets more affordable. Rome, FAO. 2022. 10.4060/cc0639en

[3] UNICEF, WHO, and W. Bank., UNICEF, WHO & World Bank. UNICEF-WHO-World Bank: Joint child malnutrition estimates - Levels and trends (2023 edition). 2023. [Cited 24 April 2023]. https://data.unicef.org/resources/jme-report-2023

[4] Zhu W, Zhu S, Sunguya BF, Huang J. Urban-Rural Disparities in the Magnitude and Determinants of Stunting among Children under Five in Tanzania: Based on Tanzania Demographic and Health Surveys 1991-2016. Int J Environ Res Public Health. 2021;18(10):5184. doi: 10.3390/ijerph18105184 34068222 PMC8153115

[5] Gatica-Domínguez GA, Victora CA, Barros AA. Ethnic inequalities and trends in stunting prevalence among Guatemalan children: an analysis using national health surveys 1995-2014. BMC Public Health. 2014. 10.1186/s12939-019-1016-0 PMC6639956 31319862

[6] Moshi CC, et al. Determinants of underweight among children aged 0–23 months in Tanzania. Food Science & Nutrition. 2022;10(4):1167–74. 35432972 10.1002/fsn3.2748 PMC9007294

[7] Rahayuwati L, Pramukti I, Susanti RD. The Effectiveness of Tele-education for Health Field University Students as a Learning Method during a COVID-19 Pandemic: A Systematic Review. Open Access Maced J Med Sci. 2021;9(T6):159–63. doi: 10.3889/oamjms.2021.7350

[8] Young MF, Nguyen PH, Gonzalez Casanova I, Addo OY, Tran LM, Nguyen S, et al. Role of maternal preconception nutrition on offspring growth and risk of stunting across the first 1000 days in Vietnam: A prospective cohort study. PLoS One. 2018;13(8):e0203201. doi: 10.1371/journal.pone.0203201 30161206 PMC6117029

[9] UNICEF, Malnutrition in mothers soars by 25 per cent in crisis-hit countries, putting women and newborn babies at risk. Accessed on 21st October 2024 2023. https://www.unicef.org/press-releases/malnutrition-mothers-soars-25-cent-crisis-hit-countries-putting-women-and-newborn

[10] Sunguya BF, et al. Trends in prevalence and determinants of stunting in Tanzania: an analysis of Tanzania demographic health surveys (1991-2016). 2019. 10.1186/s12937-019-0505-8 PMC6904996 31823827

[11] URT, National Multisectoral Nutrition Action Plan 2021/22 - 2025/26. 2022. https://www.pmo.go.tz/uploads/documents/sw-1646121553-NMNAP.pdf.

[12] Makoka D, Masibo PK. Is there a threshold level of maternal education sufficient to reduce child undernutrition? Evidence from Malawi, Tanzania and Zimbabwe. (1471-2431 (Electronic)). 10.1186/s12887-015-0406-8 PMC4546212 26297004

[13] Hiliza JN, et al. Prevalence and Factors Associated with Stunting among Public Primary School Pupils in Kasulu District, Western Tanzania. (2520-5285 (Electronic)). 10.24248/eahrj.v4i2.641 PMC8279267 34308235

[14] Musheiguza E, Mahande MJ, Malamala E, Msuya SE, Charles F, Philemon R, et al. Inequalities in stunting among under-five children in Tanzania: decomposing the concentration indexes using demographic health surveys from 2004/5 to 2015/6. Int J Equity Health. 2021;20(1):46. doi: 10.1186/s12939-021-01389-3 33485344 PMC7824937

[15] NBS, Bureau of Statistics [Tanzania] and Macro International Inc. Tanzania Demographic and Health Survey 1992. Calverton, Maryland: Bureau of Statistics and Macro International. 1993. https://www.dhsprogram.com/pubs/pdf/FR45/FR45.pdf

[16] NBS, Bureau of Statistics [Tanzania] and Macro International Inc. 1997. Tanzania Demographic and Health Survey 1996. Calverton, Maryland: Bureau of Statistics and Macro International. 1997. https://www.dhsprogram.com/pubs/pdf/FR83/FR83.pdf

[17] NBS, National Bureau of Statistics [Tanzania] and ORC Macro. Tanzania Demographic and Health Survey 2004-05. Dar es Salaam, Tanzania. 2005. https://dhsprogram.com/publications/publication-fr173-dhs-final-reports.cfm

[18] NBS, National Bureau of Statistics [Tanzania] and ICF Macro. 2011. Tanzania Demographic and Health Survey 2010. Dar es Salaam, Tanzania: NBS and ICF Macro. 2010. https://dhsprogram.com/pubs/pdf/FR243/FR243%5B24June2011%5D.pdf

[19] MoHCDGEC, Ministry of Health Community Development, Gender, Elderly and Children - MoHCDGEC/Tanzania Mainland, Ministry of Health - MoH/Zanzibar, National Bureau of Statistics - NBS/Tanzania, et al. Tanzania demographic and health survey and malaria indicator survey 2015-16. Dar es Salaam: MoHCDGEC, MoH, NBS, OCGS, and ICF; 2016 https://dhsprogram.com/pubs/pdf/fr321/fr321.pdf

[20] Ministry of Health (MoH) TM, Ministry of Health (MoH) Z, National Bureau of Statistics (NBS), Office of the Chief Government Statistician (OCGS), ICF. Tanzania Demographic and Health Survey and Malaria Indicator Survey 2022 Key Indicators Report. Dodoma, Tanzania, and Rockville, Maryland, USA: MoH, NBS, OCGS, and ICF. 2023. https://dhsprogram.com/pubs/pdf/PR144/PPR144.pdf

[21] NBS NB of S [Tanzania], Macro International Inc. Tanzania Reproductive and Child Health Survey 1999. Calverton, Maryland: National Bureau of Statistics and Macro International Inc. https://dhsprogram.com/publications/publication-fr112-dhs-final-reports.cfm

[22] WHO Multicentre Growth Reference Study Group. WHO Child Growth Standards based on length/height, weight and age. Acta Paediatr Suppl. 2006;450:76–85. doi: 10.1111/j.1651-2227.2006.tb02378.x 16817681

[23] WHO WHO. Global nutrition targets 2025: stunting policy brief. Geneva: World Health Organization. 2014.

[24] Sinha RK, et al. Determinants of stunting, wasting, and underweight in five high-burden pockets of four Indian states. Indian Pediatr. 10.4103/ijcm.IJCM_151_18 PMC6319291 30662180

[25] WHO, Physical status: the use and interpretation of anthropometry. Report of a WHO Expert Committee. 2006(0512-3054 (Print)). 8594834

[26] NBS, Ministry of Health (MoH) [Tanzania Mainland], Ministry of Health (MoH) [Zanzibar], National Bureau of Statistics (NBS), Office of the Chief Government Statistician (OCGS), and ICF. Tanzania Demographic and Health Survey and Malaria Indicator Survey 2022 Key Indicators Report. Dodoma, Tanzania, and Rockville, Maryland, USA: MoH, NBS, OCGS, and ICF. 2022. 2023.

[27] Dhamrait G, Fletcher T, Foo D, Taylor CL, Pereira G. The effects of birth spacing on early childhood development in high-income nations: A systematic review. Front Pediatr. 2022;10:851700. doi: 10.3389/fped.2022.851700 36507145 PMC9732574

[28] Dhamrait GK, Taylor CL, Pereira G. Interpregnancy intervals and child development at age 5: a population data linkage study. BMJ Open. 2021;11(3):e045319. doi: 10.1136/bmjopen-2020-045319 33757954 PMC7993213

[29] Wang Y, Zeng C, Chen Y, Yang L, Tian D, Liu X, et al. Short interpregnancy interval can lead to adverse pregnancy outcomes: A meta-analysis. Front Med (Lausanne). 2022;9:922053. doi: 10.3389/fmed.2022.922053 36530890 PMC9747778

[30] Milner EM, Fiorella KJ, Mattah BJ, Bukusi E, Fernald LCH. Timing, intensity, and duration of household food insecurity are associated with early childhood development in Kenya. Matern Child Nutr. 2018;14(2):e12543. doi: 10.1111/mcn.12543 29063732 PMC6866123

[31] Naaz A, Muneshwar KN. How Maternal Nutritional and Mental Health Affects Child Health During Pregnancy: A Narrative Review. Cureus. 2023;15(11):e48763. doi: 10.7759/cureus.48763 38098932 PMC10719542

[32] Gogos A, Thomson S, Drummond K, Holland L, O’Hely M, Dawson S, et al. Socioeconomic adversity, maternal nutrition, and the prenatal programming of offspring cognition and language at two years of age through maternal inflammation. Brain Behav Immun. 2024;122:471–82. doi: 10.1016/j.bbi.2024.08.033 39163911

[33] Sinha RK, Dua R, Bijalwan V, Rohatgi S, Kumar P. Determinants of Stunting, Wasting, and Underweight in Five High-Burden Pockets of Four Indian States. Indian J Community Med. 2018;43(4):279–83. doi: 10.4103/ijcm.IJCM_151_18 30662180 PMC6319291

[34] Das S, Chanani S, Shah More N, Osrin D, Pantvaidya S, Jayaraman A. Determinants of stunting among children under 2 years in urban informal settlements in Mumbai, India: evidence from a household census. J Health Popul Nutr. 2020;39(1):10. doi: 10.1186/s41043-020-00222-x 33246506 PMC7693500

[35] Mtongwa RH, Festo C, Elisaria E. A comparative analysis of determinants of low birth weight and stunting among under five children of adolescent and non-adolescent mothers using 2015/16 Tanzania Demographic and Health Survey (TDHS). BMC Nutr. 2021;7(1):64. doi: 10.1186/s40795-021-00468-6 34732260 PMC8567641

[36] Islam MS, Zafar Ullah AN, Mainali S, Imam MA, Hasan MI. Determinants of stunting during the first 1,000 days of life in Bangladesh: A review. Food Sci Nutr. 2020;8(9):4685–95. doi: 10.1002/fsn3.1795 32994930 PMC7500796

[37] Haque R, Alam K, Rahman SM, Mustafa MUR, Ahammed B, Ahmad K, et al. Nexus between maternal underweight and child anthropometric status in South and South-East Asian countries. Nutrition. 2022;98:111628. doi: 10.1016/j.nut.2022.111628 35436692

[38] Woldeamanuel GG, Geta TG, Mohammed TP, Shuba MB, Bafa TA. Effect of nutritional status of pregnant women on birth weight of newborns at Butajira Referral Hospital, Butajira, Ethiopia. SAGE Open Med. 2019;7:2050312119827096. doi: 10.1177/2050312119827096 30728970 PMC6351719

[39] Amaha ND, Woldeamanuel BT. Maternal factors associated with moderate and severe stunting in Ethiopian children: analysis of some environmental factors based on 2016 demographic health survey. Nutr J. 2021;20(1):18. doi: 10.1186/s12937-021-00677-6 33639943 PMC7916293

[40] Mulyaningsih T, Mohanty I, Widyaningsih V, Gebremedhin TA, Miranti R, Wiyono VH. Beyond personal factors: Multilevel determinants of childhood stunting in Indonesia. PLoS One. 2021;16(11):e0260265. doi: 10.1371/journal.pone.0260265 34797892 PMC8604318

[41] Elmighrabi NF, Fleming CAK, Agho KE. Factors Associated with Childhood Stunting in Four North African Countries: Evidence from Multiple Indicator Cluster Surveys, 2014-2019. Nutrients. 2024;16(4):473. doi: 10.3390/nu16040473 38398798 PMC10892369

[42] Semba RD, de Pee S, Sun K, Sari M, Akhter N, Bloem MW. Effect of parental formal education on risk of child stunting in Indonesia and Bangladesh: a cross-sectional study. Lancet. 2008;371(9609):322–8. doi: 10.1016/S0140-6736(08)60169-5 18294999

[43] Balla S, Goli S, Vedantam S, Rammohan A. Progress in child stunting across the world from 1990 to 2015: testing the global convergence hypothesis. Public Health Nutr. 2021;24(17):5598–607. doi: 10.1017/S136898002100375X 34462036 PMC10195413

[44] Ertem IO, Atay G, Dogan DG, Bayhan A, Bingoler BE, Gok CG, et al. Mothers’ knowledge of young child development in a developing country. Child Care Health Dev. 2007;33(6):728–37. doi: 10.1111/j.1365-2214.2007.00751.x 17944782

[45] Hasan MN, Babu MR, Chowdhury MAB, Rahman MM, Hasan N, Kabir R, et al. Early childhood developmental status and its associated factors in Bangladesh: a comparison of two consecutive nationally representative surveys. BMC Public Health. 2023;23(1):687. doi: 10.1186/s12889-023-15617-8 37046226 PMC10099688

[46] Gizaw B, Gebremedhin S. Factors associated with low birthweight in North Shewa zone, Central Ethiopia: case-control study. Ital J Pediatr. 2018;44(1):76. doi: 10.1186/s13052-018-0516-7 29973240 PMC6030760

[47] Casale D, Espi G, Norris SA. Estimating the pathways through which maternal education affects stunting: evidence from an urban cohort in South Africa. Public Health Nutr. 2018;21(10):1810–8. doi: 10.1017/S1368980018000125 29455701 PMC10260984

[48] Mohamed TM, Nyaruhucha CN. Household and Community Factors Affecting Nutritional Status of Under-five Children (6-59 months) in Gairo District Using Composite Index of Anthropometric Failure. Tanz J Sci. 2023;49(1):76–85. doi: 10.4314/tjs.v49i1.7

[49] Stryzhak O. The relationship between education, income, economic freedom and happiness. 2020. https://ouci.dntb.gov.ua/en/works/4bvj8YL4/

[50] Akombi BJ, Agho KE, Hall JJ, Wali N, Renzaho AMN, Merom D. Stunting, Wasting and Underweight in Sub-Saharan Africa: A Systematic Review. Int J Environ Res Public Health. 2017;14(8):863. doi: 10.3390/ijerph14080863 28788108 PMC5580567

[51] Cetthakrikul N, Topothai C, Suphanchaimat R, Tisayaticom K, Limwattananon S, Tangcharoensathien V. Childhood stunting in Thailand: when prolonged breastfeeding interacts with household poverty. BMC Pediatr. 2018;18(1):395. doi: 10.1186/s12887-018-1375-5 30591029 PMC6309093

[52] Tiwari R, Ausman LM, Agho KE. Determinants of stunting and severe stunting among under-fives: evidence from the 2011 Nepal Demographic and Health Survey. BMC Pediatr. 2014;14:239. doi: 10.1186/1471-2431-14-239 25262003 PMC4263111

[53] Jana A, Dey D, Ghosh R. Contribution of low birth weight to childhood undernutrition in India: evidence from the national family health survey 2019-2021. BMC Public Health. 2023;23(1):1336. doi: 10.1186/s12889-023-16160-2 37438769 PMC10337105

[54] Samuel A, Osendarp SJM, Feskens EJM, Lelisa A, Adish A, Kebede A, Brouwer ID. Gender differences in nutritional status and determinants among infants (6-11 m): a cross-sectional study in two regions in Ethiopia. BMC Public Health. 2022 Feb 26;22(1):401. doi: 10.1186/s12889-022-12772-2 ; PMCID: PMC8881837. 35219315 PMC8881837

[55] Thompson AL. Greater male vulnerability to stunting? Evaluating sex differences in growth, pathways and biocultural mechanisms. Ann Hum Biol. 2021;48(6):466–73. doi: 10.1080/03014460.2021.1998622 35105202 PMC9205267

[56] Pongou R. Why is infant mortality higher in boys than in girls? A new hypothesis based on preconception environment and evidence from a large sample of twins. Demography. 2013;50(2):421–44. doi: 10.1007/s13524-012-0161-5 23151996

[57] Galante L, Milan AM, Reynolds CM, Cameron-Smith D, Vickers MH, Pundir S. Sex-Specific Human Milk Composition: The Role of Infant Sex in Determining Early Life Nutrition. Nutrients. 2018;10(9):1194. doi: 10.3390/nu10091194 30200404 PMC6165076

[58] Ricci JA, Becker S. Risk factors for wasting and stunting among children in Metro Cebu, Philippines. Am J Clin Nutr. 1996;63(6):966–75. doi: 10.1093/ajcn/63.6.966 8644694

[59] Roth DE, Krishna A, Leung M, Shi J, Bassani DG, Barros AJD. Early childhood linear growth faltering in low-income and middle-income countries as a whole-population condition: analysis of 179 Demographic and Health Surveys from 64 countries (1993-2015). Lancet Glob Health. 2017;5(12):e1249–57. doi: 10.1016/S2214-109X(17)30418-7 29132614 PMC5695758

[60] Karlsson O, Kim R, Moloney GM, Hasman A, Subramanian SV. Patterns in child stunting by age: A cross-sectional study of 94 low- and middle-income countries. Matern Child Nutr. 2023;19(4):e13537. doi: 10.1111/mcn.13537 37276243 PMC10483943

[61] Mvunta MH, Mboya IB, Msuya SE, John B, Obure J, Mahande MJ. Incidence and recurrence risk of low birth weight in Northern Tanzania: A registry based study. PLoS One. 2019;14(4):e0215768. doi: 10.1371/journal.pone.0215768 31009497 PMC6476513

[62] Bommer C, Vollmer S, Subramanian SV. How socioeconomic status moderates the stunting-age relationship in low-income and middle-income countries. BMJ Glob Health. 2019;4(1):e001175. doi: 10.1136/bmjgh-2018-001175 30899561 PMC6407538

[63] Wake SK, Zewotir T, Lulu K, Fissuh YH. Longitudinal trends and determinants of stunting among children aged 1-15 years. Arch Public Health. 2023;81(1):60. doi: 10.1186/s13690-023-01090-7 37081559 PMC10116743

[64] Ribe IG, Svensen E, Lyngmo BA, Mduma E, Hinderaker SG. Determinants of early child development in rural Tanzania. Child Adolesc Psychiatry Ment Health. 2018;12:18. doi: 10.1186/s13034-018-0224-5 29568326 PMC5859781

[65] Nshimyiryo A, Hedt-Gauthier B, Mutaganzwa C, Kirk CM, Beck K, Ndayisaba A, et al. Risk factors for stunting among children under five years: a cross-sectional population-based study in Rwanda using the 2015 Demographic and Health Survey. BMC Public Health. 2019;19(1):175. doi: 10.1186/s12889-019-6504-z 30744614 PMC6371425

[66] Muche A, Dewau R. Severe stunting and its associated factors among children aged 6-59 months in Ethiopia; multilevel ordinal logistic regression model. Ital J Pediatr. 2021;47(1):161. doi: 10.1186/s13052-021-01110-8 34311750 PMC8314542

[67] Habimana J de D, Uwase A, Korukire N, Jewett S, Umugwaneza M, Rugema L, et al. Prevalence and Correlates of Stunting among Children Aged 6-23 Months from Poor Households in Rwanda. Int J Environ Res Public Health. 2023;20(5):4068. doi: 10.3390/ijerph20054068 36901076 PMC10001740

[68] Shinsugi C, Matsumura M, Karama M, Tanaka J, Changoma M, Kaneko S. Factors associated with stunting among children according to the level of food insecurity in the household: a cross-sectional study in a rural community of Southeastern Kenya. BMC Public Health. 2015;15:441. doi: 10.1186/s12889-015-1802-6 25924925 PMC4428099

[69] Elisaria E, Mrema J, Festo C. Improving maternal and adolescent nutrition in Tanzania. Situational Analysis. Dar es Salaam, Tanzania. 2020. Cited 0th October 2024 https://www.tfnc.go.tz/uploads/publications/sw1700578633-IMAN_Barrier%20Analysis%20Report.pdf

[70] Malima W. Experts outline factors behind stunting in Njombe. 2024. Cited 10th October 2024. https://dailynews.co.tz/experts-outline-factors-behind-stunting-in-njombe/

