# Supplementary material for: Thirty years of declining stunting in Tanzania: Trends and ongoing challenges
Source: PLoS One. 2025 Jul 29;20(7):e0327779. doi: 10.1371/journal.pone.0327779 (PMC12306788; doi:10.1371/journal.pone.0327779)
Supplement: Attachment — Submitted filename: PONE-D-24-53994.pdf [file pone.0327779.s002.pdf]

PLOS ONE

# Thirty Years of Stunting in Tanzania

--Manuscript Draft--

| | |
|---|---|
| **Manuscript Number:** | PONE-D-24-53994 |
| **Article Type:** | Research Article |
| **Full Title:** | Thirty Years of Stunting in Tanzania |
| **Short Title:** | Thirty Years of Stunting in Tanzania |
| **Corresponding Author:** | August Kuwawenaruwa, MA Economics<br>Ifakara Health Institute<br>Dar es Salaam, TANZANIA, UNITED REPUBLIC OF |
| **Keywords:** | Child growth; Nutritional; Stunting; Demographic Health Survey; Tanzania |
| **Abstract:** | Background<br><br>Tanzania has made considerable gains in children's nutrition between 1991/92 and 2022. The country's stunting rate has decreased from 50% in 1992 to 30% in 2022. However, stunting varies greatly among regions. The purpose of this study was to examine regional trends in stunting rates and associated characteristics related to the risk of stunting among children under the age of five in mainland Tanzania from 1991/1992 to 2022.<br><br>Methods<br><br>Descriptive statistics were calculated using Tanzania Demographic and Health Survey (TDHS) data to examine stunting trends and characteristics associated with stunting in the country. Frequencies and percentages were calculated and presented in tables and graphs as cross-sectional data points. The descriptive statistics led the selection of covariates for the multilevel binary logistic regression model.<br><br>Results<br><br>Over the previous three decades, stunting rates in under-five boys have consistently been 4-7 percentage points (ppt) greater than those in girls of the same age. The frequency of stunted children was higher in households with the lowest socioeconomic status (SES) (38.6%) than in those with the highest SES status (12.6%). Similar to the trend analysis, a multilevel regression analysis found that residency in the South-Western Highlands (AOR = 1.407; 95% CI: 1.114-1.776, p < 0.005) and the Southern Highlands (AOR = 1.977; 95% CI: 1.510-2.589, p < 0.001) was significant associated with risk of being stunting. Other characteristics associated with risk of being stunted included male children (AOR = 1.543 [95% CI: 1.315 - 1.811], p < 0.001), low birth weight (AOR = 3.616 [95% CI: 2.284 - 5.727], p < 0.001), birth interval of 24 to 47 months (AOR = 0.754 [95% CI: 0.592 - 0.959], p < 0.001), secondary education and above (AOR = 0.731 [95% CI: 0.542 – 0.985], p < 0.005), maternal exposure to alcohol (AOR = 1.566 [95% CI: 1.092 – 2.246], p < 0.005), unimproved sanitation facilities (AOR = 1.341 [95% CI: 1.052 – 1.709], p < 0.005) as well as higher socioeconomic status (AOR = 0.269 [95% CI: 0.179 – 0.404], p < 0.001).<br><br>Conclusion<br><br>The nation's stunting tendencies raise serious concerns about the development and health of children. These discrepancies stem from factors at the mother, family, community, and child levels. There is a disparity in regional stunting prevalence, with certain areas consistently reporting high stunting rates. Nutrition focal points should be established within regions and councils to ensure that all sectors effectively address the decentralisation of nutrition actions and resources. To combat stunting, eliminate structural obstacles including empower marginalised groups, a multisectoral strategy is required. Furthermore, current regulations and standards place more emphasis on educating mothers about diet and health than they do on empowering them economically. |

Powered by Editorial Manager® and ProduXion Manager® from Aries Systems Corporation

| | |
|---|---|
| **Order of Authors:** | Ester Elisaria |
| | Laura van der Erve |
| | Bet Caeyers |
| | Esther Nkuba |
| | August Kuwawenaruwa, MA Economics |
| **Opposed Reviewers:** | |
| **Additional Information:** | |
| **Question** | **Response** |
| **Financial Disclosure**<br><br>Enter a financial disclosure statement that describes the sources of funding for the work included in this submission. Review the submission guidelines for detailed requirements. View published research articles from *PLOS ONE* for specific examples.<br><br>This statement is required for submission and **will appear in the published article** if the submission is accepted. Please make sure it is accurate.<br><br>**Funded studies**<br>Enter a statement with the following details:<br>• Initials of the authors who received each award<br>• Grant numbers awarded to each author<br>• The full name of each funder<br>• URL of each funder website<br>• Did the sponsors or funders play any role in the study design, data collection and analysis, decision to publish, or preparation of the manuscript?<br><br>Did you receive funding for this work? | Yes |
| Please add funding details.<br>as follow-up to "**Financial Disclosure**<br><br>Enter a financial disclosure statement that describes the sources of funding for the work included in this submission. Review the submission guidelines for detailed requirements. View published research articles from *PLOS ONE* for specific examples. | This project was funded with UK International Development from the UK government and New Zealand's Ministry of Foreign Affairs and Trade (MFAT) contract PO number 10087; however, the views expressed do not necessarily reflect the UK or New Zealand's government official policies |

Powered by Editorial Manager® and ProduXion Manager® from Aries Systems Corporation

| | |
|---|---|
| This statement is required for submission and **will appear in the published article** if the submission is accepted. Please make sure it is accurate.<br><br>**Funded studies**<br>Enter a statement with the following details:<br>• Initials of the authors who received each award<br>• Grant numbers awarded to each author<br>• The full name of each funder<br>• URL of each funder website<br>• Did the sponsors or funders play any role in the study design, data collection and analysis, decision to publish, or preparation of the manuscript?<br><br>Did you receive funding for this work?" | |
| Please select the country of your main research funder (please select carefully as in some cases this is used in fee calculation).<br>as follow-up to "**Financial Disclosure**<br><br>Enter a financial disclosure statement that describes the sources of funding for the work included in this submission. Review the submission guidelines for detailed requirements. View published research articles from *PLOS ONE* for specific examples.<br><br>This statement is required for submission and **will appear in the published article** if the submission is accepted. Please make sure it is accurate. | TANZANIA, UNITED REPUBLIC OF - TZ |

| | |
|---|---|
| **Funded studies**<br>Enter a statement with the following details:<br>• Initials of the authors who received each award<br>• Grant numbers awarded to each author<br>• The full name of each funder<br>• URL of each funder website<br>• Did the sponsors or funders play any role in the study design, data collection and analysis, decision to publish, or preparation of the manuscript?<br><br>Did you receive funding for this work?" | |
| **Competing Interests**<br><br>Use the instructions below to enter a competing interest statement for this submission. On behalf of all authors, disclose any competing interests that could be perceived to bias this work—acknowledging all financial support and any other relevant financial or non-financial competing interests.<br><br>This statement is required for submission and **will appear in the published article** if the submission is accepted. Please make sure it is accurate and that any funding sources listed in your Funding Information later in the submission form are also declared in your Financial Disclosure statement.<br><br>View published research articles from *PLOS ONE* for specific examples. | The authors have declared that no competing interests exist. |

Powered by Editorial Manager® and ProduXion Manager® from Aries Systems Corporation

<table>
<tr>
<td>

**NO authors have competing interests**

Enter: *The authors have declared that no competing interests exist.*

**Authors with competing interests**

Enter competing interest details beginning with this statement:

*I have read the journal's policy and the authors of this manuscript have the following competing interests: [insert competing interests here]*

* typeset

</td>
<td></td>
</tr>
<tr>
<td>

**Ethics Statement**

Enter an ethics statement for this submission. This statement is required if the study involved:

- Human participants
- Human specimens or tissue
- Vertebrate animals or cephalopods
- Vertebrate embryos or tissues
- Field research

Write "N/A" if the submission does not require an ethics statement.

General guidance is provided below. Consult the submission guidelines for detailed instructions. **Make sure that all information entered here is included in the Methods section of the manuscript.**

</td>
<td>

This is a secondary analysis of the Tanzania Demographic Health Surveys collected by the government through the National Bureau of Statistics. Prior to data collection, the Institutional Review Board (IRB) and Tanzania's National Medical Research Committee approved the study protocol and DHS survey questionnaires. All participants provided written informed consent before the interviews. For illiterate individuals, a written consent form was presented orally in the presence of a legally permitted representative or witness, such as a friend, relative, or someone not affiliated with the research team. Parental or guardian consent was obtained for children and adolescents participating in the study. Every qualified respondent was questioned in the most private setting possible, without the presence of a third party.

</td>
</tr>
</table>

*Powered by Editorial Manager® and ProduXion Manager® from Aries Systems Corporation*

| | |
|---|---|
| **Format for specific study types**<br><br>**Human Subject Research (involving human participants and/or tissue)**<br>• Give the name of the institutional review board or ethics committee that approved the study<br>• Include the approval number and/or a statement indicating approval of this research<br>• Indicate the form of consent obtained (written/oral) or the reason that consent was not obtained (e.g. the data were analyzed anonymously)<br><br>**Animal Research (involving vertebrate animals, embryos or tissues)**<br>• Provide the name of the Institutional Animal Care and Use Committee (IACUC) or other relevant ethics board that reviewed the study protocol, and indicate whether they approved this research or granted a formal waiver of ethical approval<br>• Include an approval number if one was obtained<br>• If the study involved *non-human primates*, add *additional details* about animal welfare and steps taken to ameliorate suffering<br>• If anesthesia, euthanasia, or any kind of animal sacrifice is part of the study, include briefly which substances and/or methods were applied<br><br>**Field Research**<br><br>Include the following details if this study involves the collection of plant, animal, or other materials from a natural setting:<br>• Field permit number<br>• Name of the institution or relevant body that granted permission | |
| **Data Availability**<br><br>Authors are required to make all data underlying the findings described fully available, without restriction, and from the time of publication. PLOS allows rare exceptions to address legal and ethical concerns. See the PLOS Data Policy and FAQ for detailed information. | Yes - all data are fully available without restriction |

*Powered by Editorial Manager® and ProduXion Manager® from Aries Systems Corporation*

| | |
|---|---|
| A Data Availability Statement describing where the data can be found is required at submission. Your answers to this question constitute the Data Availability Statement and **will be published in the article**, if accepted.<br><br>**Important:** Stating 'data available on request from the author' is not sufficient. If your data are only available upon request, select 'No' for the first question and explain your exceptional situation in the text box.<br><br>Do the authors confirm that all data underlying the findings described in their manuscript are fully available without restriction? | |
| **Describe where the data may be found in full sentences. If you are copying our sample text, replace any instances of XXX with the appropriate details.**<br><br>• If the data are **held or will be held in a public repository**, include URLs, accession numbers or DOIs. If this information will only be available after acceptance, indicate this by ticking the box below. For example: *All XXX files are available from the XXX database (accession number(s) XXX, XXX.)*.<br>• If the data are all contained **within the manuscript and/or Supporting Information files**, enter the following: *All relevant data are within the manuscript and its Supporting Information files.*<br>• If neither of these applies but you are able to provide **details of access elsewhere**, with or without limitations, please do so. For example:<br><br>*Data cannot be shared publicly because of [XXX]. Data are available from the XXX Institutional Data Access / Ethics Committee (contact via XXX) for researchers who meet the criteria for access to confidential data.*<br><br>*The data underlying the results presented in the study are available from (include the name of the third party* | The dataset(s) supporting the conclusions of this article is owned by the Demographic and Health Surveys (DHS) Program, ICF, 530 Gaither Road, Suite 500, Rockville, MD 20850, USA and available upon request. |

Powered by Editorial Manager® and ProduXion Manager® from Aries Systems Corporation

| | |
|---|---|
| *and contact information or URL).*<br>• This text is appropriate if the data are owned by a third party and authors do not have permission to share the data.<br><br>* typeset | |
| Additional data availability information: | |

Powered by Editorial Manager® and ProduXion Manager® from Aries Systems Corporation

Ifakara Health Institute

PO Box 78373, Tanzania

24th November 2024

To the Editor,

PLOS ONE

**Thirty Years of Stunting in Tanzania**

We are delighted to submit the manuscript titled "***Thirty Years of Stunting in Tanzania***" to PLOS ONE.

Tanzania has made considerable gains in children's nutrition between 1991/92 and 2022. The country's stunting rate has decreased from 50% in 1992 to 30% in 2022. However, stunting varies greatly among regions. The purpose of this study was to examine regional trends in stunting rates and associated characteristics related to the risk of stunting among children under the age of five in Tanzania from 1991/1992 to 2022. Over the previous three decades, stunting rates in under-five boys have consistently been 4-7 percentage points (ppt) greater than those in girls of the same age. The frequency of stunted children was higher in households with the lowest socioeconomic status (SES) (38.6%) than in those with the highest SES status (12.6%). We believe the results of this study will be of interest to the audience of PLOS ONE.

We hope this manuscript will be of interest to your journal.

We look forward to your response.

Yours sincerely,

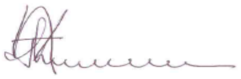

August Kuwawenaruwa

Manuscript

Click here to access/download;Manuscript;Stunting Tanzania 15.11.2024.docx

# Thirty Years of Stunting in Tanzania

**Authors**: Ester Elisaria[1], Laura van der Erve[2], Bet Caeyers[3,4], Esther Nkuba[5], August Kuwawenaruwa[1*]

* Corresponding Author

**Author Affiliations**

1. Ifakara Health Institute, Plot 463, Kiko Avenue Mikocheni P.O. Box 78 373, Dar es Salaam, Tanzania. Telephone: +255 222 774 714, Fax: +255 222 771 714.
2. Oxford Policy Management, 40-41 Park End Street, Oxford, OX1 1JD, United Kingdom.
3. Chr. Michelsen Institute (CMI), P.O.Box 6033. N-5892 Bergen, Norway.
4. Centre for Experimental Research on Fairness, Inequality and Rationality (FAIR) & Norwegian School of Economics.
5. Tanzania Food and Nutrition Center (TFNC), 22 Barack Obama Avenue, P.O Box 977, Dar-es-Salaam Tanzania.

Email: Ester Elisaria (EE) **eelisaria@ihi.or.tz**; Laura van der Erve (LE) **laura.vandererve@opml.co.uk** ; Bet Caeyers (BC) **Bet.Caeyers@cmi.no;** Esther Nkuba (EN) **esther.nkuba@tfnc.go.tz;** August Kuwawenaruwa (AK) **ajoachim@ihi.or.tz**

## Abstract

### Background

Tanzania has made considerable gains in children's nutrition between 1991/92 and 2022. The country's stunting rate has decreased from 50% in 1992 to 30% in 2022. However, stunting varies greatly among regions. The purpose of this study was to examine regional trends in stunting rates and associated characteristics related to the risk of stunting among children under the age of five in mainland Tanzania from 1991/1992 to 2022.

### Methods

Descriptive statistics were calculated using Tanzania Demographic and Health Survey (TDHS) data to examine stunting trends and characteristics associated with stunting in the country. Frequencies and percentages were calculated and presented in tables and graphs as cross-sectional data points. The descriptive statistics led the selection of covariates for the multilevel binary logistic regression model.

### Results

Over the previous three decades, stunting rates in under-five boys have consistently been 4-7 percentage points (ppt) greater than those in girls of the same age. The frequency of stunted children was higher in households with the lowest socioeconomic status (SES) (38.6%) than in those with the highest SES status (12.6%). Similar to the trend analysis, a multilevel regression analysis found that residency in the South-Western Highlands (AOR = 1.407; 95% CI: 1.114-1.776, $p < 0.005$) and the Southern Highlands (AOR = 1.977; 95% CI: 1.510-2.589, $p < 0.001$) was significant associated with risk of being stunting. Other characteristics associated with risk of being stunted included male children (AOR = 1.543 [95% CI: 1.315 - 1.811], $p < 0.001$), low birth weight (AOR = 3.616 [95% CI: 2.284 - 5.727], $p < 0.001$), birth interval of 24 to 47 months (AOR = 0.754 [95% CI: 0.592 - 0.959], $p < 0.001$), secondary education and above (AOR = 0.731 [95% CI: 0.542 – 0.985], $p < 0.005$), maternal exposure to alcohol (AOR = 1.566 [95% CI: 1.092 – 2.246], $p < 0.005$), unimproved

sanitation facilities (AOR = 1.341 [95% CI: 1.052 – 1.709], $p < 0.005$) as well as higher socioeconomic status (AOR = 0.269 [95% CI: 0.179 – 0.404], $p < 0.001$).

**Conclusion**

The nation's stunting tendencies raise serious concerns about the development and health of children. These discrepancies stem from factors at the mother, family, community, and child levels. There is a disparity in regional stunting prevalence, with certain areas consistently reporting high stunting rates. Nutrition focal points should be established within regions and councils to ensure that all sectors effectively address the decentralisation of nutrition actions and resources. To combat stunting, eliminate structural obstacles including empower marginalised groups, a multisectoral strategy is required. Furthermore, current regulations and standards place more emphasis on educating mothers about diet and health than they do on empowering them economically.

**Keywords**: Child growth; Nutritional; Stunting; Demographic Health Survey; Tanzania.

## Introduction

In 2022, globally an estimated 148.1 million children under the age of five were classified as stunted, indicating that they were too short for their age [1]. The prevalence of stunting was notably higher among children residing in low- and lower-middle-income countries, which accounted for 89% of the global burden of stunting in 2020. Additionally, these children were more likely to come from rural areas and have mothers who had not received an education. Alarmingly, nearly one-third of countries in Northern Africa, Oceania, and the Caribbean are experiencing an increase in the number of stunted children [2]. This trend underscores a concerning lack of progress towards the goal of halving the prevalence of stunting by the year 2030. Central Africa is the most severely affected subregion, with a prevalence rate of 37.4% [3]. In the case of Tanzania, the Demographic Health Surveys (DHS) data collected from 1991/92-2022 indicates the country had a decrease in stunting from 50% in 1991/92 to 30% [2], with a significant disparity between rural and urban areas [4]. The World Bank and UNICEF

agree that heightened efforts needed to reach the global goal of reducing the number of children suffering from stunting to 89 million by 2030 [1].

Earlier research indicated that the disparity in stunting prevalence between rural and urban areas is linked to several factors, including access to public services, education, and economic resources [4-6]. The findings further demonstrated that understanding the underlying causes of stunting in different contexts can facilitate the development of tailored interventions for both urban and rural settings [4]. Stunting occurs due to inadequate nutrition during pre-conception, pregnancy and early childhood [7-10]. Children affected by stunting may never reach their full potential height and may not fully develop their cognitive abilities. These children start life at a significant disadvantage, and the consequences persist into adulthood. They encounter challenges in learning at school, have lower earning potential as adults, and face obstacles to engaging in their communities [3]. Most Tanzanians have undiversified diets, where an average of 71% of all energy comes from staple foods. Even in the wealthiest segment of the population, nearly 60% of energy is derived solely from staple foods [11]. The 2022 TDHS indicates that only 64% of children aged less than six months were exclusively breastfed, 19% of children aged 6-23 months, and 25% of women aged 15-49 years received a diversified diet. In addition, 11% of children aged 6–59 months were given iron-containing supplements in the last 12 months at the health facility, and 53% were given vitamin A supplements in the last 6 months.

To the best of our knowledge, none of the previous studies conducted in Tanzania [4, 6, 10, 12-14] has thoroughly examined the time trends of stunting alongside child-related factors (such as birthweight, gender, and age), maternal factors (including breastfeeding practices, maternal education, nutritional status, and the number of antenatal care visits), household-related factors (such as hygiene practices, socio-economic status, the gender of the household head, and child infections, particularly diarrhoea) and cross-regional comparisons from 1991/92 to 2022. Therefore, the primary aim of this study was to examine the trends of stunting and characteristics associated with risk of being stunted in Tanzania.

## Materials and Methods

### Data Sources

The DHS are nationally representative, repeated cross-sectional surveys conducted in low- and middle-income countries. Among other measures, these gather socio-economic and demographic data on children under the age of five, along with other health and development measures. These surveys provide valuable information for comparing nutritional and health estimates within and across countries, monitoring trends, and measuring the progress of interventions targeting stunting among children. The resulting data contain both national and regional-level estimates for stunting indicators, essential for making inferences about national and regional trends. All data presented in the graphs as cross-sectional data points and tables are from the Tanzania DHS, 1991/92 - 2022 [15-21].

Anthropometry is commonly used to measure a child's nutritional status. Anthropometric measurements are used to report on child growth indicators. According to the World Health Organization (WHO) 2006 report, the distribution of height and weight among children under age 5 was compared with the WHO Child Growth Standards reference population [22]. The distribution of a well-nourished population will be similar to that of the reference population, while the distribution of a poorly nourished population will not. Children who are too short for their age are considered stunted, and this is the result of chronic or recurrent undernutrition. Stunting is the impaired growth and development that children experience due to poor nutrition, repeated infection, and inadequate psychosocial stimulation [23]. Children whose height-for-age z-score (HAZ) is below minus two standard deviations (−2 SD) from the median of the reference population are considered short for their age (stunted). Children whose HAZ is below minus three standard deviations (−3 SD) from the median are considered severely stunted [24, 25]. HAZ is calculated by subtracting an age- and sex-appropriate median value from a standard population, and dividing it by the SD of the standard population.

In 2006, the WHO recommended new standard growth cut-off points for stunting [22]. The Tanzania DHS surveys conducted from 1991/92 to 2004/05 used the Center for Disease Control standard

growth references, which were derived from the National Center for Health Statistics, Fels Research Institute, and Center for Disease Control reference population. To harmonize the surveys from 1991/2 to 2004/5, z-scores were recalculated according to the new WHO Child Growth Standards. The syntax file is available from the WHO (**http://www.who.int/childgrowth/software/en/**).

To maintaining the quality of measurements for the TDH surveys, quality control teams comprised personnel from the National Bureau of Statistics, the Office of the Chief Government Statistician, the Tanzania Food and Nutrition Centre (TFNC), and the ministry responsible for health in both mainland Tanzania and Zanzibar [26]. Monitoring of fieldwork involved regular visits to ensure the survey was conducted according to established protocols, providing real-time solutions to field challenges by observing the biomarker measurements of eligible respondents. All biomarker questionnaires and urine specimens were dispatched weekly to the nearest TFNC laboratory. Field check tables were regularly generated from Syncloud to monitor data quality and fieldwork progress. For field teams facing specific challenges, quality control staff provided targeted instructions to improve their performance; otherwise, consistent feedback was offered to all field teams.

## Data analysis

*Descriptive analysis*

Descriptive statistics were generated for the Tanzania DHS 1991/92-2022 survey data. We calculated frequencies and percentages, which are displayed using graphs as cross-sectional data points. Descriptive statistics were used to summarise factors influencing stunting, categorised into child-related factors (such as gender and age), caregiver/mother-related factors (such as education, number of ANC visits, and age at first birth), and household-level factors (such as gender of the household head and household wealth status). A chi-square test was used to compare observed results with expected results. The descriptive statistics guided the selection of covariates included in the univariate and multilevel binary logistic regression model to assess the factors influencing stunting in 2022.

*Empirical analysis*

It has been hypothesised that child stunting would be affected by both child, caregivers, and household-level factors. A multilevel binary logistic regression model was estimated to identify factors associated with stunting in 2022. Both backward and forward elimination have been used to arrive at the final model for statistically significant levels, conditional on the p-value being greater than 5 percent ($p > 0.05$). All analyses have been weighted using the weighting generated by the TDHS.

## Ethical considerations

Prior to data collection, the Institutional Review Board (IRB) and Tanzania's National Medical Research Committee approved the study protocol and DHS survey questionnaires. All participants provided written informed consent before the interviews. For illiterate individuals, a written consent form was presented orally in the presence of a legally permitted representative or witness, such as a friend, relative, or someone not affiliated with the research team. Parental or guardian consent was obtained for children and adolescents participating in the study. Every qualified respondent was questioned in the most private setting possible, without the presence of a third party.

## Results

## Trends of Stunting in Tanzania

In Tanzania, stunting has decreased slowly over the last 30 years, from 50% in 1991/92 to 30% in 2022 (see Figure 1A and Map 1). In Figure 1A, we can see that at least some of the policies and guidelines introduced over time have likely contributed to an improvement in the general trends in stunting in Tanzania; however, as shown in Map 1 in Figure 1, in 2022 stark disparities remained between regions. The regions in red may reflect inadequate government budget allocation, human capacity, or prioritization, or specific characteristics of the territory, like in the case of Iringa and Njombe, where the research highlights specific social factors that have been affecting the outcomes in

nutrition. Rates of stunting range from 57% in the Iringa region to less than 13% in Zanzibar (Mjini Magharibi). There are disparities in stunting rates across different areas, attributed to factors related to income. For example, people living in highland areas may not have easy access to clean water, healthcare, good infrastructure, or sanitation services, which can increase the likelihood of illness and poor nutrition [19]. Residents of villages like Iringa often do not purchase as much meat and fish during the dry season due to financial constraints or a preference for drinking over eating [20]. A lack of nutritional knowledge, poor childcare practices, inadequate breastfeeding, and excessive alcohol consumption by both parents have all been linked to high rates of stunting in Iringa [21]. Elisaria et al.'s (2020) qualitative research in the Njombe region found that women's heavy workloads, which leave them little time to prepare food, a lack of male support, limited control over animal foods such as chicken, alcoholism, domestic violence, and poor water, sanitation, and hygiene practices are significant social factors contributing to these issues [27].

## Characteristics associated with the risk of stunting (child, mother, and family)

### Stunting and Child Birthweight

Figure 2A shows the relationship between birthweight and stunting over time in Tanzania. Close to half of children under five years old who were born with low birthweight (<2,500 grams) are stunted, and only around a quarter of children who weighted 3,500 grams or more at birth. While stunting is a reflection of persistent undernutrition, weight at birth is very predictive of stunting [28, 29]. According to Mtongwa *et al.*, (2022), the comparative analysis of characteristics associated with the risk of stunting indicates that maternal age during pregnancy has a significant relationship with the likelihood of a child being born with low birth weight in Tanzania. The study states that mothers who were 18-24 years old had a higher percentage of having a low birth weight child compared with older mothers. This was related to poor nutrition during pregnancy and biological immaturity [30]. Low birthweight increases the likelihood of respiratory problems, reduced immune function, and metabolic

dysfunction, which in turn increases the risk of childhood infections, illnesses, and inadequate physical development, including stunting [31].

**Stunting and Child Gender**

Figure 1B shows this disparity over time in Tanzania, where, over the last 30 years, under-five boys have consistently had 4-7 percentage points (ppts) higher stunting rates than girls of the same age. Not only does stunting vary by region of residence, but there are also important and well-established differences by child characteristics. Across countries, child gender has been shown to be strongly associated with stunting rates, with male children being more likely to be stunted [30, 32-34]. It has been argued that this is driven by male children needing more calories than female children, even if the two are of the same size and weight, putting them at higher risk of stunting [35, 36]. This disparity can be explained through many studies conducted around the world, especially in Africa, examining the prevalence and factors associated with undernutrition between boys and girls. For example, in Gairo District in Morogoro, Tanzania, a cross-sectional study involving 300 under-five children and their mothers/caretakers identified factors associated with undernutrition in Morogoro [37]. One of the main results from the binary logistic regression model indicated that the sex of the child was significantly associated with stunting, meaning that relatively more male children were stunted compared with female children. The characteristics identified included favouritism towards daughters, male children being known to be hungrier than girls, which leads to early weaning and lasts for boys, and low socioeconomic status. Other reasons included gender roles, due to the time that female children spend at home, especially in the kitchen, making girls more nutritionally advantaged than boys [37].

**Stunting and Child Age**

Figure 1C highlights this pattern in Tanzania, where until approximately two years, stunting rates increase, and decrease thereafter. Following the literature on the relationship between stunting and child age in Tanzania, Mohamed argues that children aged 24-59 months, as shown in the graph, have a higher risk of being stunted compared with older children [37]. His hypothesis revolves around the

inadequate quality and quantity of food given to the children during the weaning period. In the study, the weaning food was mainly maize porridge, and the most common diseases were diarrhoea and intestinal worms, which is probably the reason for the increase in stunting in those ages. Additionally, the study shows an increase in stunting in children from 3-4 months who started solid foods, due to a correlation with maternal/caretaker age, age during pregnancy, and marital status [37]. Stunting rates across lower- and middle-income countries tend to increase rapidly with age until around 20 months, after which they stabilise or even reduce [38-40]. This has been linked to the longer exposure times and accumulation of adverse exposures to undernutrition and infections, while after this period, there can be some catch-up growth. Karlsson *et al., (2023)* assessed the correlation between the prevalence of stunting and age among children aged 0-59 months in 94 low- and middle-income countries [39]. The authors found that the prevalence of stunting was higher among older children, up to around 28 months of age. This can be attributed to longer exposure times and the cumulative effect of adverse factors such as undernutrition and infections. However, in the majority of countries, the prevalence of stunting decreased among older children after approximately 28 months. This can be attributed to the fact that further adverse factors have a less detrimental impact on older children, and they experience catch-up growth. In the first two years after birth, it is crucial to provide a high level of nutrition to support rapid growth and development. During this stage, any adverse factors have a greater impact on growth stunting. Furthermore, repeated infections during this period increase the likelihood of stunted growth. Although children above the age of two may undergo catch-up growth, stunting is often already firmly established by this point in many low- and middle-income countries [41]. A 15-year longitudinal study conducted in Ethiopia, India, Peru, and Vietnam revealed that a child's age plays a crucial role as a characteristics associated with risk of being stunted [42]. The study found that children at the ages of 8, 12, and 15 were less likely to experience stunting compared to those at the age of 1. However, it also showed that children at the age of 5 were more likely to be stunted than those at the age of 1 [42].

## Maternal characteristics associated with the risk of stunting

### Breastfeeding Practices

There has been a decline in stunting trend for those breastfeeding for more than 12 months in Tanzania, from 51% in 1991/92 to 38% in the 1996 DHS, while stunting rates for those breastfeeding for less than 12 months declined by 5 ppt from 22% in 1991/92 to 17% in 2022 (Figure 2B). The proportion of stunting among caregivers who breastfed for more than 12 months was higher than the overall stunting rates. While the evidence of the linkage between breastfeeding practices and stunting is mixed [38, 43, 44], breastfeeding practices play a crucial role in ensuring optimal nutrition and child development. Exclusive breastfeeding for the first six months of life provides essential nutrients, antibodies, and enzymes that promote growth and development. It protects against infections and diseases that can hinder physical and cognitive development. Continued breastfeeding, along with appropriate complementary foods for up to two years or beyond, ensures sustained nutritional support. Inadequate breastfeeding practices, such as early weaning or insufficient breastfeeding, can lead to nutrient deficiencies. WHO guidelines recommend exclusive breastfeeding for the first six months of life, and breastfeeding together with safe and appropriate complementary feeding for up to the age of two years and beyond. Tanzania has ratified the 1990 Innocenti Declaration [45] on the protection, promotion, and support of breastfeeding and has been commemorating World Breastfeeding Week every year. Furthermore, the Tanzanian Infant and Young Child Feeding Guidelines follow these WHO recommendations and the National Antenatal Care guidelines 2018 highlight that mothers should be counselled on exclusive breastfeeding and complementary feeding from 6 months during ANC visits and before discharge after birth [46, 47]. Despite this, exclusive breastfeeding rates stand at 64% for the first 6 months across Tanzania, slightly higher in urban areas (67%) than in rural areas (63%) [26]. Exclusive breastfeeding among children under 6 months has increased from 26% in 1991–92 to 64% in 2022 [26]. For example, a significant association was found between delays in the initiation of breastfeeding after childbirth and stunting. Children who were breastfed after one hour of birth were twice as likely to be stunted compared to those who breastfed within one hour of birth [38]. Conversely, Qadri *et al., (2017)* found that there was no association between breastfeeding and any

form of undernutrition [43]. Akombi *et al., (2017)* from Nigeria reported that a long duration of breastfeeding contributes to stunting [48], while Cetthakrikul *et al., (2018)* in Thailand found that prolonged breastfeeding beyond 12 months, when interacting with poor economic status of a household, potentiated stunting [49]. In Kilimanjaro, Tanzania, it was observed that exclusive breastfeeding practices for infants aged 0–6 months were not associated with stunting [44].

**Mother's Education**

Figure 2C shows the trend of stunting and maternal education in Tanzania. It is observed that mothers with no education have children with higher rates of stunting compared to those with higher education (primary complete). There has been a declining trend since the 1996 DHS for all education categories. For example, stunting among caregivers with no education stunting declined by 11 ppts from 47% to 36% in the 2022 DHS. In the research on Tanzania's undernutrition status, Mohamed argues that children whose mothers are more educated have lower chances of being wasted, due to the influence of education that makes mothers more informed about the nutritional benefits of food and their awareness of their children's growth process [37]. Likewise, Moshi's paper shows that mothers' education can predict being underweight, and mothers with lower education may miss out on important information about infant and young child feeding practices [6]. Educated mother is more likely to have a better income due to employment opportunities and better management of their income, allowing them to meet their children's needs. Moreover, an educated mother is likely to have better information to implement strategies for better nutrition, and due to the years, spent studying, they are more likely to have children at older ages, enabling them to provide better childcare.

More globally, the evidence reveals that one of the most consistent factors associated with childhood stunting over the last three decades has been mother-related factors such as low maternal education and nutritional status [28, 50-52]. Literature shows that children raised by educated caregivers have a lower risk of being stunted [28]. Semba *et al., (2008)* [51] argue that educated caregivers provide more care by ensuring that newborns are fully immunized and receive vitamin A and iodized salt; these, in turn, lower the risk of stunting for children under five years of age. Educated caregivers also

ensure better sanitation. Education is often referred to as an investment in human capital, and evidence has shown a positive correlation between the highest educational attainment within a family and total household well-being [53]. Caregivers who have received education are viewed as having proper maternal nutritional knowledge, including knowledge of a balanced diet.

**Stunting and Mother's Nutritional Status**

Mother's nutritional characteristics in Tanzania across the years have been consistently closely correlated with the growth of the newborn (Figure 2D). Evidence suggests that children of mothers with a low body mass index (BMI) have higher odds of being stunted [24, 30, 32, 33]. According to the National Antenatal Care Guidelines of 2018, women's BMI should be taken early in the first trimester and may be used as a baseline for follow-up of maternal weight gain during pregnancy [47]. The national guidelines on the Prevention and Control of Micronutrient Deficiencies promote key interventions addressing micronutrient deficiencies among women (including pregnant women) and adolescents, including micronutrient supplementation for pregnant and postpartum women during ANC, dietary diversification (e.g., home gardening/livestock keeping), and food fortification (industrial/home-based methods) [54]. In addition, the National Nutrition Strategy emphasizes promoting and supporting infant and young child-feeding practices, counselling on dietary improvement; provision of micronutrient supplements, and management of malnutrition [55]. Amaha *et al., (2021)* argue that a unit increase in BMI reduced the odds of child stunting by 4 ppts and every centimeter increase in maternal height reduced the odds of stunting by 0.5 ppts [34]. Similar to the results that Moshi found in the determinants of underweight among children in Tanzania, children born to mothers with a BMI lower than 18.5 were more likely to be underweight [6].

**Stunting and Number of ANC Visits**

Figure 3A shows the trend of the ANC visits over time. An increased number of ANC visits to more than 4 has been associated with a declining trend of stunting. A 16-point decline in stunting was observed for mothers having more than 4 ANC visits, from 42% in 1991-92 to 26% in 2022. Low birth weight is persistently recognised as a factor contributing to stunting. Improving the mother's

overall care is the most important determinant in reducing stunting levels in developing countries [34]. Generally, having at least 4 ANC clinic visits and delivering in a health facility are associated with lower severe stunting levels [30, 32-34]. According to the National Antenatal Care Guidelines of 2018, which is based on the 2016 WHO antenatal care model, a minimum of 8 ANC visits is recommended for a positive pregnancy experience [47]. The guidelines stipulate that all pregnant women should be counselled on healthy eating (balanced diet) and physical exercise for a healthy pregnancy. Furthermore, women should receive iron and folic acid supplementation, calcium supplementation where required, restrict caffeine intake, and regulate alcohol consumption. Other ANC nutrition-specific interventions include deworming tablets and malaria interventions, e.g., intermittent preventive treatment and long-lasting insecticidal nets. A study conducted in Northern Tanzania showed that the relative recurrence risk of low birth weight (LBW) was higher among women with less than four antenatal care visits, those with ≥37 weeks of gestational age, and those without preeclampsia in the first pregnancy [56].

## Household Related Factors

### *Stunting and Hygiene Practices*

Figure 3C shows the trends in stunting and household sanitation facilities. Stunting rates among households with unimproved sanitation and open defecation are higher than at the national level. According to the Moshi paper, 72% of the participants living in rural areas came from households with unimproved toilet facilities, finding that children from these households were more likely to be underweight due to increased vulnerabilities to infections [6]. Good hygiene practices correlate with a child’s health. The infant and Young Child Feeding guidelines stipulate that clean, safe preparation and feeding of complementary foods are essential to reduce the risk of contamination and related illnesses [46]. For clean and safe preparation of feeds, clean hands, utensils, safe water and food, and safe storage are required. For example, children whose mothers demonstrated good hand-washing practices were less likely to be stunted [57]. The use of clean and safe water reduces a child’s risk of infection. Furthermore, proper feeding practices are associated with lower risks of stunting [57].

Figure 3B shows trends in improved and unimproved water sources, with households using unimproved water sources being more disadvantaged compared to those with improved water.

***Stunting and Household's Socioeconomic Status (SES)***

Figure 3D shows a clear disparity in stunting rates between households of different socioeconomic statuses (SES), with households in the highest wealth quintile having lower stunting rates than those in the lowest wealth quintile. Evidence shows that poor socioeconomic status is a risk factor for stunting in LMICs [49, 57-60]. A recent study from LMICs, using DHS data from 72 countries, has shown that stunting is higher in the poorest than in the richest quartile, with large differences in stunting prevalence at 20 months; 50.3% in the poorest quartile being and 29.2% in the richest quartile [40]. Poor households are less likely to meet the nutritional needs of a caregiver and newborn. Caregivers living in underdeveloped areas and in poor housing with inadequate sanitary conditions have poorer health outcomes for the newborn.

*Stunting and Gender of Head of Household*

There is a declining stunting trend within male-headed households from 43% in 1991 to 30% in 2022. The proportion of stunting within female-headed households has been higher than that of male-headed households since 1991-2 to 2015-16 (Figure 4A). A previous national level study conducted using DHS data in Tanzania showed that child stunting was associated with female-headed households [10]. A study conducted using primary data to understand the prevalence of undernutrition and its characteristics in the lowland and highland areas of Kilosa District, Tanzania, highlighted that children living with mothers only and those from female-headed households were more likely to be stunted than those living with married mothers and in male-headed households [61]. Conversely, evidence from cross-country, cross-sectional quantitative data from the most recent DHS from eight sub-Saharan African countries Burundi (2016), Ethiopia (2016), Guinea (2018), Malawi (2016), Mali (2018), Zimbabwe (2015), Uganda (2016), and Tanzania (2015) showed that an increase in women's empowerment (specifically, women's autonomy in asset ownership and decision-making) significantly reduces anaemia and the co-occurrence of anaemia and stunting in children. However, children of

empowered women from male-headed households were more likely to be anaemic and concurrently anaemic and stunted compared to their counterparts whose mothers were from female-headed households [62]. Cross-sectional survey data collected from Bahi District in the Dodoma region and Mbarali District in the Mbeya region of Tanzania found that dietary diversity scores for children (1.6) and the minimum dietary diversity score for women (3.2) were lower in female-headed households compared to male-headed households. Moreover, children and women in female-headed households had low dietary diversity compared to those in male-headed households [63].

*Stunting and Child Infection (Diarrhoea)*

The prevalence of diarrhoea among children under 5 declined from 31% and 13% in 1991 to 8% and 9% respectively, in 2022. It has been observed that as diarrhoea declines over years, stunting also decreases. Trends of stunting and diarrhoea are presented in Figure 4B. Repeated episodes of sickness deteriorate a child's growth over time. Commonly reported illnesses include diarrhoeal episodes [32, 33, 48, 64-67], infections, and malaria affects child growth [68-70]. Diarrhoeal disease accounts for an estimated 13.5% of stunting globally [71]. Children experiencing episodes of diarrhoea do not have sufficient time to catch up on growth, which affects their subsequent development. Repeated diarrhoeal episodes are associated with stunting. It is important to note that repeated infections alongside nutritional deficiencies may lead to long-term linear growth faltering in children. In Ethiopia, children with malaria infections are more likely to be stunted [69]. The relationship between malaria and stunting is sometimes conflicting; for example, a review of 15 studies showed that stunting was associated with an increased malaria risk, while 11 studies showed no association, and 2 studies indicated protective effect of stunting [72].

**Stunting by Regions**

Figure 4C presents information on time trends in stunting in a few selected regions which were performing relatively well in 2022 (Dar es Salaam, Pwani, Kilimanjaro). Interestingly, whereas Dar es Salaam and Kilimanjaro have consistently been outperforming the average region in Tanzania in 1992-2022 in terms of stunting rates, Pwani started off as a relatively poor performer in 1992 but over

time has become a relatively well-performing region [73-75]. Figure 4D in turn presents information on trends in stunting in a few selected regions that were performing poorly in 2022 (Iringa, Ruvuma, and Mbeya). Note that all three regions have been performing relatively poorly ever since 1992. Research suggests that parents in those regions have been persistently giving children one or only two types of foods, completely ignoring other foods. For example, the Njombe region produces fruits and meat, but the only preferred menu for families consists of either ugali with beans or potatoes with beans [76]. The trends of stunting in the country portray a significant concern for child health and development.

## Bivariate Analysis of Childhood Stunting and Related Factors in 2022

To determine statistical significance of the differences described above, Table 1 presents bivariate regression results of 2022 stunting rates on various characteristics associated with risk of being stunted, including demographic characteristics such as age, place of residence, child gender, zone, mother's educational status, and wealth index quintile. The South West Highlands and Southern Highlands regions had a statistically significantly higher proportion of stunted children, at 37.8% and 46.2% respectively, compared to Eastern and Zanzibar, which had 22.8% and 17.1% respectively. A higher proportion of stunted children was observed among those aged 18 to 47 months (35.9%) and 9 to 17 months (27.7%). Similarly, males had a higher proportion (33.3%) of stunting compared to females (25.8%). A high proportion (55.6%) of stunting was observed in children with low birthweight. Women with short birth intervals (less than 24 months) had a higher proportion (35.3%) of stunted children compared to those with intervals of 24 to 47 months (29.5%) and those above 47 months (25.6%). Overweight and obese women had a lower proportion of stunted children (24.5% each) compared to those with normal weight (32.9%). Women with secondary education or above had a lower proportion of stunted children (20.3%) compared to those with no formal education (36.3%). Those with more than four ANC visits had a lower proportion of stunted children (26.5%). Women who gave birth before the age of 20 had a higher proportion of stunted children; similarly, those exposed to alcohol had a higher proportion of stunted children (38.3%). Women who used folic acid

for more than 90 days had a lower proportion of stunted children (27.8%). Households with limited access to improved water had a higher proportion of stunted children (34.9%); similarly, those lacking access to improved sanitation facilities (37.1%). Households in the lowest SES had the highest proportion of stunted children (38.6%) compared to those in the highest SES (12.6%).

**Table 1: Bivariate Analysis of Childhood Stunting and Related Factors, TDH 2022**

| | **Not on Stunted n=3,423** | **Stunted n=1,443** | **Total N = 4,866** | **χ2 (df)** | **P-value** |
|---|---|---|---|---|---|
| Zones new | n(%) | n(%) | n (%) | | |
| Lakes | 1,197(69.7) | 520(30.3) | 1,717(35.3) | 109.6(8) | 0.000 |
| Northern highlands | 369(74.0) | 129(25.9) | 498(10.2) | | |
| Central | 363(70.1) | 151(29.3) | 514(10.6) | | |
| Western | 328(70.3) | 138(29.7) | 466(9.6) | | |
| South West Highlands | 296(62.1) | 181(37.9) | 477(9.8) | | |
| Southern Highlands | 136(53.8) | 117(46.2) | 253(5.2) | | |
| Eastern | 471(77.2) | 139(22.8) | 610(12.5) | | |
| Southern | 143(77.1) | 43(22.9) | 186(3.8) | | |
| Zanzibar | 118(82.9) | 24(17.1) | 143(2.9) | | |
| child age | | | | | |
| <9 | 615(82.7) | 129(17.3) | 744(15.3) | 81.5(4) | 0.000 |
| 9 - 17 | 584(72.3) | 224(27.7) | 808(16.6) | | |
| 18 – 47 | 1,527(64.1) | 855(35.9) | 2,382(48.9) | | |
| 48 – 59 | 670(75.2) | 221(24.8) | 891(18.3) | | |
| Child sex | | | | | |
| Male | 1,653(66.7) | 826(33.3) | 2,480(50.9) | 18.2(1) | 0.000 |
| Female | 1,769(74.2) | 617(25.8) | 2,386(49.0) | | |
| Child weight | | | | | |
| Low birth weight | 82(44.4) | 103(55.6) | 185(3.8) | 52.3(2) | 0.000 |
| Normal | 1,628(72.4) | 619(27.6) | 2,248(46.2) | | |
| Child fever | | | | | |
| Yes | 376(70.1) | 161(29.9) | 537(11.0) | 0.17(1) | 0.679 |
| No | 3,046(70.4) | 1,282(29.6) | 4,328(88.9) | | |
| Child Diarrhoea | | | | | |
| Yes | 294(69.6) | 129(30.5) | 423(8.7) | 0.09(1) | 0.755 |
| No | 3,129(70.4) | 1,314(29.6) | 4,443(91.3) | | |

| | | | | | |
|---|---|---|---|---|---|
| Birth order | | | | | |
| 1 | 745(68.6) | 341(31.4) | 1,086(22.3) | 16.5(3) | 0.001 |
| 2-3 | 1,347(72.2) | 519(27.8) | 1,866(38.4) | | |
| 4-5 | 796(73.9) | 280(26.0) | 1,076(22.1) | | |
| Six and above | 535(63.9) | 302(36.1) | 837(17.2) | | |
| Birth interval | | | | | |
| < 24 months | 398(64.7) | 217(35.3) | 615(12.6) | 14.6(3) | 0.002 |
| 24 - 47 months | 1,322(70.5) | 554(29.5) | 1,877(38.6) | | |
| Above 47 months | 954(74.4) | 328(25.6) | 1,282(26.4) | | |
| BMI | | | | | |
| Underweight | 258(72.9) | 96(27.1) | 354(7.3) | 47.6(4) | 0.000 |
| Normal | 2,143(67.1) | 1,051(32.9) | 3,194(65.6) | | |
| Overweight | 660(75.5) | 214(24.5) | 874(17.9) | | |
| Obese | 350(81.8) | 213(24.5) | 428(8.8) | | |
| Education | | | | | |
| None | 667(63.7) | 380(36.3) | 1,047(21.5) | 60.9(2) | 0.000 |
| Primary | 1,955(69.5) | 859(30.5) | 2,815(57.8) | | |
| Secondary and above | 800(79.7) | 203(20.3) | 1,003(20.6) | | |
| ANC visits | | | | | |
| < 4 visits | 2,108(68.5) | 970(31.5) | 3,078(63.3) | 13.8(1) | 0.000 |
| > 4 visits | 1,314(73.5) | 473(26.5) | 1,787(36.7) | | |
| Delivery - caesarean section | | | | | |
| No | 3,221(70.5) | 1,348(29.5) | 4,569(93.9) | 2.3(1) | 0.129 |
| Yes | 202(67.9) | 95(32.0) | 297(6.1) | | |
| Age at birth | | | | | |
| <20 years | 1,983(68.1) | 929(31.9) | 2,912(59.8) | 8.7(2) | 0.013 |
| 20-24 | 1,137(73.2) | 416(26.8) | 1,554(31.9) | | |
| 25+ | 302(75.7) | 97(24.3) | 399(8.2) | | |
| Exposure to alcohol | | | | | |
| No | 3,286(70.7) | 1,358(29.2) | 4,645(95.5) | 8.3(1) | 0.004 |
| Yes | 136(61.7) | 85(38.3) | 221(4.5) | | |
| Used folic acid for more than 90 days | | | | | |
| No | 2,628(69.8) | 1,137(30.2) | 3,766(77.4) | 3.6(1) | 0.058 |
| Yes | 794(72.2) | 305(27.8) | 1,100(22.6) | | |
| Access to improved water | | | | | |
| No | 953(65.0) | 512(34.9) | 1,465(30.1) | 34.9(2) | 0.000 |
| Yes | 2,350(72.4) | 894(27.6) | 894(61.9) | | |
| Assess to improved sanitation | | | | | |
| Unimproved | 1,244(62.9) | 734(37.1) | 1,978(40.7) | 113.4(3) | 0.000 |
| Improved | 1,686(77.4) | 492(22.6) | 2,178(44.7) | | |
| Open defecation | 389(67.3) | 189(32.8) | 578(11.9) | | |
| Household head | | | | | |
| Female | 805(72.7) | 302(27.3) | 1,107(22.7) | 4.1(1) | 0.044 |
| Male | 2,618(69.6) | 1,140(30.3) | 3,758(77.2) | | |
| SES | | | | | |

| | | | | | |
|---|---|---|---|---|---|
| Lowest | 687(61.5) | 430(38.6) | 1,117(22.9) | 139.7(4) | 0.000 |
| Second | 633(61.5) | 333(34.5) | 966(19.8) | | |
| Middle | 659(65.5) | 295(30.9) | 954(19.6) | | |
| Fourth | 734(72.3) | 281(27.7) | 1,015(20.9) | | |
| Highest | 709(87.4) | 102(12.6) | 812(16.7) | | |

## Multilevel logistic regression on the characteristics associated with the risk of stunting in 2022

A total of 4,807 observations were included in the regression analysis (Table 2). The table presents the results of multilevel logistic regression analysis on factors associated with stunting in Tanzania.

***Multilevel logistic regression analysis results***

Child factors influencing stunting included age, gender, and birth weight. All these factors contributed to stunting, with children aged between 9 and 17 months (Adjusted Odds Ratio - AOR = 1.831 [95% CI: 1.356-2.470], p < 0.001), children aged between 18 and 47 months (AOR = 2.678 [95% CI: 2.072 – 3.460], p < 0.001), male children (AOR = 1.543 [95% CI: 1.315 – 1.811], p < 0.001), and low birth weight (AOR = 3.616 [95% CI: 2.284 – 5.727], p < 0.001). Maternal characteristics associated with risk of being stunted included a birth interval of 24 to 47 months (AOR = 0.754 [95% CI: 0.592 – 0.959], p < 0.001), mothers with normal weight (AOR = 1.403 [95% CI: 1.014 – 1.940], p < 0.005), secondary education and above (AOR = 0.731 [95% CI: 0.542 – 0.985], p < 0.005), delivery by caesarean section (AOR = 1.465 [95% CI: 1.061 – 2.022], p < 0.005), and maternal exposure to alcohol (AOR = 1.566 [95% CI: 1.092 – 2.246], p < 0.005). Household characteristics associated with risk of being stunted included the use of unimproved sanitation facilities (AOR = 1.341 [95% CI: 1.052 – 1.709], p < 0.005) and higher socioeconomic status (AOR = 0.269 [95% CI: 0.179 – 0.404], p < 0.001). Contextual characteristics included households from the south-western highlands' region (AOR = 1.407; 95% CI: 1.114-1.776, p < 0.005) and households residing in the southern highlands (AOR = 1.977; 95% CI: 1.510-2.589, p < 0.001).

*Multilevel regression and trend analysis findings*

Several factors in the multilevel analysis were significantly associated with stunting after controlling for confounding factors, similar to the trend analysis for the DHS 1991/92-2022 (Table 2). These variables included households residing in the South West Highlands region ($p < 0.005$) and the Southern Highlands ($p < 0.001$); child age ($p < 0.001$), child sex ($p < 0.001$), child birth weight ($p < 0.001$), birth interval ($p < 0.005$), mother's normal weight ($p < 0.005$), mother's education ($p < 0.005$), delivery by Caesarean section ($p < 0.005$), access to sanitation ($p < 0.005$), and socio-economic status (SES) ($p < 0.001$).

Factors such as child diarrhoea (AOR = 1.231 [95% CI: 0.914 – 1.657], p = 0.170), more than four ANC visits (AOR = 0.863 [95% CI: 0.698 – 1.067], p = 0.175), mother's age at birth of 20 – 24 years (AOR = 0.904 [95% CI: 0.754 – 1.084], p = 0.279), above 25 years of age (AOR = 0.993 [95% CI: 0.738 – 1.333], p = 0.170), and access to improved water (AOR = 1.004 [95% CI: 0.834 – 1.207], p = 0.968) lose statistical significance in the multilevel analysis.

**Table 2: Multilevel logistic regression on the characteristics associated with the risk of stunting, TDHS 2022**

| | **COR** | **95%CI** | **P-value** | **AOR** | **95%CI** | **P-value** |
|---|---|---|---|---|---|---|
| Zones new | | | | | | |
| Lakes | 1 | | | | | |
| Northern highlands | 0.807 | 0.602-1.083 | 0.153 | 0.843 | 0.621-1.143 | 0.271 |
| Central | 0.953 | 0.728-1.247 | 0.729 | 0.919 | 0.688-1.228 | 0.571 |
| Western | 0.975 | 0.744-1.277 | 0.853 | 0.810 | 0.611-1.074 | 0.143 |
| South West Highlands | 1.407 | 1.115-1.776 | 0.004 | 1.415 | 1.114-1.799 | 0.004 |
| Southern Highlands | 1.977 | 1.510-2.589 | 0.000 | 2.379 | 1.765-3.205 | 0.000 |
| Eastern | 0.681 | 0.511-0.905 | 0.008 | 0.947 | 0.699-1.283 | 0.725 |
| Southern | 0.683 | 0.453-1.030 | 0.069 | 0.614 | 0.398-0.948 | 0.028 |
| Zanzibar | 0.474 | 0.365-0.616 | 0.000 | 0.832 | 0.620-1.117 | 0.221 |
| Child age | | | | | | |
| <9 | 1 | | | | | |
| 9 - 17 | 1.831 | 1.356-2.470 | 0.000 | 1.880 | 1.368-2.584 | 0.000 |
| 18 - 47 | 2.678 | 2.072-3.460 | 0.000 | 3.056 | 2.305-4.051 | 0.000 |
| 48 - 59 | 1.574 | 1.172-2.113 | 0.003 | 1.969 | 1.377-2.816 | 0.000 |
| Child sex | | | | | | |
| Female | 1 | | | | | |
| Male | 1.434 | 1.229-1.673 | 0.000 | 1.543 | 1.315-1.811 | 0.000 |
| Child weight | | | | | | |

| | | | | | | |
|---|---|---|---|---|---|---|
| Normal | 1 | | | | | |
| Low birth weight | 3.297 | 2.197-4.947 | 0.0000 | 3.616 | 2.284-5.727 | 0.000 |
| Child fever | | | | | | |
| No | 1 | | | | | |
| Yes | 1.015 | 0.776-1.328 | 0.913 | | | |
| Child Diarrhoea | | | | | | |
| No | 1 | | | 1 | | |
| Yes | 1.042 | 0.790-1.374 | 0.769 | 1.231 | 0.914-1.657 | 0.170 |
| Bir order | | | | | | |
| <2 | 1 | | | | | |
| 2-3 | 0.842 | 0.684-1.037 | 0.107 | 0.757 | 0.093-6.146 | 0.795 |
| 4-5 | 0.769 | 0.608-0.971 | 0.027 | 0.588 | 0.072-4.825 | 0.621 |
| Six and above | 1.233 | 0.973-1.563 | 0.083 | 0.867 | 0.106-7.099 | 0.894 |
| Bir interval | | | | | | |
| < 24 months | 1 | | | | | |
| 24 - 47 months | 0.769 | 0.605-0.976 | 0.031 | 0.754 | 0.592-0.959 | 0.022 |
| Above 47 months | 0.631 | 0.489-0.814 | 0.000 | 0.749 | 0.567-0.990 | 0.042 |
| BMI | | | | | | |
| Underweight | 1 | | | | | |
| Normal | 1.319 | 0.961-1.810 | 0.086 | 1.403 | 1.014-1.940 | 0.041 |
| Overweight | 0.871 | 0.612-1.240 | 0.444 | 1.124 | 0.776-1.629 | 0.535 |
| Obese | 0.598 | 0.394-0.906 | 0.015 | 1.043 | 0.670-1.625 | 0.849 |
| Education | | | | | | |
| None | 1 | | | | | |
| Primary | 0.772 | 0.641-0.931 | 0.007 | 0.952 | 0.775-1.170 | 0.643 |
| Secondary and above | 0.446 | 0.352-0.566 | 0.000 | 0.731 | 0.542-0.985 | 0.039 |
| ANC visits and above | | | | | | |
| < 4 visits | 1 | | | | | |
| > 4 visits | 0.781 | 0.661-0.923 | 0.004 | 0.863 | 0.698-1.067 | 0.175 |
| Delivery Caesarean section | | | | | | |
| No | 0 | | | | | |
| Yes | 1.127 | 0.856-1.483 | 0.395 | 1.465 | 1.061-2.022 | 0.020 |
| Age at birth | | | | | | |
| <20 years | 1 | | | | | |
| 20-24 | 0.782 | 0.661-0.926 | 0.004 | 0.904 | 0.754-1.084 | 0.279 |
| 25+ | 0.685 | 0.522-0.897 | 0.006 | 0.993 | 0.738-1.333 | 0.961 |
| Exposure to alcohol | | | | | | |
| No | 1 | | | | | |
| Yes | 1.502 | 1.058-2.133 | 0.023 | 1.566 | 1.092-2.246 | 0.015 |
| Used folic acid for more than 90 days | | | | | | |
| No | 1 | | | | | |
| Yes | 0.889 | 0.733-1.079 | 0.236 | | | |
| Access to improved water | | | | | | |
| No | 1 | | | 1 | | |
| Yes | 0.709 | 0.602-0.832 | 0.000 | 1.004 | 0.834-1.207 | 0.968 |

| | | | | | | |
|---|---|---|---|---|---|---|
| Assess to sanitation | | | | | | |
| Improved | 1 | | | 1 | | |
| Unimproved | 2.019 | 1.697-2.404 | 0.000 | 1.341 | 1.052-1.709 | 0.018 |
| Open defecation | 1.662 | 1.299-2.125 | 0.000 | 1.006 | 0.723-1.401 | 0.972 |
| Household head | | | | | | |
| Female | 1 | | | | | |
| Male | 1.158 | 0.961-1.396 | 0.124 | | | |
| SES | | | | | | |
| Lowest | 1 | | | | | |
| Second | 0.839 | 0.686-1.026 | 0.088 | 0.746 | 0.595-0.934 | 0.011 |
| Middle | 0.714 | 0.572-0.891 | 0.003 | 0.691 | 0.526-0.907 | 0.008 |
| Fourth | 0.611 | 0.477-0.784 | 0.000 | 0.633 | 0.449-0.891 | 0.009 |
| Highest | 0.230 | 0.174-0.305 | 0.000 | 0.269 | 0.179-0.404 | 0.000 |
| Observations | | | | 4,807 | | |
| Wald chi2 (39) | | | | 337.9 | | |
| Prob > chi2 | | | | 0.000 | | |
| Pseudo R2 | | | | 0.086 | | |

**Note**: Both backward and forward elimination have been used to arrive at the final multilevel model for statistically significant levels, conditional on the p-value being greater than 5 percent ($p > 0.05$).

## Discussion

The synthesis aimed to examine trends in stunting rates among children under five in mainland Tanzania, identify factors associated with stunting among children under five years over time. Despite the high burden of stunting, a decline has been observed, from 50% in 1991/2 to 30% in 2022. Identified characteristics associated with risk of being stunted included child-related maternal, child, household and other factors.

**Starting with maternal factors,** birth spacing has been found to influence child outcomes, including nutritional status [77, 78]. A meta-analysis indicates that a short interpregnancy interval following delivery is a risk factor for preterm birth, low birth weight, being small for gestational age, and congenital abnormalities [79]. Nutrition plays a crucial role in the preconception and prenatal periods, significantly influencing fetal development [24, 30, 32, 33, 80-82]. Maternal undernutrition during early pregnancy may increase the risk of adverse outcomes, including intrauterine growth restriction [83, 84]. Whereas maternal educational backgrounds were significantly linked to stunting, as supported by other studies indicating that mothers with higher levels of education positively impact

child outcomes [28, 85-88]. The association between maternal education and childhood stunting can be attributed to the health and growth benefits that arise from improved resources and occupational status among educated mothers. Furthermore, high maternal education enhances a mother's ability to create a healthy environment conducive to the child's intrauterine development.

**Concerning child factors,** low birth weight of newborns was significantly associated with stunting. Ideally infants with low birth weight (<2,500 g) are at a greater risk of developing stunting [31, 89]. Furthermore, child gender has been strongly linked to stunting rates across different countries, with male children more likely to experience stunting [30, 32-34]. This may be due to male children requiring more calories than their female counterparts, even when they are of the same size and weight, more likely to be born preterm, thus increasing their risk of stunting [35, 36, 90, 91]. Child age influence was found to influence stunting [37] whereas stunting is less prevalent among infants under six months of age, primarily due to exclusive breastfeeding practices [92]. Although children above the age of two may undergo catch-up growth, stunting is often already firmly established by this point in many low- and middle-income countries [41].

**The household factors** include household social economic status was significant associated with stunting. Households with lower socioeconomic status often experience lower nutritional status and poor sanitary and hygiene conditions [93]. Poor households are less likely to meet the nutritional needs of both caregivers and newborns. Children from low-income households are at increased risk of growth failure due to inadequate nutrition, greater susceptibility to illness, difficulties in accessing essential health services, water as well as improved sanitation facilities [57, 94].

## Limitations

The study faced several limitations. First and foremost, the cross-sectional nature of the data collection restricts the ability to establish causal relationships between variables. Since the first DHS, there have been changes in methodology, e.g. cut-off points for stunting. To address this, all data were

analysed using the new WHO cut-off points for comparison. Additionally, there is a possibility that not all confounders were addressed since no primary data were collected. A review of the DHS database revealed that the data collection tool does not include information on responsive care, and there are only a limited number of variables related to the safety and security domain. Furthermore, the data is insufficient regarding social care services and the support provided by family and foster care compared to institutional care. Existing policies were not evaluated in relation to stunting; therefore, using this information to justify changes in stunting requires attention.

## Conclusion and Recommendations

The nation's stunting tendencies raise serious concerns about the development and health of children. These discrepancies stem from factors at the mother, family, community, and child levels. To combat stunting, eliminate structural obstacles including unequal roles, and empower marginalised groups, a multisectoral strategy is required. Furthermore, current regulations and standards place more emphasis on educating mothers about diet and health than they do on empowering them economically.

**Abbreviation**

| | |
|---|---|
| ANC | Antenatal Care |
| AOR | Adjusted Odds Ratio |
| COR | Crude Odds Ratio |
| BMI | Body Mass Index |
| DHS | Demographic and Health Survey |
| FYDP | National Five-Year Development Plan |
| GDP | Gross domestic product |
| HSSP | Health Sector Strategic Plan |
| IQ | Intelligence Quotient |
| LBW | Low Birth Weight |
| LMICs | Low- and Middle-Income Countries |

| | |
|---|---|
| NCDs | Noncommunicable diseases |
| NMNAP | National Multisectoral Nutrition Action Plan |
| PPTs | Percentage points |
| SES | Social Economic Status |
| SUN | Scaling Up Nutrition |
| TDHS | Tanzania Demographic Health Survey |
| UNICEF | United Nations Children's Fund |
| WASH | Water, sanitation and hygiene |
| WHO | World Health Organization |

## Declarations

### Acknowledgements

We would like to acknowledge the Demographic and Health Surveys (DHS) Program, ICF, 530 Gaither Road, Suite 500, Rockville, MD 20850, USA for granting us permission to use the Tanzania DHS 1991/92- 2022. We also acknowledge the support of the UK International Development from the UK government and New Zealand's Ministry of Foreign Affairs and Trade (MFAT) contract PO number 10087; however, the views expressed do not necessarily reflect the UK or New Zealand's government official policies.

### Ethics approval and consent to participate

Not applicable

### Consent for publication

Not applicable

### Availability of data and material

The dataset(s) supporting the conclusions of this article is owned by the Demographic and Health Surveys (DHS) Program, ICF, 530 Gaither Road, Suite 500, Rockville, MD 20850, USA and available upon request.

**Competing interests**

The authors declare that they have no competing interests.

**Funding**

This manuscript is an output of the Thrive programme. This programme was funded with UK International Development from the UK government and New Zealand's Ministry of Foreign Affairs and Trade (MFAT) contract PO number 10087; however, the views expressed do not necessarily reflect the UK or New Zealand's government official policies. Thrive is a multi-country research programme that aims to assist countries in translating knowledge of positive early childhood development into practical, scalable, low-cost programmes capable of transforming societies across multiple generations (https://thrivechildevidence.org/projects/ ).

**Authors' contributions**

EE, LE, and BC conceptualised the study. AK and LE contributed to data manipulation, analysis and interpretation. AK drafted the manuscript. BC and EN contributed to results interpretation and manuscript writing. The manuscript was critically evaluated by all writers, who then approved the final version.

## Bibliography


1. UNICEF. *UNICEF/WHO/The World Bank: Joint child malnutrition estimates (JME). Accessed on 21st October 2024 from* **https://www.who.int/teams/nutrition-and-food-safety/monitoring-nutritional-status-and-food-safety-and-events/joint-child-malnutrition-estimates**. 2023.
2. FAO, *FAO, IFAD, UNICEF, WFP and WHO. 2022. The State of Food Security and Nutrition in the World 2022. Repurposing food and agricultural policies to make healthy diets more affordable. Rome, FAO.* **https://doi.org/10.4060/cc0639en**. *Accessed from* **https://openknowledge.fao.org/server/api/core/bitstreams/67b1e9c7-1a7f-4dc6-a19e-f6472a4ea83a/content**. 2022.
3. UNICEF, WHO, and W. Bank., *UNICEF, WHO & World Bank. 2023. UNICEF-WHO-World Bank: Joint child malnutrition estimates - Levels and trends (2023 edition). [Cited 24 April 2023].* **https://data.unicef.org/resources/jme-report-2023**. 2023.
4. Zhu, W., et al., *Urban-Rural Disparities in the Magnitude and Determinants of Stunting among Children under Five in Tanzania: Based on Tanzania Demographic and Health Surveys 1991-2016. LID - 10.3390/ijerph18105184 [doi] LID - 5184.* (1660-4601 (Electronic)).
5. Gatica-Domínguez, G.A.-O., C.A.-O. Victora, and A.A.-O. Barros, *Ethnic inequalities and trends in stunting prevalence among Guatemalan children: an analysis using national health surveys 1995-2014.* (1475-9276 (Electronic)).
6. Moshi, C.C., et al., *Determinants of underweight among children aged 0–23 months in Tanzania.* Food Science & Nutrition, 2022. **10**(4): p. 1167-1174.
7. Sukmawati S, et al., *Stunting Prevention with Education and Nutrition in Pregnant Women: A Review of Literature. Open Access Maced J Med Sci. 2021 Oct 15; 9(T6):12-19.* **https://doi.org/10.3889/oamjms.2021.7350**. 2021.
8. Young, M.A.-O., et al., *Role of maternal preconception nutrition on offspring growth and risk of stunting across the first 1000 days in Vietnam: A prospective cohort study.* 2018(1932-6203 (Electronic)).
9. UNICEF, *Malnutrition in mothers soars by 25 per cent in crisis-hit countries, putting women and newborn babies at risk. Accessed on 21st October 2024 from* **https://www.unicef.org/press-releases/malnutrition-mothers-soars-25-cent-crisis-hit-countries-putting-women-and-newborn**. 2023.
10. Sunguya, B.F., et al., *Trends in prevalence and determinants of stunting in Tanzania: an analysis of Tanzania demographic health surveys (1991-2016).* 2019(1475-2891 (Electronic)).
11. URT, *National Multisectoral Nutrition Action Plan 2021/22 - 2025/26.* **https://www.pmo.go.tz/uploads/documents/sw-1646121553-NMNAP.pdf**. 2022.

12. Makoka, D. and P.K. Masibo, *Is there a threshold level of maternal education sufficient to reduce child undernutrition? Evidence from Malawi, Tanzania and Zimbabwe.* (1471-2431 (Electronic)).

13. Hiliza, J.N., et al., *Prevalence and Factors Associated with Stunting among Public Primary School Pupils in Kasulu District, Western Tanzania.* (2520-5285 (Electronic)).

14. Musheiguza, E., et al., *Inequalities in stunting among under-five children in Tanzania: decomposing the concentration indexes using demographic health surveys from 2004/5 to 2015/6.* International Journal for Equity in Health, 2021. **20**(1): p. 46.

15. NBS, *Bureau of Statistics [Tanzania] and Macro International Inc. 1997. Tanzania Demographic and Health Survey 1996. Calverton, Maryland: Bureau of Statistics and Macro International.* . 1992.

16. NBS, *Bureau of Statistics [Tanzania] and Macro International Inc. 1997. Tanzania Demographic and Health Survey 1996. Calverton, Maryland: Bureau of Statistics and Macro International.* . 1996.

17. NBS, *National Bureau of Statistics (NBS) [Tanzania] and ORC Macro.2005. Tanzania Demographic and Health Survey 2004-05. Dar es Salaam, Tanzania, 2005.* 2005.

18. NBS, *National Bureau of Statistics (NBS) [Tanzania] and ICF Macro. 2011. Tanzania Demographic and Health Survey 2010. Dar es Salaam, Tanzania: NBS and ICF Macro*. 2010.

19. MoHCDGEC, *Ministry of Health Community Development, Gender, Elderly and Children - MoHCDGEC/Tanzania Mainland, Ministry of Health - MoH/Zanzibar, National Bureau of Statistics - NBS/Tanzania, et al. Tanzania demographic and health survey and malaria indicator survey 2015-16. Dar es Salaam: MoHCDGEC, MoH, NBS, OCGS, and ICF; 2016*. 2016.

20. MoH, *Ministry of Health (MoH) [Tanzania Mainland], Ministry of Health (MoH) [Zanzibar], National Bureau of Statistics (NBS), Office of the Chief Government Statistician (OCGS), and ICF. 2023 Tanzania Demographic and Health Survey and Malaria Indicator Survey 2022 Key Indicators Report. Dodoma, Tanzania, and Rockville, Maryland, USA: MoH, NBS, OCGS, and ICF*. 2022.

21. NBS, *National Bureau of Statistics [Tanzania] and Macro International Inc. 2000. Tanzania Reproductive and Child Health Survey 1999. Calverton, Maryland: National Bureau of Statistics and Macro International Inc.* 1999.

22. WHO, *WHO Child Growth Standards based on length/height, weight and age*. 2006(0803-5326 (Print)).

23. WHO, *WHO. Global nutrition targets 2025: stunting policy brief (WHO/NMH/NHD/14.3). Geneva: World Health Organization; 2014*. 2014.

24. Sinha, R.K., et al., *Determinants of Stunting, Wasting, and Underweight in Five High-Burden Pockets of Four Indian States.* (0970-0218 (Print)).

25. WHO, *Physical status: the use and interpretation of anthropometry. Report of a WHO Expert Committee.* 2006(0512-3054 (Print)).
26. TDHS, *Ministry of Health (MoH) [Tanzania Mainland], Ministry of Health (MoH) [Zanzibar], National Bureau of Statistics (NBS), Office of the Chief Government Statistician (OCGS), and ICF. 2023 Tanzania Demographic and Health Survey and Malaria Indicator Survey 2022 Key Indicators Report. Dodoma, Tanzania, and Rockville, Maryland, USA: MoH, NBS, OCGS, and ICF.* 2022.
27. Elisaria, E., et al., *Improving maternal and adolescent nutrition in Tanzania. Situational Analysis. Dar es Salaam, Tanzania.* 2020.
28. Mulyaningsih, T., et al., *Beyond personal factors: Multilevel determinants of childhood stunting in Indonesia. PLoS ONE 16(11): e0260265.* **https://doi.org/10.1371/journal.pone.0260265**. 2021.
29. Tiwari, R., L. Ausman, and K. Agho, *Determinants of stunting and severe stunting among under-fives: Evidence from the 2011 Nepal Demographic and Health Survey. BMC Pediatrics. 2014 Sep 27; 14(1).* **https://doi.org/10.1186/1471-2431-14-239** *PMID: 25262003.* 2014.
30. Mtongwa, R.H., C. Festo, and E. Elisaria, *A comparative analysis of determinants of low birth weight and stunting among under five children of adolescent and non-adolescent mothers using 2015/16 Tanzania Demographic and Health Survey (TDHS).* BMC Nutrition, 2021. **7**(1): p. 64.
31. Jana, A., D. Dey, and R. Ghosh, *Contribution of low birth weight to childhood undernutrition in India: evidence from the national family health survey 2019–2021.* BMC Public Health, 2023. **23**(1): p. 1336.
32. Das, S., et al., *Determinants of stunting among children under 2 years in urban informal settlements in Mumbai, India: evidence from a household census.* Journal of Health, Population and Nutrition, 2020. **39**(1): p. 10.
33. Islam, M.A.-O., et al., *Determinants of stunting during the first 1,000 days of life in Bangladesh: A review.* (2048-7177 (Print)).
34. Amaha, N.A.-O. and B.T. Woldeamanuel, *Maternal factors associated with moderate and severe stunting in Ethiopian children: analysis of some environmental factors based on 2016 demographic health survey.* 2021(1475-2891 (Electronic)).
35. Samuel, A., et al., *Gender differences in nutritional status and determinants among infants (6–11 m): a cross-sectional study in two regions in Ethiopia.* BMC Public Health, 2022. **22**(1): p. 401.
36. Thompson, A.L., *Greater male vulnerability to stunting? Evaluating sex differences in growth, pathways and biocultural mechanisms.* (1464-5033 (Electronic)).
37. Mohamed T. and C. Nyaruhucha, *Household and Community Factors Affecting Nutritional Status of Under- five Children (6-59 months) in Gairo District Using Composite Index of*

*Anthropometric Failure. Tanzania Journal of Science 49(1): 76-85, 2023. ISSN 0856-1761, e-ISSN 2507-7961*. 2023.

38. Muchina, E. and P. Waithaka, *Relationship between breastfeeding practices and nutritional status of children aged 0-24 months in Nairobi, Kenya. Accessed on 25th January 2024 from* **https://www.ajol.info/index.php/ajfand/article/view/55329**. 2010.
39. Karlsson, O., et al., *Patterns in child stunting by age: A cross-sectional study of 94 low- and middle-income countries.* Maternal & Child Nutrition, 2023. **19**(4): p. e13537.
40. Bommer, C., S. Vollmer, and S.V. Subramanian, *How socioeconomic status moderates the stunting-age relationship in low-income and middle-income countries.* (2059-7908 (Print)).
41. Roth, D.E., et al., *Early childhood linear growth faltering in low-income and middle-income countries as a whole-population condition: analysis of 179 Demographic and Health Surveys from 64 countries (1993-2015).* 2017(2214-109X (Electronic)).
42. Wake, S.K., et al., *Longitudinal trends and determinants of stunting among children aged 1–15 years.* Archives of Public Health, 2023. **81**(1): p. 60.
43. Qadri, S., H. Hubaira, and D. Singh, *A study of inter-relationship between prolonged breast feeding and nutritional status in children aged 15 months to 3 years: An observational, case-control study.* Sri Lanka Journal of Child Health, 2017. **46**(4): p. 362-365.
44. Mgongo, M., et al., *Underweight, Stunting and Wasting among Children in Kilimanjaro Region, Tanzania; a Population-Based Cross-Sectional Study. LID - 10.3390/ijerph14050509 [doi] LID - 509.* (1660-4601 (Electronic)).
45. WABA. *World Alliance for Breastfeeding Action (WABA). 1990 Innocenti Declaration On The Protection, Promotion And Support Of Breastfeeding. Accessed on 10th October 2024 from* **https://waba.org.my/1990-innocenti-declaration-on-the-protection-promotion-and-support-of-breastfeeding/**. 2024.
46. MoHSW, *MoHSW The United Republic of Tanzania Ministry of Health and Social Welfare. National Guidelines Infant and Young Child Feeding. Dar es Salaam, Tanzania.* 2013.
47. MOHCDGEC, *The Ministry of Health Community Development Gender Elderly and Children (MOHCDGEC). Antenatal Care Guidelines. Dar es Salaam. Tanzania.* 2018.
48. Akombi, B.J., et al., *Stunting, Wasting and Underweight in Sub-Saharan Africa: A Systematic Review. LID - 10.3390/ijerph14080863 [doi] LID - 863.* 2017(1660-4601 (Electronic)).
49. Cetthakrikul, N., et al., *Childhood stunting in Thailand: when prolonged breastfeeding interacts with household poverty.* BMC Pediatrics, 2018. **18**(1): p. 395.
50. Elmighrabi, N.F., C.A.-O. Fleming, and K.A.-O. Agho, *Factors Associated with Childhood Stunting in Four North African Countries: Evidence from Multiple Indicator Cluster Surveys, 2014-2019. LID - 10.3390/nu16040473 [doi] LID - 473.* (2072-6643 (Electronic)).

51. Semba, R., et al., *Effect of parental formal education on risk of child stunting in Indonesia and Bangladesh: a cross-sectional study. The Lancet. 2008 Jan 26; 371 (9609):322–8.* **https://doi.org/10.1016/S0140-6736(08)60169-5** *PMID: 18294999.* 2008.
52. Balla, S., et al., *Progress in child stunting across the world from 1990 to 2015: testing the global convergence hypothesis.* (1475-2727 (Electronic)).
53. Stryzhak, O., *The relationship between education, income, economic freedom and happiness.* 2020.
54. MoH, *National Guidelines on the Prevention and Control of Micronutrient Deficiencies. Dar es Salaam, Tanzania* 2018.
55. MoHSW, *National Nutrition Strategy (NNS) (2011/12–2015/16). Dar es Salaam, Tanzania.* 2011.
56. Mvunta, M.H., et al., *Incidence and recurrence risk of low birth weight in Northern Tanzania: A registry based study.* PLOS ONE, 2019. **14**(4): p. e0215768.
57. Habimana, J.A.-O.X., et al., *Prevalence and Correlates of Stunting among Children Aged 6-23 Months from Poor Households in Rwanda. LID - 10.3390/ijerph20054068 [doi] LID - 4068.* (1660-4601 (Electronic)).
58. Nshimyiryo, A.A.-O., et al., *Risk factors for stunting among children under five years: a cross-sectional population-based study in Rwanda using the 2015 Demographic and Health Survey.* (1471-2458 (Electronic)).
59. Muche, A.A.-O. and R. Dewau, *Severe stunting and its associated factors among children aged 6-59 months in Ethiopia; multilevel ordinal logistic regression model.* (1824-7288 (Electronic)).
60. Shinsugi, C., et al., *Factors associated with stunting among children according to the level of food insecurity in the household: a cross-sectional study in a rural community of Southeastern Kenya.* BMC Public Health, 2015. **15**(1): p. 441.
61. Mrema, J., et al., *"Prevalence and Determinants of Undernutrition among 6- to 59-Months-Old Children in Lowland and Highland Areas in Kilosa District, Tanzania: A Cross-Sectional Study", Journal of Nutrition and Metabolism, vol. 2021, Article ID 6627557, 9 pages, 2021.* **https://doi.org/10.1155/2021/6627557**.
62. Christian, A.K., et al., *Women's empowerment, children's nutritional status, and the mediating role of household headship structure: Evidence from sub-Saharan Africa.* Maternal & Child Nutrition, 2023. **19**(3): p. e13520.
63. Ochieng, J., et al., *Determinants of dietary diversity and the potential role of men in improving household nutrition in Tanzania.* PLOS ONE, 2017. **12**(12): p. e0189022.
64. Tafese, Z.A.-O., et al., *Child Feeding Practice and Primary Health Care as Major Correlates of Stunting and Underweight among 6- to 23-Month-Old Infants and Young Children in Food-Insecure Households in Ethiopia.* (2475-2991 (Electronic)).

65. Mrema, J.D., C.N. Nyaruhucha, and A.W. Mwanri, *Dietary intake and diversity among children of age 6–59 months in lowland and highland areas in Kilosa District in Morogoro, Tanzania.* Human Nutrition & Metabolism, 2023. **33**: p. 200205.
66. Beal, T.A.-O., et al., *A review of child stunting determinants in Indonesia.* (1740-8709 (Electronic)).
67. Kalinda, C.A.-O., et al., *Understanding factors associated with rural-urban disparities of stunting among under-five children in Rwanda: A decomposition analysis approach.* (1740-8709 (Electronic)).
68. Ateba, F.F., et al., *The effect of malaria on stunting: an instrumental variables approach.* Transactions of The Royal Society of Tropical Medicine and Hygiene, 2021. **115**(9): p. 1094-1098.
69. Gari, T.A.-O., et al., *Malaria increased the risk of stunting and wasting among young children in Ethiopia: Results of a cohort study.* (1932-6203 (Electronic)).
70. Jackson, B.D. and R.E. Black, *A Literature Review of the Effect of Malaria on Stunting.* The Journal of Nutrition, 2017. **147**(11): p. 2163S-2168S.
71. Danaei, G., et al., *Risk Factors for Childhood Stunting in 137 Developing Countries: A Comparative Risk Assessment Analysis at Global, Regional, and Country Levels.* (1549-1676 (Electronic)).
72. Das, D., et al., *Complex interactions between malaria and malnutrition: a systematic literature review.* BMC Medicine, 2018. **16**(1): p. 186.
73. Ledama, U., *Groundbreaking 'Stawisha Maisha' Programme to combat child malnutrition launched. Accessed from* **https://www.unicef.org/tanzania/press-releases/groundbreaking-stawisha-maisha-programme-combat-child-malnutrition-launched**. 2024.
74. Blakstad, M.A.-O.X., et al., *Home gardening improves dietary diversity, a cluster-randomized controlled trial among Tanzanian women.* (1740-8709 (Electronic)).
75. Juma, O.A., et al., *Prevalence and assessment of malnutrition among children attending the Reproductive and Child Health clinic at Bagamoyo District Hospital, Tanzania.* BMC Public Health, 2016. **16**(1): p. 1094.
76. Malima, W. *Experts outline factors behind stunting in Njombe. Accessed on 10th October 2024 from* **https://dailynews.co.tz/experts-outline-factors-behind-stunting-in-njombe/**. 2024.
77. Dhamrait, G., et al., *The effects of birth spacing on early childhood development in high-income nations: A systematic review.* (2296-2360 (Print)).
78. Dhamrait, G.A.-O.X., C.A.-O. Taylor, and G.A.-O. Pereira, *Interpregnancy intervals and child development at age 5: a population data linkage study.* (2044-6055 (Electronic)).
79. Wang, Y., et al., *Short interpregnancy interval can lead to adverse pregnancy outcomes: A meta-analysis.* (2296-858X (Print)).

80. Milner, E.A.-O., et al., *Timing, intensity, and duration of household food insecurity are associated with early childhood development in Kenya.* 2017(1740-8709 (Electronic)).
81. Naaz, A. and K.N. Muneshwar, *How Maternal Nutritional and Mental Health Affects Child Health During Pregnancy: A Narrative Review.* 2023(2168-8184 (Print)).
82. Gogos, A., et al., *Socioeconomic adversity, maternal nutrition, and the prenatal programming of offspring cognition and language at two years of age through maternal inflammation.* Brain, Behavior, and Immunity, 2024.
83. Haque, R., et al., *Nexus between maternal underweight and child anthropometric status in South and South-East Asian countries.* Nutrition, 2022. **98**: p. 111628.
84. Woldeamanuel, G.A.-O., et al., *Effect of nutritional status of pregnant women on birth weight of newborns at Butajira Referral Hospital, Butajira, Ethiopia.* (2050-3121 (Print)).
85. Ertem, I.O., et al., *Mothers' knowledge of young child development in a developing country.* (0305-1862 (Print)).
86. Hasan, M.N., et al., *Early childhood developmental status and its associated factors in Bangladesh: a comparison of two consecutive nationally representative surveys.* BMC Public Health, 2023. **23**(1): p. 687.
87. Gizaw, B. and S. Gebremedhin, *Factors associated with low birthweight in North Shewa zone, Central Ethiopia: case-control study.* Italian Journal of Pediatrics, 2018. **44**(1): p. 76.
88. Casale, D., G. Espi, and S.A. Norris, *Estimating the pathways through which maternal education affects stunting: evidence from an urban cohort in South Africa.* (1475-2727 (Electronic)).
89. Santosa, A., E. Novanda Arif, and D. Abdul Ghoni, *Effect of maternal and child factors on stunting: partial least squares structural equation modeling.* (2713-4148 (Electronic)).
90. Pongou, R., *Why is infant mortality higher in boys than in girls? A new hypothesis based on preconception environment and evidence from a large sample of twins.* (0070-3370 (Print)).
91. Galante, L.A.-O., et al., *Sex-Specific Human Milk Composition: The Role of Infant Sex in Determining Early Life Nutrition. LID - 10.3390/nu10091194 [doi] LID - 1194.* (2072-6643 (Electronic)).
92. Ricci, J.A. and S. Becker, *Risk factors for wasting and stunting among children in Metro Cebu, Philippines.* (0002-9165 (Print)).
93. Ribe, I.G., et al., *Determinants of early child development in rural Tanzania.* Child and Adolescent Psychiatry and Mental Health, 2018. **12**(1): p. 18.
94. Akombi, B.J., et al., *Stunting and severe stunting among children under-5 years in Nigeria: A multilevel analysis. BMC pediatrics, 17, 1-16.* 2017.

# Figure 1: Stunting, child gender and child age from TDHS 1991/92-2022.

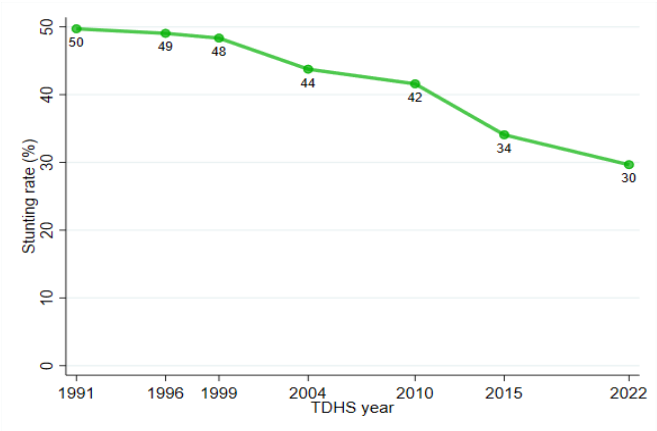


Figure 1 A: Thirty Years Stunting 1991/92 – 2022

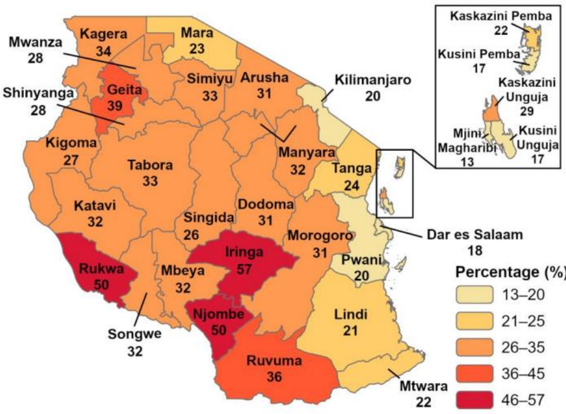


Map 1: Stunting Across Regions from 2022 TDHS Report

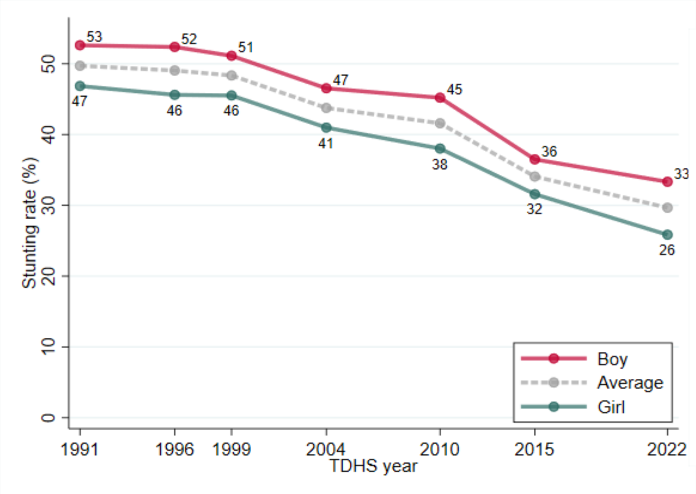


Figure 1 B: Stunting Among Boys and Girls

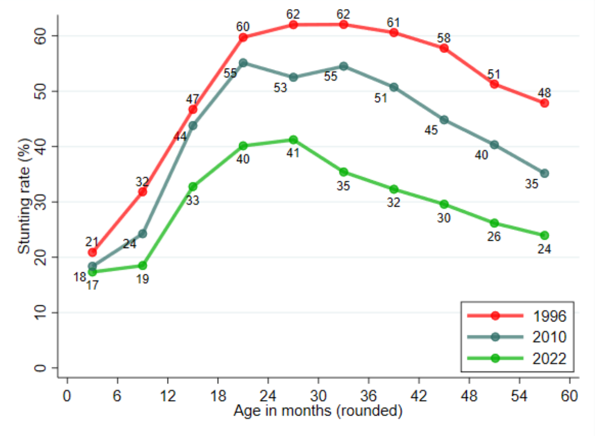


Figure 1 C: Stunting and Child Age

**Note: The line graphs represent cross-sectional data points for different TDHS from 1991/92 to 2022, rather than longitudinal data.**

## Figure 2: Stunting, child weight, breast feeding, mother education and mother's BMI - from TDHS 1991/92 – 2022

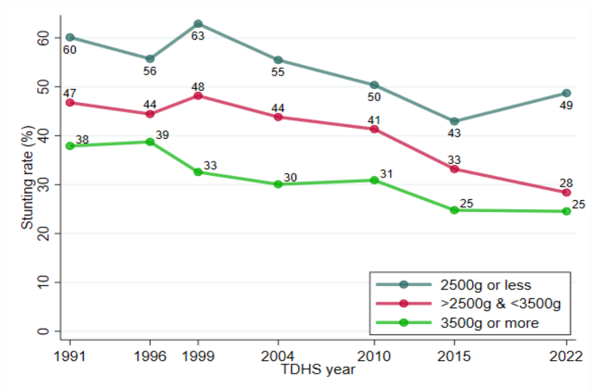


Figure 2 A: Stunting and Child Birthweight

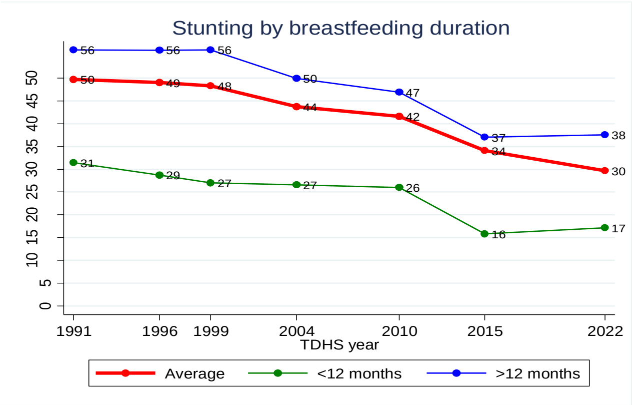


Figure 2 B: Stunting and Breastfeeding Duration

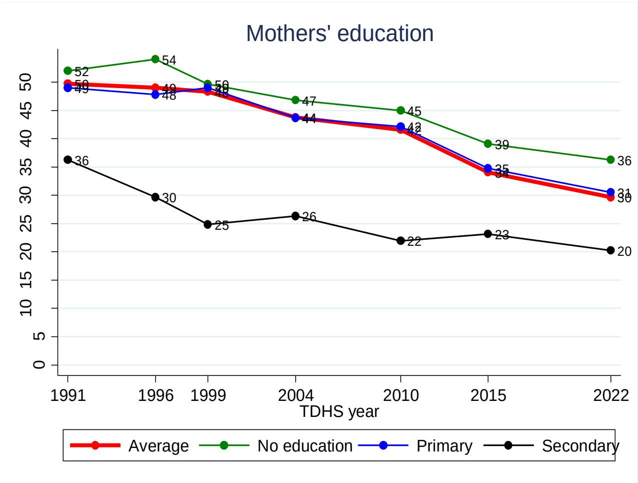


Figure 2 C: Stunting and Mother's Education Level

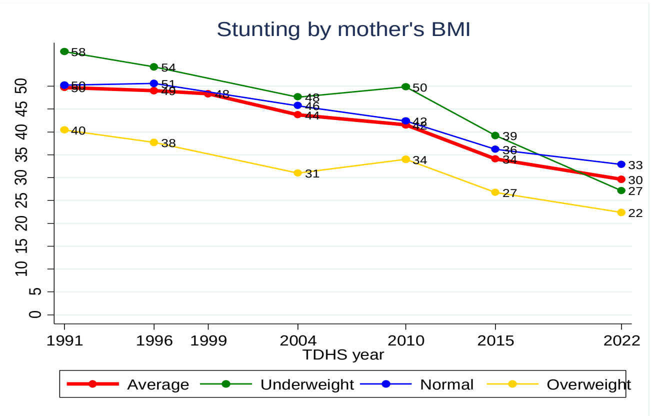


Figure 2 D: Stunting and mothers' BMI

**Note: The line graphs represent cross-sectional data points for different TDHS from 1991/92 to 2022, rather than longitudinal data.**

## Figure 3: Stunting, ANC visits, Access to Water, Sanitation and Socio-economic Status -    from TDHS 1991/92 – 2022

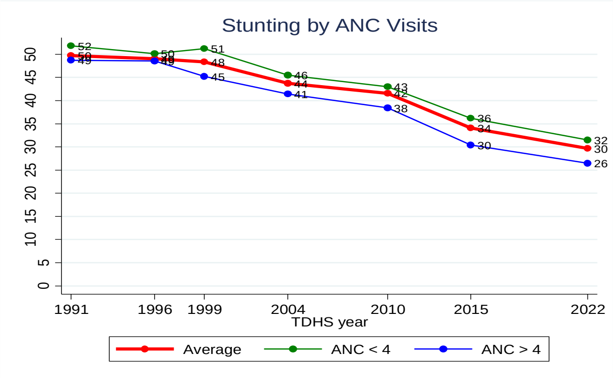


Figure 3 A: Stunting and ANC visits

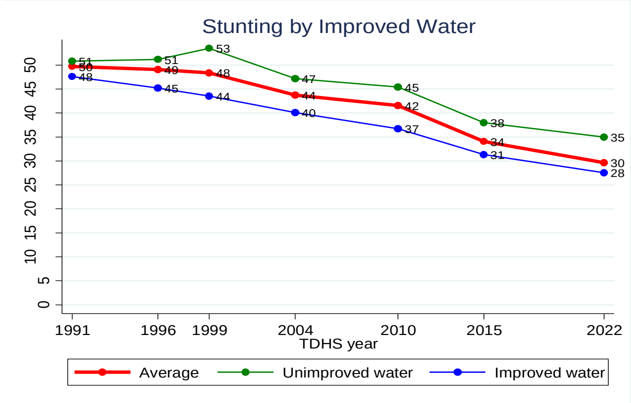


Figure 3 B: Stunting and Access to Water

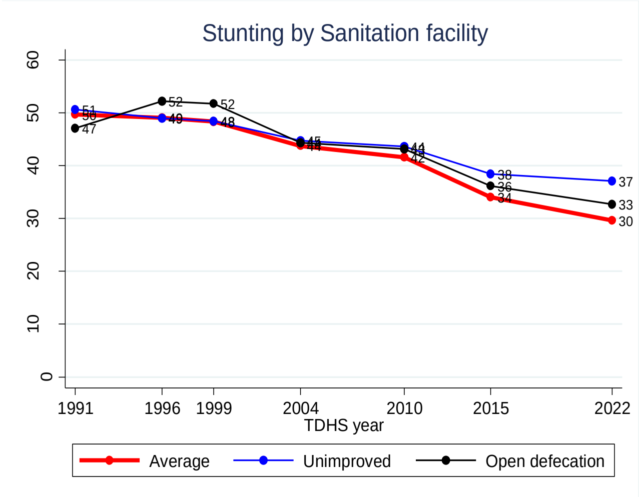


Figure 3 C: Stunting and Sanitation Facility

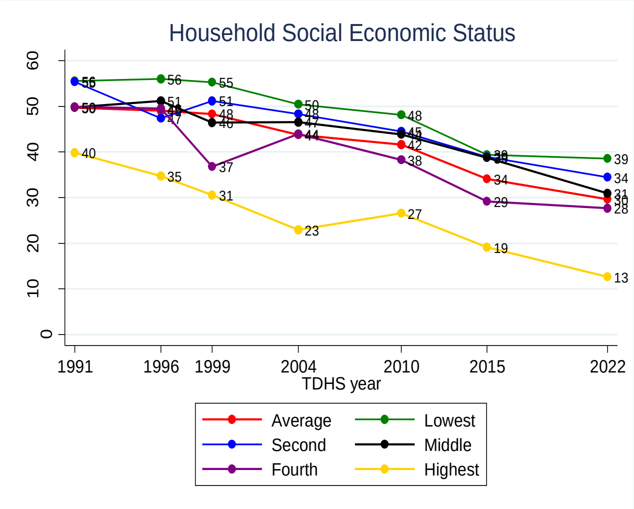


Figure 3 D: Stunting and Household Socio-economic Status

**Note: The line graphs represent cross-sectional data points for different TDHS from 1991/92 to 2022, rather than longitudinal data.**

## Figure 4: Stunting, Household Head Gender, Diarrhoea, Well and Poor Performing Regions from TDHS - 1991/92 – 2022

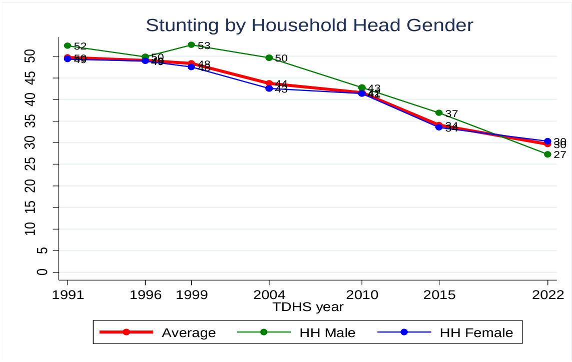


Figure 4 A: Stunting and Household Head Gender

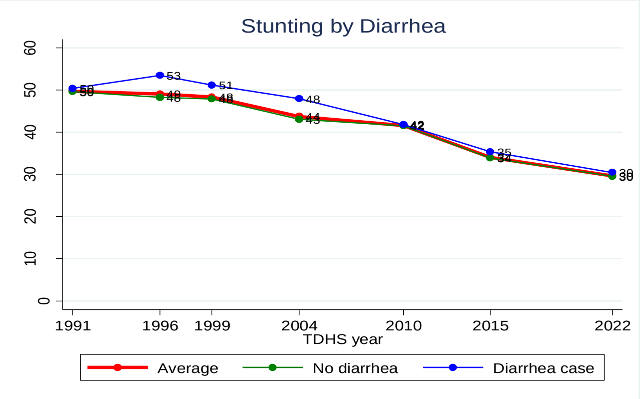


Figure 4 B: Stunting and Diarrhoea

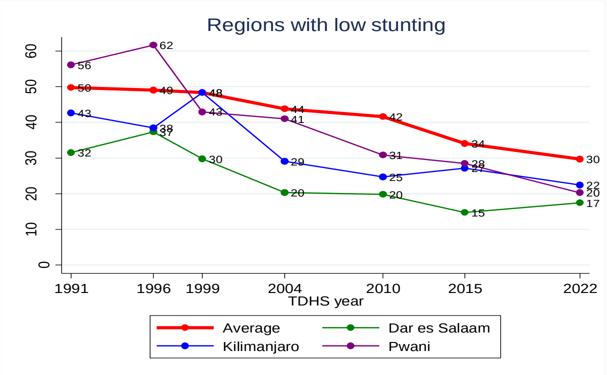


Figure 4 C: Stunting for Well-Performing Regions

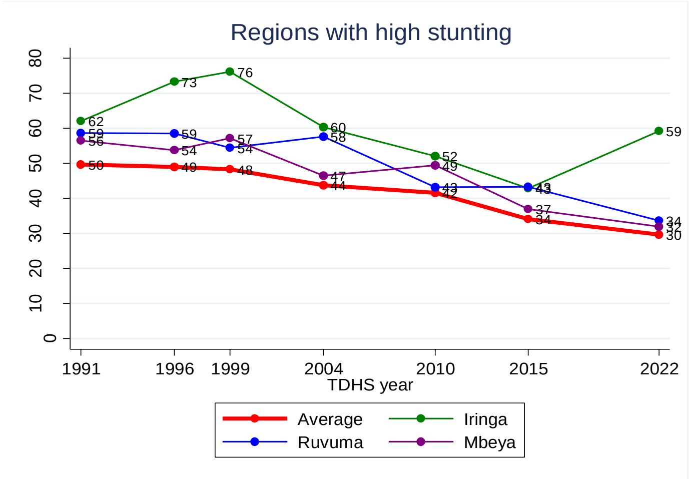


Figure 4 D: Stunting for Poor Performing Regions

**Note: The line graphs represent cross-sectional data points for different TDHS from 1991/92 to 2022, rather than longitudinal data.**
