# Supplementary material for: Thirty years of declining stunting in Tanzania: Trends and ongoing challenges
Source: PLoS One. 2025 Jul 29;20(7):e0327779. doi: 10.1371/journal.pone.0327779 (PMC12306788; doi:10.1371/journal.pone.0327779)
Supplement: Attachment — Submitted filename: Peer review report for the manuscript by Elisaria et al.docx [file pone.0327779.s001.docx]

**Peer review report for the manuscript “Thirty Years of Stunting in Tanzania” by Elisaria et al.**

The authors are to be credited for their thorough study on stunting, particularly for providing valuable evidence on stunting trends over the years. The presentation of these trends across different characteristics is impressive and shows notable improvements. However, despite these strengths, the authors should consider revising their work in the following areas:

**Title**

- The title "Thirty Years of Stunting in Tanzania" may not fully capture the significant positive trends observed in the study, particularly the decline in stunting rates from 50% in 1991/92 to 30% in 2022. To better reflect these encouraging findings, the title could emphasize the reduction in stunting, such as "Declining Stunting Rates in Tanzania: A 30-Year Overview" or "Tracking 30 Years of Declining Stunting in Tanzania." This would more clearly communicate the positive trend of stunting reduction over the three decades. However, it is also important to highlight that despite this progress, challenges remain, and continued efforts are necessary to further reduce stunting, especially in underprivileged communities. A revised title like "Thirty Years of Declining Stunting in Tanzania: Trends and Ongoing Challenges" could better capture both the progress made and the need for targeted interventions to address remaining disparities.

Abstract

- Please ensure the methods section of the abstract specifies the years of the TDHS datasets used in the study.
- In the methods or results sections of the abstract, the sample size(s) should be indicated.
- In the results section of the abstract, the term "SES" already stands for "socioeconomic status," so the use of "SES status" appears redundant and should be revised to simply "SES".
- Revise “… was significant associated with risk of being stunting” to something like “… was significantly associated with the risk of being stunted”.
- The conclusion section of the abstract needs revision. Unnecessary information can be reduced to make it concise. For example, the first two sentences could be revised to something like “Stunting remains a major challenge in Tanzania, driven by factors at the maternal, family, community, and child levels.”
- The keyword “Demographic Health Survey” should be “Demographic and Health Survey”, referring to DHS.

Introduction

- Revise the first sentence of the introduction to something like “In 2022, an estimated 148.1 million children under the age of five globally were classified as stunted, ...”
- “Demographic Health Surveys” should be “Demographic and Health Surveys”
- The sentence on page 3 “…data collected from 1991/92-2022 indicates the country had a decrease in stunting from 50% in 1991/92 to 30% [2] …” should indicate when the 30% was the case. In the abstract, it is stated that 30% was the case in 2022. Please revise accordingly.
- Please revise the entire manuscript to correct any grammar and sentence structure issues. For example, the sentence “The World Bank and UNICEF agree that heightened efforts needed to reach the global goal of reducing the number of children suffering from stunting to 89 million by 2030 [1]” seems to be missing the word “are,” which affects the sentence’s clarity and flow.

Materials and methods

- The methods section requires should be reworked. The authors should consider the following to improve the section.
- Did the authors use the Tanzania DHS data, 1991/92 – 2022 as secondary data or primary data?
- How was the data accessed? If the standard procedure of accessing such data through the DHS Program (<https://dhsprogram.com/>) was (or wasn’t) used, it should be made explicit.
- Consider adding more subtitles. For example, you have indicated in the data source that this study was based on multiple cross-sectional data points. This can appear under a separate subtitle – Study design – and indicate that this was a repeated cross-sectional study or serial cross-sectional study.
- Then we need to see how many data points were used over the mentioned period, 1991/92 – 2022.
- Please clarify the variables used, both dependent and independent. For instance, the authors clearly explain that a child is considered stunted if their HAZ is below -2 SD, and severely stunted if their HAZ is below -3 SD. However, there may also be a category for children who are not stunted, likely those with a HAZ of -2 SD and above. Did you categorize the variable into three groups? If you only used two categories to make it binary, how were these operationalized? Please provide a clear explanation in the text.
- Surprisingly, the entire methodology section is completely missing the sampling process and sample size.
- In the data analysis section, the authors should revise the sentence 'Descriptive statistics were used to summarise factors influencing stunting…' to ensure the correct statistical language is used. Consider revising it to: ‘Descriptive statistics were used to summarize the data on stunting, while the factors influencing stunting were analyzed using appropriate statistical methods’ etc.
- The authors state that ‘A chi-square test was used to compare observed results with expected results.’ Please explain why this was done and what you aimed to achieve. The correct application of the chi-square test, in this specific case, should have been to assess the association between stunting and each of the independent variables, as an intermediate level of data analysis (prior to multivariate analysis) for identifying factors associated with stunting. This applies if all variables were analyzed as categorical. If any variables were continuous, tests such as the t-test (e.g., comparing the mean age of stunted and non-stunted children) or analysis of variance (ANOVA) should have been mentioned if they were applied.
- Since one of the primary objectives of this study was to assess stunting trends from 1991/92 to 2022, the authors should specify any trend tests they performed in the methods section and present their findings on whether the trend was statistically significant in the results section.
- The authors state that ‘… univariate and multilevel binary logistic regression model to assess the factors influencing stunting in 2022.’ The authors should provide a clear justification for using multilevel binary logistic regression model. This should be done considering the fact that the formal analysis of DHS data requires the application of sampling weights, ensuring due consideration of Primary Sampling Units (PSUs), Clusters, and related parameters. Why then did the authors use multilevel analysis instead of adhering to the established approach?
- In using the multilevel model in this study, how many levels were included, and why were they chosen? What were those levels (e.g., household, community, PSU, region etc.), and what assumptions were made about them?
- Please change the subtitle ‘*Empirical analysis’* to ‘*Inferential analysis*’.
- Stepwise approaches, such as backward elimination and forward selection, are sometimes criticized for over-relying on statistical software to make decisions for you. A more effective method is to select variables based on their relevance to the research question, as informed by the literature. Then, at the data analysis stage, testing variables individually using methods like the likelihood ratio test can help ensure their appropriateness for inclusion in the model. So, why did you use both backward and forward elimination methods? Where did you use each method and how did the two inform each other in relation to the objectives of this study? Which cut-off points or criteria did you use to add or remove variables from a model?
- It is also stated in the data analysis section that “…to arrive at the final model for statistically significant levels, conditional on the p-value being greater than 5 percent (p > 0.05).” This statement contradicts statistical principles. Statistical significance is concluded when the associated p-value is less than 0.05 (p < 0.05), indicating that the observed relationship is unlikely to be due to chance, with a significance level (alpha) set at 5%.
- Please specify the statistical software used in this analysis.
- The authors state under ethical considerations section that “Prior to data collection, the Institutional Review Board (IRB) and Tanzania’s National Medical Research Committee approved the study protocol and DHS survey questionnaires.”
- Depending on whether the study used the DHS data as secondary or primary; is the ‘data collection’ referred to here referring to what the authors did themselves or it is a description of what the DHS Program went through in the process?
- Which IRB are the authors referring to? The one with the National Institute for Medical Research (NIMR)? Please specify.
- Is “National Medical Research Committee” existing in Tanzania? I am aware of the Medical Research Coordinating Committee (MRCC) of NIMR.
- What is described as “study protocol” who wrote it; the authors or the DHS Program?
- Overall, if what is described here is what the DHS Program (not the authors) went through to collect the DHS data, the authors can skip this or describe briefly and clearly that DHS data collection typically follows IRB approval and strict ethical standards, ensuring informed consent, privacy, and confidentiality. This process is carried out in collaboration with national governments and local institutions.

**Results**

- I expected the results section to begin with a brief profile of the respondents, providing a clear picture of their sociodemographic characteristics and other relevant details of the respondents.
- Why not change “Elisaria et al.'s (2020) qualitative research in the Njombe region found that… ” to “A qualitative research in Njombe region by Elisaria et al (2020) found that… ”?
- Since results and discussion appear to be separate sections in this study, I did not expect to see any references in the results section.
- In Table 1, the percentages in the ‘Total’ column should add up to 100% for each category, based on the data presented in the ‘Not Stunted’ and ‘Stunted’ columns. Currently, the percentages under the ‘Total’ column are unclear. For example, what does the total of 35.3% mean for Lakes? What does 2.9% mean for Zanzibar? Additionally, is this table weighted or unweighted?
- In Table 2, the results of the multilevel analysis are shown, including both crude and adjusted odds ratios. This is good. However, a multilevel analysis has two components: fixed effects and random effects. The current presentation only includes the fixed effects, with the random effects missing. This ties back to the earlier question about the number of levels considered in the analysis and the rationale behind their inclusion. To strengthen the results, the Intraclass Correlation Coefficients (ICCs) for each level should be reported, as they help demonstrate the importance of each level and validate the assumptions made regarding the levels.

**Discussion**

- The results section should focus solely on presenting the results, with additional details and references moved to the discussion section. The discussion section, as it currently stands, feels shallow and lacks sufficient details; for example, the relationship between caesarean section and stunting and the likely underlying mechanisms is not discussed at all. etc.

**Conclusion and Recommendations**

- This section is written in broad terms, without clearly specifying how individual factors relate to stunting (i.e., effect magnitude and direction). For instance, the recommendation to "eliminate structural obstacles including unequal roles" is not directly supported by the study's findings, which mainly focused on SES and other demographic factors, rather than structural issues like unequal roles. Similarly, the statement about current regulations emphasizing diet and health education for mothers over economic empowerment is not backed by the study, as it did not assess the effectiveness of current programs in either area. To strengthen the conclusion, the study should more closely align the findings with the proposed actions, particularly emphasizing the role of socioeconomic disparities and advocating for a targeted, evidence-based approach to addressing stunting in Tanzania.

Abbreviations

- The long form of ‘TDHS’ should be ‘Tanzania Demographic and Health Survey’, not ‘Tanzania Demographic Health Survey’
