# Supplementary material for: Thirty years of declining stunting in Tanzania: Trends and ongoing challenges
Source: PLoS One. 2025 Jul 29;20(7):e0327779. doi: 10.1371/journal.pone.0327779 (PMC12306788; doi:10.1371/journal.pone.0327779)
Supplement: Attachment — Submitted filename: Point response to the comments 07.02.2025.docx [file pone.0327779.s003.docx]

Point response to the comments

| # | Comment | Response | Page # |
| --- | --- | --- | --- |
| Editorial - Journal requirements: | | |  |
| 1 | Thank you for stating the following financial disclosure: “This project was funded with UK International Development from the UK government and New Zealand’s Ministry of Foreign Affairs and Trade (MFAT) contract PO number 10087; however, the views expressed do not necessarily reflect the UK or New Zealand’s government official policies”  Please state what role the funders took in the study. If the funders had no role, please state: "The funders had no role in study design, data collection and analysis, decision to publish, or preparation of the manuscript."  If this statement is not correct you must amend it as needed.  Please include this amended Role of Funder statement in your cover letter; we will change the online submission form on your behalf. | Thank you for this observation we have included the following statement “The funders had no role in study design, data collection and analysis, decision to publish, or preparation of the manuscript." | Page 29 Acknowledgements section |
| 2 | In the online submission form, you indicated that [The dataset(s) supporting the conclusions of this article is owned by the Demographic and Health Surveys (DHS) Program, ICF, 530 Gaither Road, Suite 500, Rockville, MD 20850, USA and available upon request.].  All PLOS journals now require all data underlying the findings described in their manuscript to be freely available to other researchers, either 1. In a public repository, 2. Within the manuscript itself, or 3. Uploaded as supplementary information.  This policy applies to all data except where public deposition would breach compliance with the protocol approved by your research ethics board. If your data cannot be made publicly available for ethical or legal reasons (e.g., public availability would compromise patient privacy), please explain your reasons on resubmission and your exemption request will be escalated for approval. | We appreciate this feedback. The Tanzania Demographic Health Survey Data supporting the analysis is available, however, will have to inquire from DHS Program whether is allowed to be uploaded on other websites. I have amended the text as follows:  The dataset(s) supporting the conclusions of this article is owned by the Demographic and Health Surveys (DHS) Program, ICF, 530 Gaither Road, Suite 500, Rockville, MD 20850, USA and available upon request. To request and access the required data, visit the official webpage https://dhsprogram.com/, register, and await notification along with an official authorization letter from the DHS Program team. Further information may be requested via email from Bridgette Wellington (Data Archivist, DHS Program; Bridgette.Wellington@icf.com; & cc: archive@dhsprogram.com). The datasets used and/or analysed during the current study available from the corresponding author on reasonable request. | Page 30 Availability of data and material |
| 3 | We note that Figure 1 in your submission contain [map/satellite] images which may be copyrighted. All PLOS content is published under the Creative Commons Attribution License (CC BY 4.0), which means that the manuscript, images, and Supporting Information files will be freely available online, and any third party is permitted to access, download, copy, distribute, and use these materials in any way, even commercially, with proper attribution. For these reasons, we cannot publish previously copyrighted maps or satellite images created using proprietary data, such as Google software (Google Maps, Street View, and Earth). For more information, see our copyright guidelines: http://journals.plos.org/plosone/s/licenses-and-copyright.  We require you to either (1) present written permission from the copyright holder to publish these figures specifically under the CC BY 4.0 license, or (2) remove the figures from your submission: | We appreciate this observation and have decided to remove the figures from the submission. |  |
|  | Overall |  |  |
|  | The title "Thirty Years of Stunting in Tanzania" may not fully capture the significant positive trends observed in the study, particularly the decline in stunting rates from 50% in 1991/92 to 30% in 2022. To better reflect these encouraging findings, the title could emphasize the reduction in stunting, such as "Declining Stunting Rates in Tanzania: A 30-Year Overview" or "Tracking 30 Years of Declining Stunting in Tanzania." This would more clearly communicate the positive trend of stunting reduction over the three decades. However, it is also important to highlight that despite this progress, challenges remain, and continued efforts are necessary to further reduce stunting, especially in underprivileged communities. A revised title like "Thirty Years of Declining Stunting in Tanzania: Trends and Ongoing Challenges" could better capture both the progress made and the need for targeted interventions to address remaining disparities. | We see reviews concern and we have amended the title now reads “*Thirty Years of Declining Stunting in Tanzania: Trends and Ongoing Challenge*s" | Page 1 |
|  | Abstract |  |  |
|  | Please ensure the methods section of the abstract specifies the years of the TDHS datasets used in the study. | Years of the TDHS have been added in the abstract (1991-92; 1996; 1999; 2004-05; 2010; 2015-16 and 2022) | Page 2, Abstract - Methods section |
|  | In the methods or results sections of the abstract, the sample size(s) should be indicated. | Text has been amended and now reads “……Descriptive statistics were calculated using the Tanzania Demographic and Health Survey (TDHS) data from 42,408 under-five children spanning 1991–1992 (n = 6,585), 1996 (n = 5,438), 1999 (n = 2,555), 2004–2005 (n = 7,230), 2009–2010 (n = 6,792), 2015–2016 (n = 9,001), and 2022 (n = 4,807).…….” | Page 2, Abstract - Methods section |
|  | In the results section of the abstract, the term "SES" already stands for "socioeconomic status," so the use of "SES status" appears redundant and should be revised to simply "SES". | The term SES status has been revised and have deleted …” …status…” … |  |
|  | Revise “… was significant associated with risk of being stunting” to something like “… was significantly associated with the risk of being stunted”. | Text has been amended and now a text read “…. was significantly associated with the risk of being stunted ….” | Page 2 & 3, results section |
|  | The conclusion section of the abstract needs revision. Unnecessary information can be reduced to make it concise. For example, the first two sentences could be revised to something like “Stunting remains a major challenge in Tanzania, driven by factors at the maternal, family, community, and child levels.” | Thank you for the proposition and we have amended the text including adding the proposed sentence. Stunting remains a major challenge in Tanzania, driven by factors at the maternal, family, community, and child levels. | Page 3, conclusion |
|  | The keyword “Demographic Health Survey” should be “Demographic and Health Survey”, referring to DHS. | We have amended the keyword and included “Demographic and Health Survey”. | Whole document |
|  | Introduction |  |  |
|  | Revise the first sentence of the introduction to something like “In 2022, an estimated 148.1 million children under the age of five globally were classified as stunted, ...” | Thank you for the proposed sentence. Now the first sentence of the introduction reads “In 2022, an estimated 148.1 million children under the age of five globally were classified as stunted”. | Page 4 |
|  | “Demographic Health Surveys” should be “Demographic and Health Surveys” | We appreciate your feedback. Changes have been made to the document. | Whole document |
|  | The sentence on page 3 “…data collected from 1991/92-2022 indicates the country had a decrease in stunting from 50% in 1991/92 to 30% [2] …” should indicate when the 30% was the case. In the abstract, it is stated that 30% was the case in 2022. Please revise accordingly. | Thank you for this note. The text has been revised to include additional information regarding the 30% case, which relates to 2022. | Page 3 |
|  | Please revise the entire manuscript to correct any grammar and sentence structure issues. For example, the sentence “The World Bank and UNICEF agree that heightened efforts needed to reach the global goal of reducing the number of children suffering from stunting to 89 million by 2030 [1]” seems to be missing the word “are,” which affects the sentence’s clarity and flow. | The entire manuscript has been reviewed by a native English speaker and corrected for grammar. | Whole document |
|  |  |  |  |
|  | Methods |  |  |
|  | Did the authors use the Tanzania DHS data, 1991/92 – 2022 as secondary data or primary data? | Authors used Tanzania DHS data from 1991/92 to 2022 as secondary data; no primary data were collected. |  |
|  | How was the data accessed? If the standard procedure of accessing such data through the DHS Program (https://dhsprogram.com/) was (or wasn’t) used, it should be made explicit. | The standard procedure for accessing such data through the DHS programme was followed, as mentioned in the manuscript under the data access sub-heading. | Page 7 |
|  | Consider adding more subtitles. For example, you have indicated in the data source that this study was based on multiple cross-sectional data points. This can appear under a separate subtitle – Study design – and indicate that this was a repeated cross-sectional study or serial cross-sectional study. | Three new subsections have been added in the methods section, Study Design, Data Access and TDHS Sampling of Households | Page 5 & 7 |
|  | Then we need to see how many data points were used over the mentioned period, 1991/92 – 2022. | A new subsection has been added in the methods section showing data points over the mentioned period. The text reads as …… “The TDHS employed a random sampling method that considered population density into account to gather data from all administrative regions of the country. Data of 42,408 under-five children had had nutrition related variables. The waves of TDHS had different sample size, 1991–1992 (n = 6,585), 1996 (n = 5,438), 1999 (n = 2,555), 2004–2005 (n = 7,230), 2009–2010 (n = 6,792), 2015–2016 (n = 9,001), and 2022 (n = 4,807) …..” | Page 2 – abstract  Page 7 - methods section |
|  | Please clarify the variables used, both dependent and independent. For instance, the authors clearly explain that a child is considered stunted if their HAZ is below -2 SD, and severely stunted if their HAZ is below -3 SD.  However, there may also be a category for children who are not stunted, likely those with a HAZ of -2 SD and above. Did you categorize the variable into three groups? If you only used two categories to make it binary, how were these operationalized? Please provide a clear explanation in the text. | The primary outcome of interest was stunting, defined as a height-for-age measurement that is more than two standard deviations below the mean of the reference population. Then the variables was categorize the variable into two groups, stunted vs non-stunted. |  |
|  | Surprisingly, the entire methodology section is completely missing the sampling process and sample size. | A new subsection has been added (‘TDHS Sampling of Households’) in the methods section showing data points over the mentioned period. The text reads as …… “The TDHS employed a random sampling method that considered population density into account to gather data from all administrative regions of the country. Data of 42,408 under-five children had had nutrition related variables. The waves of TDHS had different sample size, 1991–1992 (n = 6,585), 1996 (n = 5,438), 1999 (n = 2,555), 2004–2005 (n = 7,230), 2009–2010 (n = 6,792), 2015–2016 (n = 9,001), and 2022 (n = 4,807)…..” | Page 2 – abstract  Page 7 - methods section |
|  | In the data analysis section, the authors should revise the sentence 'Descriptive statistics were used to summarise factors influencing stunting…' to ensure the correct statistical language is used. Consider revising it to: ‘Descriptive statistics were used to summarize the data on stunting, while the factors influencing stunting were analyzed using appropriate statistical methods’ etc. | The sentence has been revised and adapted the proposed one ….. “…Descriptive statistics were used to summarize the data on stunting, while the factors influencing stunting were analyzed using appropriate statistical methods...” | Page 7 |
|  | The authors state that ‘A chi-square test was used to compare observed results with expected results.’ Please explain why this was done and what you aimed to achieve. The correct application of the chi-square test, in this specific case, should have been to assess the association between stunting and each of the independent variables, as an intermediate level of data analysis (prior to multivariate analysis) for identifying factors associated with stunting. This applies if all variables were analyzed as categorical. If any variables were continuous, tests such as the t-test (e.g., comparing the mean age of stunted and non-stunted children) or analysis of variance (ANOVA) should have been mentioned if they were applied. | We have modified the text as all the variables presented in Table 2 were categorical. A chi-square test was used to assess the association between stunting and each of the independent variables for identifying factors associated with stunting. | Page 14 - 16 |
|  | Since one of the primary objectives of this study was to assess stunting trends from 1991/92 to 2022, the authors should specify any trend tests they performed in the methods section and present their findings on whether the trend was statistically significant in the results section. | We have added 95% confidence intervals (CI) within figure 1 | Figure 1 A: Thirty Years Stunting 1991/92 – 2022 |
|  | The authors state that ‘… univariate and multilevel binary logistic regression model to assess the factors influencing stunting in 2022.’ The authors should provide a clear justification for using multilevel binary logistic regression model. This should be done considering the fact that the formal analysis of DHS data requires the application of sampling weights, ensuring due consideration of Primary Sampling Units (PSUs), Clusters, and related parameters. Why then did the authors use multilevel analysis instead of adhering to the established approach?  In using the multilevel model in this study, how many levels were included, and why were they chosen? What were those levels (e.g., household, community, PSU, region etc.), and what assumptions were made about them? | As levels were not considered during the regression analysis, we have amended the term 'Multilevel' to 'multivariate'. The revised statement now refers to univariate and multivariate logistic regression models to assess the factors influencing stunting in 2022. | Page 16 |
|  | Please change the subtitle ‘Empirical analysis’ to ‘Inferential analysis’. | Empirical analysis has been changed to ‘Inferential analysis’. | Page 8 |
|  | Stepwise approaches, such as backward elimination and forward selection, are sometimes criticized for over-relying on statistical software to make decisions for you. A more effective method is to select variables based on their relevance to the research question, as informed by the literature. Then, at the data analysis stage, testing variables individually using methods like the likelihood ratio test can help ensure their appropriateness for inclusion in the model. So, why did you use both backward and forward elimination methods? Where did you use each method and how did the two inform each other in relation to the objectives of this study? Which cut-off points or criteria did you use to add or remove variables from a model? | The selection of the variables for inclusion in the whole analysis was informed by the literature review and availability of the variable within the Tanzania DHS.  We used both backward and forward elimination, as documented elsewhere. The decision to include or exclude certain variables was informed by the changes observed after performing the model (odds ratio, standard error, and p-values < 0.05). |  |
|  | It is also stated in the data analysis section that “…to arrive at the final model for statistically significant levels, conditional on the p-value being greater than 5 percent (p > 0.05).” This statement contradicts statistical principles. Statistical significance is concluded when the associated p-value is less than 0.05 (p < 0.05), indicating that the observed relationship is unlikely to be due to chance, with a significance level (alpha) set at 5%. | We have amended the text, as it was overlooked; we meant that the p-value is less than 0.05. | Page 8 |
|  | Please specify the statistical software used in this analysis. | STATA Version 15 was used in the data management and analysis. | Page 8 |
|  | The authors state under ethical considerations section that “Prior to data collection, the Institutional Review Board (IRB) and Tanzania’s National Medical Research  Committee approved the study protocol and DHS survey questionnaires.”  Depending on whether the study used the DHS data as secondary or primary; is the ‘data collection’ referred to here referring to what the authors did themselves or it is a description of what the DHS Program went through in the process?  Which IRB are the authors referring to? The one with the National Institute for Medical Research (NIMR)? Please specify.  Is “National Medical Research Committee” existing in Tanzania? I am aware of the Medical Research Coordinating Committee (MRCC) of NIMR.  What is described as “study protocol” who wrote it; the authors or the DHS Program?  Overall, if what is described here is what the DHS Program (not the authors) went through to collect the DHS data, the authors can skip this or describe briefly and clearly that DHS data collection typically follows IRB approval and strict ethical standards, ensuring informed consent, privacy, and confidentiality. This process is carried out in collaboration with national governments and local institutions. | The use of this data was approved by the Demographic and Health Surveys (DHS) Program, ICF, following our request with the data analysis concept. During the Tanzania DHS, the protocols and data collection procedures were approved by the relevant authorities in mainland Tanzania and Zanzibar. These included the National Institute of Medical Research (NIMR), the Zanzibar Medical Research Ethical Committee (ZAMREC), the Institutional Review Board of ICF International, and the Centres for Disease Control and Prevention in Atlanta. In the data collection procedures, all participants were asked to provide verbal informed consent after the consent statement was read to them, which emphasised the voluntary nature of the survey. Interviews were conducted in the private conditions afforded by the environments encountered. Confidentiality was maintained by ensuring that respondents' names were not written in the data collection tools, thus preserving their anonymity. | Page 8 |
|  | Results |  |  |
|  | I expected the results section to begin with a brief profile of the respondents, providing a clear picture of their sociodemographic characteristics and other relevant details of the respondents. | A new table has been introduced to present the basic information regarding the respondents' profiles (see Table 1: profile of the respondents). | Table 1 page 9 - 10 |
|  | Why not change “Elisaria et al.'s (2020) qualitative research in the Njombe region found that… ” to “A qualitative research in Njombe region by Elisaria et al (2020) found that… ”? | We have amended the text and change it to ….. “A qualitative research in Njombe region by Elisaria et al (2020) found that….” | Page 27 |
|  | Since results and discussion appear to be separate sections in this study, I did not expect to see any references in the results section. | We have revised whole results section and separated the discussion from the results section | Page 9 - 19 |
|  | In Table 1, the percentages in the ‘Total’ column should add up to 100% for each category, based on the data presented in the ‘Not Stunted’ and ‘Stunted’ columns. Currently, the percentages under the ‘Total’ column are unclear. For example, what does the total of 35.3% mean for Lakes? What does 2.9% mean for Zanzibar? Additionally, is this table weighted or unweighted? | As a new table has been introduced earlier, this will now be Table 2.  We have chosen to remove the column total to prevent potential confusion. | Page 14 – 16 |
|  | In Table 2, the results of the multilevel analysis are shown, including both crude and adjusted odds ratios. This is good. However, a multilevel analysis has two components: fixed effects and random effects. The current presentation only includes the fixed effects, with the random effects missing. This ties back to the earlier question about the number of levels considered in the analysis and the rationale behind their inclusion. To strengthen the results, the Intraclass Correlation Coefficients (ICCs) for each level should be reported, as they help demonstrate the importance of each level and validate the assumptions made regarding the levels. | The terminology has been revised from "multilevel" to "multivariate" as levels were not considered during the analysis. | Page 16 |
|  | Discussion |  |  |
|  | The results section should focus solely on presenting the results, with additional details and references moved to the discussion section. The discussion section, as it currently stands, feels shallow and lacks sufficient details; for example, the relationship between caesarean section and stunting and the likely underlying mechanisms is not discussed at all. etc. | All citations and relevant information have been transferred from the results section to the discussion section. The discussion has been revised accordingly. | Page 19 - 27 |
|  | Conclusion and recommendations |  |  |
|  | This section is written in broad terms, without clearly specifying how individual factors relate to stunting (i.e., effect magnitude and direction). For instance, the recommendation to "eliminate structural obstacles including unequal roles" is not directly supported by the study's findings, which mainly focused on SES and other demographic factors, rather than structural issues like unequal roles. Similarly, the statement about current regulations emphasizing diet and health education for mothers over economic empowerment is not backed by the study, as it did not assess the effectiveness of current programs in either area. To strengthen the conclusion, the study should more closely align the findings with the proposed actions, particularly emphasizing the role of socioeconomic disparities and advocating for a targeted, evidence-based approach to addressing stunting in Tanzania. | Conclusion has been revised | Page 28 |
