# Supplementary material for: Thirty years of declining stunting in Tanzania: Trends and ongoing challenges
Source: PLoS One. 2025 Jul 29;20(7):e0327779. doi: 10.1371/journal.pone.0327779 (PMC12306788; doi:10.1371/journal.pone.0327779)
Supplement: Attachment — Submitted filename: Reviwers comments PLOS.docx [file pone.0327779.s004.docx]

Abstract

Revised the methods section of the abstract, please mention what analysis you have used. Revised the results section please note that Odds ratios less than 1 are not associated with high risk. Birth interval of 24 to 47 months, secondary education and above and higher socioeconomic status had odds less than one. The conclusion section is not based on the study findings. The last sentence is not relevant to study findings.

Methods

Data analysis

Please revised the data analysis section. No need to add the sentence for hypothesis. I am not sure that the authors had use the weight when doing regression. Please note that we should weight while doing the logistic regression for demographic surveys.

Results

“Not only does stunting vary by region of residence, but there are also important and well-established differences by child characteristics.” Please don’t comments in the results section.
“To determine the statistical significance of the differences described above, Table 2 presents bivariate

regression results of 2022 stunting rates on various characteristics associated with the risk of being

stunted, including demographic characteristics such as age, place of residence, child gender, zone,

mother’s educational status, and wealth index quintile”. Please revise this text, it is not regression you are doing. It is chisquare test.

Revised the results section of the manuscript. Please note that Odds ratios less than 1 are not associated with high risk. Birth interval of 24 to 47 months, secondary education and above and higher socioeconomic status had odds less than one and it is associated with low risk. Please do interpretation differently. The graph can be better, use similar minimum and maximum points for each graph (Figure 1 to Figure 2), better to remove the lines in the graph. There is no overall legend in Figure 2 A. Be consistent. In table 1, 2 and table 3, p-value is mentioned as 0.000. Replace it by <0.005 or <0.001 where relevant. Use complete word don’t use Bir order or Bir interval, Give units for child age in table 2 and table 3.

Discussion

Replace the word synthesis. The discussion is too long and some sentences are not relevant to study findings. Please narrow down the discussion. Highlights only important and significant factors associated with outcomes.

I am not sure whether we need abbreviations in the manuscript. Please remove it.
