# Supplementary material for: Thirty years of declining stunting in Tanzania: Trends and ongoing challenges
Source: PLoS One. 2025 Jul 29;20(7):e0327779. doi: 10.1371/journal.pone.0327779 (PMC12306788; doi:10.1371/journal.pone.0327779)
Supplement: Attachment — Submitted filename: Point response to the comments 6.6.2025.docx [file pone.0327779.s005.docx]

**Point response to the comments**

| **#** | **Comment** | **Response** | **Page #** |
| --- | --- | --- | --- |
| Editorial feedback | | |  |
| 1 | Submit a filled-out STROBE checklist for the journal's files | Thank you for this observation; we have included the STROBE checklist. | Supplementary files |
| 2 | Please provide a written response to Reviewer 3: I was not able to find this in the online files, but I apologize in advance if it is there and I missed it.  Please provide responses to Reviewer 3's comments on Revision 1 (I have not been able to find these) | Reviewer 3's comments were addressed, and the current email dated 22 May 2025 indicates that all comments have been addressed.  *1. If the authors have adequately addressed your comments raised in a previous round of review and you feel that this manuscript is now acceptable for publication, you may indicate that here to bypass the “Comments to the Author” section, enter your conflict of interest statement in the “Confidential to Editor” section, and submit your "Accept" recommendation.*  *Reviewer #1: (No Response)*  *Reviewer #3: All comments have been addressed* |  |
| 3 | Please follow Reviewer 3's request to shorten the discussion. Good practice is to state a point based on your results and succinctly compare to other articles in the literature.  Please make the Discussion more succinct, as Reviewer 3 requested | Thank you for this suggestion; the discussion has been significantly revised and shortened. We hope the revised version makes the main take away points and the links to the literature clearer. | Page 20 – 22  Line 340 - 415 |
| 4 | Please also remove the list of abbreviations. This is not the style for this journal. You simply spell out the abbreviation on first use, and then after that just use the abbreviation. | The list of abbreviation has been deleted and we have made sure that all abbreviations are spelled out at time of first use. | Through the document |
| 5 | I believe that journal policy is to add line numbers to the submitted Manuscript to make it easier for review  I thought that PLOS required line numbers on a revised version. Please include these. | Continued line numbers have been added to the revised manuscript | Through the document |
| 6 | I have also provided a list of typos, as follows: |  |  |
|  | Abstract: suggest “maternal secondary education” rather than “secondary education” | We appreciate this observation. Text corresponding to the comments has been updated. | Page 3 line 47-48 |
|  | P5: top line: consider saying “only” 19% of children aged…..” |  | Page 5 line 94 |
|  | Section entitled “TDHS sampling of households”: In first sentence, consider saying “took” rather than “considered” |  | Page 7 line 157 |
|  | Table 1: number of ANC visits: one of these categories should be either “greater than or equal to” or “less than or equal to” (later it seems that the first category is “less than or equal to 4”; the same issue is observed in table 2 and table 3. | Table, 1 – 3, number of ANC visits, information has been updated to **>= 4 ANC visits** | Page 10, Table 1  Page 16, Table 2  Page 19, Table 3 |
|  | P13, section on stunting and diarrhoea: it would be helpful to specify the period referred to in terms of episodes of diarrhoea, i.e. the question asked by DHS | We appreciate this observation, and we have added information to reflect that the DHS data collection tool captured details on diarrhoea in the two weeks prior to the survey. | Page 5 line 104  Page 13 line 268  Page 15 Table 2  Page 18 Table 3 |
|  | P13: discussion of regions: given that the Abstract mentions Southwest Highlands and Southern Highlands specifically, it would be helpful to identify which regions belong in each of these Highland areas (Google didn’t seem to distinguish Southern Highlands and Southwestern, but indicated that Iringa, Mbeya and Ruvuma, all listed here, were part of the Southern Highlands) | Regions belonging to the South West Highlands include Katavi, Rukwa, Songwe, and Mbeya, while the Southern Highlands comprise Iringa, Njombe, and Ruvuma.  Text has been added at the bottom of Table 2 to list the regions | Page 16 line 310 - 311 |
|  | Table 3: birth order and birth interval should be spelled out in full | In Table 3, the term ‘bir’ has been spelled out in full ‘birth’ | Page 18 Table 3 |
|  | P22: reference to Qadri et al – suggest specify the country to which this study refers. | The discussion section has been modified, and the reference to Qadri et al. is no longer included in the revised version of the manuscript. |  |
|  | P23: I couldn’t find Mtongwa et al (2022) in the reference list, and in any case this reference should be given a numbered reference, not (2022). | We appreciate this observation. The discussion section has been modified, and shortened. | Page 20 – 22, line 340 - 405 |
|  | P23, second last line: “Ideally” should be deleted. |  |  |
|  | P24, last paragraph: delete first mention of “influence”. Should read “Child age was found to influence stunting”. |  |  |
|  | P25: don’t give date of publication for Karlsson et al, since that isn’t consistent with the style for this journal. |  |  |
|  | P25: first sentence of last paragraph: Suggest rephrasing as “The household factors include socio-economic status which was significantly associated with…” |  |  |
|  | P26, in first complete paragraph: capitalize Infant (in Infant and Young Child Feeding guidelines) |  |  |
|  | Section on Limitations: do you mean that “no new primary data were collected”? | Yes, indeed, no new primary data were collected. We clarified this. |  |
|  | General comments |  |  |
|  | Abstract  Revised the methods section of the abstract, please mention what analysis you have used. Revised the results section please note that Odds ratios less than 1 are not associated with high risk. Birth interval of 24 to 47 months, secondary education and above and higher socioeconomic status had odds less than one. The conclusion section is not based on the study findings. The last sentence is not relevant to study findings. | Revision has been made to be more specific to the results.  **Results**:  *Over the previous three decades, stunting rates in boys under five have consistently been 4-7 percentage points (ppt) higher than those in girls of the same age. The prevalence of stunted children was greater in households with the lowest socioeconomic status (SES) (38.6%) compared to those with the highest SES (12.6%). Similar to the trend analysis, a multivariable regression analysis found that residency in the Southern Highlands (AOR = 2.368; 95% CI: 1.746-3.212, p < 0.001), male children (AOR = 1.583 [95% CI: 1.349 - 1.858], p < 0.001), low birth weight (AOR = 3.639 [95% CI: 2.279 - 5.810], p < 0.001), maternal exposure to alcohol (AOR = 1.440 [95% CI: 1.057 – 1.963], p < 0.05), and unimproved sanitation facilities (AOR = 1.345 [95% CI: 1.055 – 1.714], p < 0.05) were significantly associated with a higher risk of stunting. In contrast, a birth interval of 24 to 47 months (AOR = 0.762 [95% CI: 0.598 - 0.969], p < 0.001), a high level of maternal education (AOR = 0.715 [95% CI: 0.530 – 0.963], p < 0.05), and high socioeconomic status (AOR = 0.268 [95% CI: 0.178 – 0.403], p < 0.001) were significantly associated with a lower risk of stunting.*  **Conclusion**  *Although child stunting has decreased in the country, it remains a major challenge in Tanzania, driven by factors such as residing in the Southern Highlands, child and maternal issues, and household economic and environmental factors. To combat stunting and eliminate structural obstacles, including the empowerment of marginalised groups, a multisectoral strategy is required. Furthermore, current regulations and standards place more emphasis on educating mothers about diet and health than on empowering them economically.* | Page 2 – 3, line 22 - 58 |
|  | Data analysis    Please revised the data analysis section. No need to add the sentence for hypothesis. I am not sure that the authors had use the weight when doing regression. Please note that we should weight while doing the logistic regression for demographic surveys. | All the analysis was weighted, this has now been clarified, thank you for pointing out this ambiguity. | Page 8 line 178 - 181 |
|  | Results  “Not only does stunting vary by region of residence, but there are also important and well-established differences by child characteristics.” Please don’t comments in the results section. “To determine the statistical significance of the differences described above, Table 2 presents bivariate regression results of 2022 stunting rates on various characteristics associated with the risk of being  stunted, including demographic characteristics such as age, place of residence, child gender, zone,  mother’s educational status, and wealth index quintile”. Please revise this text, it is not regression you are doing. It is chi-square test.  Revised the results section of the manuscript. Please note that Odds ratios less than 1 are not associated with high risk. Birth interval of 24 to 47 months, secondary education and above and higher socioeconomic status had odds less than one and it is associated with low risk. Please do interpretation differently. The graph can be better, use similar minimum and maximum points for each graph (Figure 1 to Figure 2), better to remove the lines in the graph.  There is no overall legend in Figure 2 A. Be consistent.  In table 1, 2 and table 3, p-value is mentioned as 0.000. Replace it by <0.005 or <0.001 where relevant. Use complete word don’t use Bir order or Bir interval, Give units for child age in table 2 and table 3. | Thank you for this observation, text has been revised.  We have in fact significantly reduced the section on multivariable regression analysis to avoid repetition to what was already discussed earlier in the text. The revised section now serves the purpose to confirm what factors remain significant and which ones do not, after controlling for confounding factors. The discussion section picks up the core findings again, hence we thought we would remove a significant amount of repetition by shortening this section. We hope the reviewers will agree.  We feel the lines should be maintained for each graph (Figure 1 to figure 2)  Figure 2 A has been amended to include overall stunting  Text within the tables has been amended. | Page 14 line 281-282  Table 1, 2 and table 3 |
|  | Discussion  Replace the word synthesis. The discussion is too long and some sentences are not relevant to study findings. Please narrow down the discussion. Highlights only important and significant factors associated with outcomes. | Thank you for this suggestion; the discussion has been significantly revised, shortened and made more relevant to the core findings. | Page 20 – 22, line 340 - 405 |
|  | I am not sure whether we need abbreviations in the manuscript. Please remove it. | List of abbreviations have been removed |  |
|  |  |  |  |
|  | Reviewer 1 |  |  |
| 1 | I appreciate the authors for making substantial revisions to the manuscript in response to my earlier comments. Their efforts to improve the clarity and presentation of the study are commendable. However, my major concern is that the term "multilevel" has been replaced with "multivariate." If this change was merely in terminology, without a corresponding revision in the analysis, then the approach remains incorrect. In revising the analysis, the authors should first declare the data as 'survey' in design through the 'svyset' command in Stata (as mentioned, Stata was used). I think it takes this form... "svyset [pweight=v005], psu(v021) strata(v022)". Following this, each syntax for both descriptive and multivariate analyses should begin with the 'svy:' prefix to account for the survey design.  Currently, the analysis does not account for the inherent clustering of DHS data. Given the survey design, it is essential to either conduct a weighted analysis that accounts for stratification and primary sampling units (PSUs), or to use a multilevel approach to properly address the hierarchical structure of the data. Ignoring clustering can lead to incorrect standard errors and potentially biased estimates.  I strongly encourage the authors to either incorporate survey weights and adjust for clustering, or implement a multilevel model that aligns with the hierarchical nature of the data. Clarifying these methodological choices in the manuscript would greatly strengthen the rigor and validity of the findings. | All the analysis applied sample weighting as advised by TDHS and, indeed, as outlined in the reviewer comment. We have now clarified this in the text. Thank you very much for this important comment. | Page 8 line 178 - 181 |
| 2 | Consider using 'multivariable' instead of 'multivariate' because multiple predictors or covariates are being associated with a single outcome - stunting. | The term multivariable has been adapted in the manuscript | page 2 line 34 - 35 and line 41  Page 8 line 172 & 175  Page 17 line 312 - 317  Page 18 line 335 & 337 |
| 3 | Consider marking p-values of '0.000' as 'p<0.001' | P values have been amended | Page 2 - 3 line 38 - 50  Page 15 - 16 Table 2  Page 18 - 19 Table 3 |
|  | Reviewer 3 |  |  |
| 1 | Thank you for your valuable feedback and insightful comments. The manuscript needs to revised sections to enhance clarity, ensuring logical flow and coherence throughout the manuscript. The methodology to provide additional details on data analysis. The discussion needs to strengthened the discussion by incorporating relevant literature. The study adheres to ethical guidelines, and necessary approvals have been obtained since the study was based on secondary data analysis. | Thank you for this suggestion; the methodology has been improved and the discussion has been significantly revised and shortened. | Page 20 – 22, line 340 - 405 |
